# Isolation and Total Synthesis of PM170453, a New Cyclic Depsipeptide Isolated from *Lyngbya* sp.

**DOI:** 10.3390/md22070303

**Published:** 2024-06-28

**Authors:** Rogelio Fernández, Marta Pérez, Alejandro Losada, Silvia Reboredo, Asier Gómez-San Juan, María Jesús Martín, Andrés Francesch, Simon Munt, Carmen Cuevas

**Affiliations:** Research and Development, PharmaMar S.A., Avda. de los Reyes 1, Pol. In. La Mina, 28770 Madrid, Spainccuevas@pharmamar.com (C.C.)

**Keywords:** cyclic depsipeptide, cyanobacteria, *Lyngbya*, cytotoxicity, PD-1/PD-L1 inhibition, isolation, structure elucidation, total synthesis

## Abstract

In our continuing search for biologically active new chemical entities from marine organisms, we have isolated a new cyclic depsipeptide, PM170453 (**1**), from a cyanobacterium of the genus *Lyngbya* sp., collected in the Indo-Pacific Ocean. Structure elucidation of the isolated compound was determined by spectroscopic methods including MS, ^1^H, ^13^C and 2D-NMR. To solve the supply problem for **1** and progress pharmaceutical development, the total synthesis of **1** that involves a total of 20 chemical steps in a convergent process was carried out. Its in vitro cytotoxic activity against four human tumor cell lines, as well as the inhibition of the interaction between the programmed cell death protein 1 PD-1 and its ligand PD-L1 were also evaluated.

## 1. Introduction

The programmed cell death 1 receptor (PD-1) and its ligand programmed cell death ligand 1 (PD-L1) are immune checkpoint proteins found on the cell surface of various immune cells. PD-L1 is also expressed in a variety of human cancers. Under physiological conditions, their interaction results in cell immune suppression, but in cancer cells this interaction results in the escape of immune detection. However, this can be reversed by blocking PD-1’s interaction with PD-L1 [1]. Marine cyanobacteria have emerged as a promising source of bioactive compounds with therapeutic and pharmacological potential [2]. In this context, cyclic peptides have garnered particular interest due to their structural diversity and biological activity. In particular, the *Lyngbya* genus, a strain of Cyanobacteria belonging to the family Oscillatoriacea Engler, has provided different metabolites with biological effects, and their unusual activities as immunosuppressants are remarkably interesting [3,4]. Yanucamides, for instance, are natural depsipeptides isolated from an assemblage of the marine Cyanobacteria *Lyngbya majuscula* and *Schizothrix* species [5]. These structures exhibit strong brine shrimp toxicity, although further biological activities have not been tested so far. Later, the total synthesis and stereochemical revision of yanucamide A was reported [6]. In our continuing search for biologically active compounds from marine organisms, we have isolated a new cyclic depsipeptide, PM170453 (**1**), from a *Lyngbya* sp. specimen, collected off the coast of Buru Island, the third largest island within the Maluku Islands of Indonesia. Initially the crude extract of this specimen showed hints of inhibition of the PD-1 receptor activity, which prompted us to further fractionate it. Structurally, compound **1** has similarity to several cyclic peptides such as yanucamides [5], dudawalamides [7], kulolides [8] and viequeamides [9]. The structure of **1** was determined using different spectroscopic methods such as 1D- and 2D-NMR, and the molecular formula was confirmed by HRESITOFMS. It contains three amino acids (β-alanine, *N*-methyl-phenylalanine and proline) and a 2-hydroxyisovaleric acid (Hiv). Additionally, it includes Dhoya residue (2,2-dimethyl-3-hydroxy-7-octynoic acid) with a terminal alkyne, as does yanucamide. To explore the potential therapeutic applications of **1**, we carried out its total synthesis, which involves a total of 20 chemical steps (longest linear sequence 12 steps) in a convergent process, solving the supply issue. The synthetic product and the natural product produced identical spectroscopic data. The in vitro cytotoxic activity against four human tumor cell lines, as well as the PD-1/PD-L1 inhibition were evaluated. Unfortunately, no significant bioactivity was found in any of the assays for this compound.

## 2. Results and Discussions

### 2.1. Isolation and Structure Elucidation of PM170453 (***1***)

**Figure 1:**
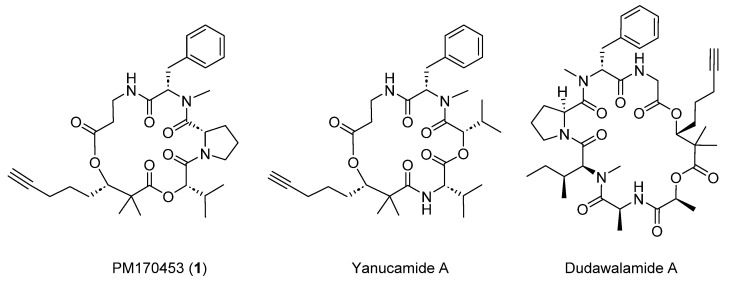
Chemical structures of PM170453 (**1**), yanucamide A and dudawalamide A.

During our screening program to find new chemical entities from marine organisms that reverse immune suppression by inhibiting PD-1 receptor activity, the crude extract of a marine *Lyngbya* sp. collected off the coast of Buru, Indonesia showed hints of inhibition of this interaction (45% inhibition compared with untreated control cells). Bioassay-guided fractionation of an organic extract of the organism, including VLC RP-18 chromatography followed by reverse-phase preparative HPLC of selected fractions, led to the isolation of compound **1** (Figure 1).

Compound **1** was isolated as an optically active white powder with a pseudomolecular ion in the (+)-HRESITOFMS *m*/*z* 596.3364 [M + H]^+^. The presence of 31 signals on the ^13^C NMR spectrum (Table 1) was also in agreement with the molecular formula C_33_H_45_N_3_O_7_ (*m*/*z* 596.3364 [M + H]^+^, calcd. for C_33_H_46_N_3_O_7_ *m*/*z* 596.3330 [M + H]^+^). The peptide nature of **1** was evident from its ^1^H and ^13^C NMR spectra (Figures S1–S9). ^1^H NMR data in CD_3_OD (Table 1) displayed the characteristic α-amino or α-hydroxy acids at 5.18, 4.99, 4.60 and 4.55 ppm, corresponding to four methine carbons adjacent to heteroatoms at 78.4, 64.2, 78.4 and 57.3 ppm, respectively in the HSQC experiment. This assignation is supported by the presence of five carbonyl groups in the range 178.2–169.5 ppm. A signal at 8.57 ppm in the ^1^H NMR when performed in CD_3_OH was attributable to a NH amide. Combination of ^13^C NMR and HSQC spectra showed a N-Me group at 29.9 ppm. Additionally, an aromatic ring with five aromatic methines in the range 139.4–127.9 ppm as well as a terminal acetylene group, presumed by the presence of a characteristic methine carbon at 70.1 (observed when the HSQC experiment is performed with a *J*_H-C_ = 250 Hz) together with the quaternary carbon at 84.4 ppm, were part of this molecule. 

**Figure 2:**
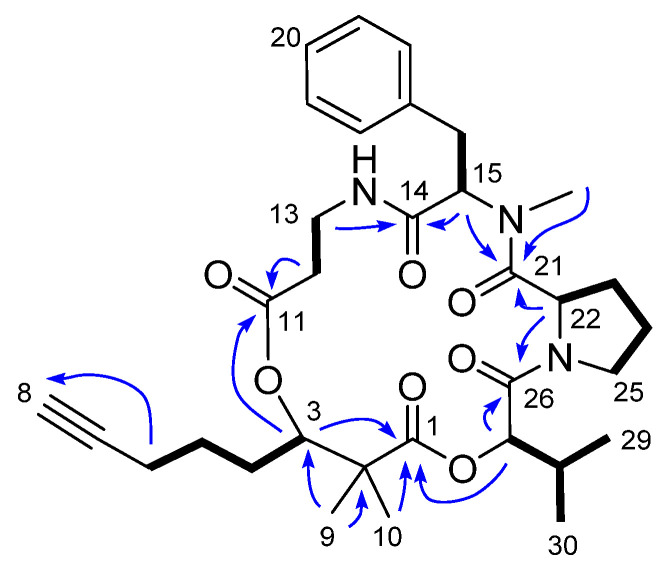
COSY (bold) and selected HMBC (blue) correlations for compound **1**.

Extensive 2D NMR analysis of COSY, HSQC and HMBC (Figure 2) revealed the presence of Dhoya residue and β-alanine (β-Ala), *N*-methylphenylalanine (*N*Me-Phe), proline (Pro) and 2-hydroxyvaleric acid (Hiv) units. The spin system corresponding to 2,2-dimethyl-3-hydroxy-7-octanoic acid was determined by the COSY correlation from position H-3 (5.18 ppm) to H-6 (2.22 ppm) and the long range cross-peaks between the acetylene H-8 (2.24 ppm) to the methylene C-6 (18.7 ppm), H-6 (2.22 ppm) to C-7 and C-8 (84.4 and 70.1 ppm, respectively) and the methyl singlets H-9 (1.31 ppm) and H-10 (1.16 ppm) to C-1, C-2 and C-3 (178.2, 47.7 and 78.4 ppm, respectively). In the same way, the spin systems corresponding to β-Ala and Pro were deduced from their COSY correlations between adjacent methylenes H-12 and H-13 (2.88/2.65 and 3.67/3.18 ppm) and from positions H-22 to H-25 (4.55, 1.02/0.87, 1.84/1.70, 3.75/3.44 ppm), respectively. Furthermore, the presence of *N*Me-Phe and Hiv was inferred from the COSY correlations between H-15 and H-16 (4.99 and 3.30/2.98 ppm) and from H-27 to H-30 (4.60, 2.11, 1.05, 0.99 ppm) correspondingly. Finally, the connectivity between these moieties was deduced by the HMBC correlations observed from the relationship of the α-protons and *N*Me to the adjacent carbonyls, establishing the sequence of **1** as Dhoya/β-Ala/*N*Me-Phe/Pro/Hiv.

Due to the small amount of the compound isolated, we could not carry out further experiments to determine the absolute configuration of the chiral centres. Nonetheless, we assumed the same configuration of the residues as those of the known yanucamide due to their spectroscopic similarities.

### 2.2. Total Synthesis of ***1***

**Figure 3:**
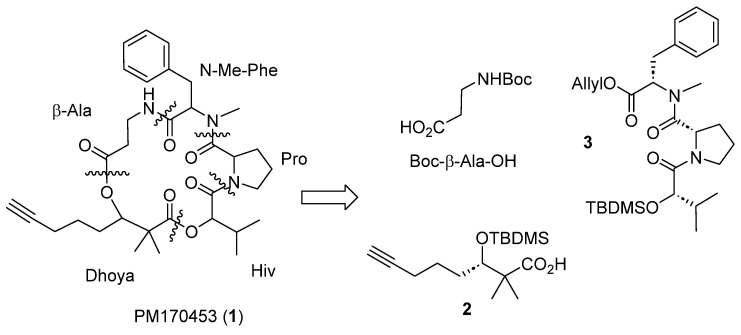
Retrosynthetic analysis of PM170453 (**1**).

To solve the supply problem for further development and to confirm the proposed stereochemistry, we completed the first total synthesis of **1**. The synthetic methodology involves a linear sequence of 12 chemical steps using (*S*)-2-hydroxyisovaleric acid (Hiv) as starting material. Key elements of the approach include different coupling and esterification reactions using appropriately protected amino acids and other fragments and a final cyclization between β-alanine and *N*-methyl-phenylalanine to create **1** (Figure 3). This ring-closure is sufficiently unhindered to allow for smooth cyclization and was achieved without any racemization. This procedure allowed us to obtain a few grams of compound **1**, from key fragments **2** and **3**.

**Figure 4:**
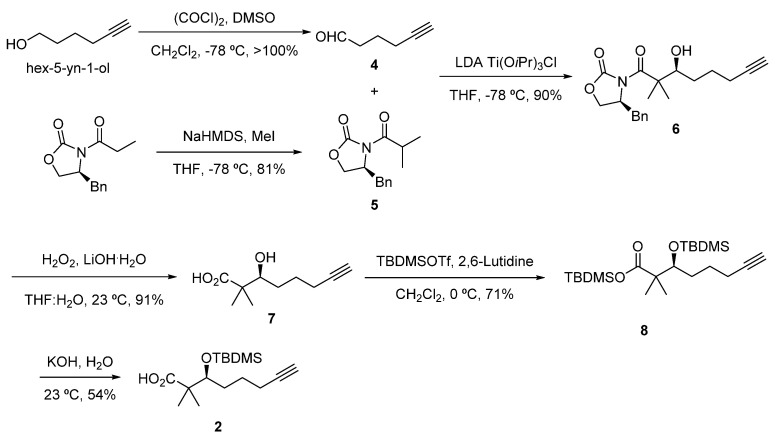
Preparation of fragment **2**.

The Dhoya fragment **2** (Figure 4) was constructed in five linear steps from Hex-5-yn-1-ol. Swern oxidation gave aldehyde **4,** which was converted into the (*S*)-α-hydroxy acid **7** via an asymmetric aldol reaction with oxazolidinone **5**, previously obtained by methylation of (*S*)-4-benzyl-3-propionyl-2-oxazolidinone and cleavage of Evans oxazolidinone **6** with LiOH/H_2_O_2_ [10]. Double silyl protection of **7** using TBDMS-triflate resulted in intermediate **8,** which was subjected to saponification, affording the corresponding (*S*)-3-((*tert*-butyldimethylsilyl)oxy)-2,2-dimethyloct-7-ynoic acid fragment **2** after acidification.

**Figure 5:**
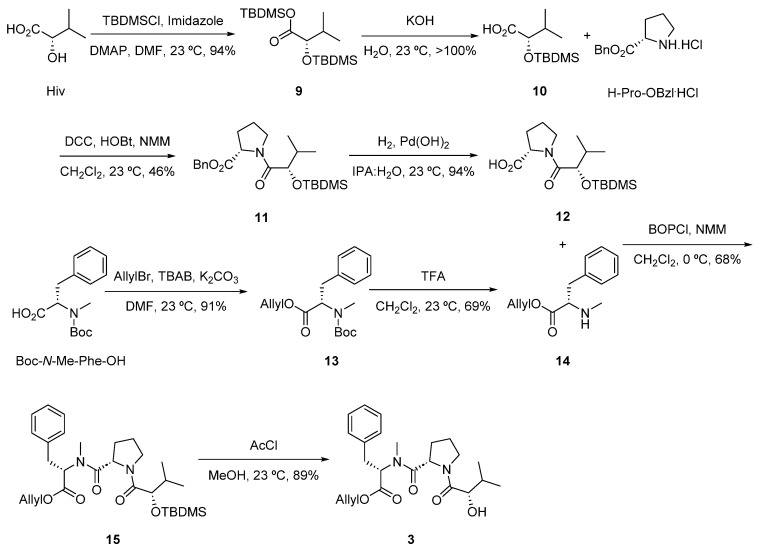
Preparation of fragment **3**.

Fragment **3** was a straightforward preparation starting from (*S*)-2-hydroxyisovaleric acid (Hiv) (Figure 5). Hiv was converted to silyl ether intermediate **10** following the same strategy as that used to prepare fragment **2** from intermediate **7** [11]. The coupling of **10** with *L*-proline benzyl ester hydrochloride, mediated by DCC, NMM and HOBt, proceeded smoothly to give **11** with a 46% yield. Hydrogenolytic removal of the benzyl group in **11** was achieved by using Pd(OH)_2_ in a 2:1 mixture of IPA:H_2_O to obtain acid **12**. At the same time, Boc-*N*-methyl-*L*-phenylalanine was protected as the allyl ester, and acidic Boc removal gave *N*-methyl intermediate **14**. Coupling reaction between **12** and **14** using BOPCl and NMM afforded amide **15** with a 68% yield. Deprotection of the TBDMS silyl group using acetyl chloride in MeOH gave fragment **3** in excellent yield (89%).

**Figure 6:**
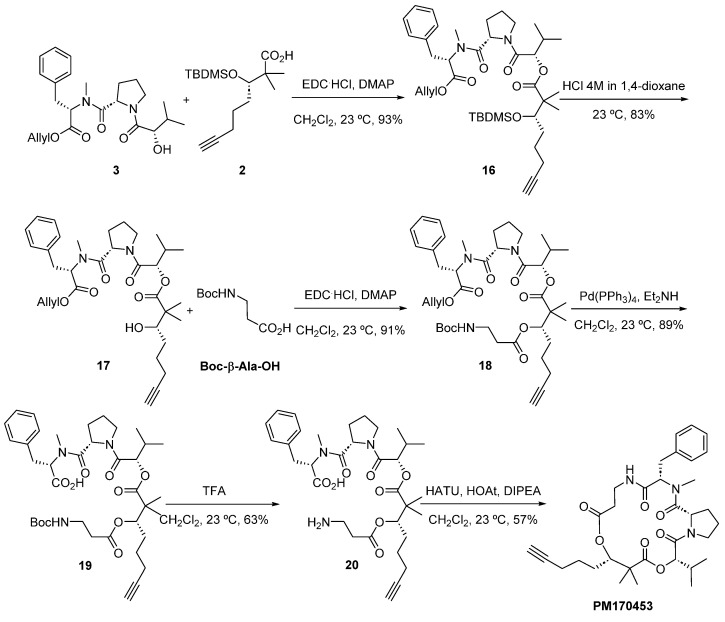
Preparation of PM170453 (**1**).

The construction of **1** from the three fragments was completed using standard procedures (Figure 6). Esterification between fragments **2** and **3** was mediated by the use of EDC and DMAP to give intermediate **16** at a 93% yield. Removal of the silyl protecting group in acidic media afforded alcohol **17**, and esterification under the same conditions with Boc-β-Ala-OH produced precursor **18** at a 91% yield. Consecutive unmasking of the carboxylic acid and the primary amine then allowed for cyclization via activation with HATU and HOAt, at high dilution in CH_2_Cl_2_ (57% yield). In this way, we completed the total synthesis of **1** with all the chiral centers C-3, C-15, C-22 and C-27 in an *S* configuration and unambiguously confirmed the structural assignment of the natural product. All the spectral data (^1^H and ^13^C NMR; Figures S13–S51), HPLC retention times and biological activities of the synthetic sample exactly matched those of the isolated natural product **1**.

### 2.3. Biological Activities

The in vitro cytotoxic activity of the new cyclic depsipeptide **1** was tested against four human tumor cell lines, lung (A-549), colon (HT-29), breast (MDA-MB-231) and pancreas (PSN-1), following a published procedure [12]. Doxorubicin was used as the positive control (Figure S11). Table 2 provides the data on the biological activity of **1** (GI_50_ value). 

In addition, the PD-1/PD-L1 inhibition was also tested. The PD-1 assay measures receptor activation through co-culture of cells expressing PD-1 with PD-L1 presenting cells. BMS202 was used as control for PD-1/PD-L1 interaction inhibition. A PrestoBlue cell proliferation assay was simultaneously performed to control the cytotoxicity of the samples. The ability of PM170453 to inhibit PD-1/PD-L1 interaction was also determined using the well-established HTRF assay. Although we detected the same slight activity of **1** as in the crude extract, it was not significant enough to be considered (Figure S12, Table 3). 

## 3. Materials and Methods

### 3.1. General Experimental Procedures

Dry solvents were purchased and used without any extra processing. All reagents were used as purchased without further purification unless otherwise stated. All reactions, unless otherwise indicated, were performed under an atmosphere of nitrogen in oven-dried glassware. Routine monitoring of reactions was performed using silica gel TLC plates (Merck 60 F254). Spots were visualized by UV and/or dipping the TLC plate into an ethanolic phosphomolybdic acid solution and heating with a hot plate. Flash chromatography was carried out on silica gel 60 (200–400 mesh). ^1^H and ^13^C NMR spectra were recorded on a Varian Unity 400 or 500 spectrometer at 400 or 500 MHz and 100 or 125 MHz, respectively. Chemical shifts (δ) are reported in parts per millions (ppm) referenced to CHCl_3_ at 7.26 ppm for ^1^H and CDCl_3_ at 77.0 ppm for ^13^C and to CH_3_OH at 3.31 ppm for ^1^H and CD_3_OD at 49.0 ppm. Coupling constants are reported in hertz (Hz), with the following abbreviations used: s = singlet, d = doublet, t = triplet, q = quartet, m = multiplet. When appropriate, the multiplicities are preceded with br, indicating that the signal was broad. High-resolution mass spectroscopy (HRMS) was performed using an Agilent 6230 TOF LC/MS system and the ESI-MS technique. (+)-ESIMS were recorded using an Agilent 1100 Series LC/MSD spectrometer. The PathHunter^®^ U2OS PD-L1/PD-L1 Dimerization Cell Line system was used to screen compounds that block PD-1/PD-L1 to enhance T cell response and mediate antitumor activity. Optical rotations were determined using a Jasco P-1020 polarimeter with a sodium lamp and are reported as follows: [α]_D_: (*c* g/100 mL, solvent).

### 3.2. Biological Material

A Cyanophycea, belonging to the Family *Oscillatoriacea* Engler (1898), was collected by hand using a Rebreather diving system in Buru, Indonesia (3°50.439’ S/ 127°08.706’ E) at depths ranging between 6 and 63 m. This biological material was identified as *Lyngbya* sp. A sample of the specimen was deposited in PharmaMar, Spain, with the reference code BURU-169.

### 3.3. Extraction and Isolation of ***1***

A frozen specimen of the sample described in Section 3.2. (42 g) was diced and extracted at room temperature with magnetic stirring, using a mixture of 50:50 CH_2_Cl_2_:CH_3_OH (3 × 250 mL). The organic extracts were evaporated to provide a dry residue of 1.5 g. The organic extract was subjected to step gradient VLC on Lichroprep RP-18 eluting initially with H_2_O to CH_3_OH and subsequently from CH_3_OH to CH_2_Cl_2_. The fraction eluted with CH_3_OH (55.0 mg) was subjected to semipreparative reversed phase HPLC (SymmetryPrep C18, 7 μm, 7.8 × 150 mm, gradient H_2_O:CH_3_CN from 60 to 80% in 20 min, UV detection, flow 2.3 mL/min) to afford compound **1** (0.8 mg, retention time 8.5 min).

Compound **1**: White powder. (+)-HRESITOFMS *m*/*z* 596.3364 [M + H]^+^ (calcd for C_33_H_46_N_3_O_7_ *m*/*z* 596.3330); ^1^H (500 MHz) and ^13^C NMR (125 MHz) see Table 1. [α]_D_: −32.4 (*c* 0.073, MeOH).

### 3.4. Total Synthesis of ***1***

#### 3.4.1. Preparation of Fragment **2** ((*S*)-3-((Tert-butyldimethylsilyl)oxy)-2,2-dimethyloct-7-ynoic Acid)

Compound **4**: A solution of oxalyl chloride (1.9 mL, 22.67 mmol) in CH_2_Cl_2_ (36 mL) was cooled to −78 °C and stirred for 30 min at this temperature. A 10% solution of DMSO (1.6 mL, 22.67 mmol) in CH_2_Cl_2_ (16 mL) was added dropwise at −78 °C. Once the gas emission was finished, the reaction mixture was stirred for 15 min. After that, 5-hexyn-1-ol (0.89 g, 9.07 mmol) was added and the mixture was stirred for 45 min. Finally, triethylamine (6.3 mL, 45.34 mmol) was added, and the mixture was stirred for 20 min. The reaction mixture was allowed to warm to 23 °C and washed with a saturated aqueous solution of NH_4_Cl and a saturated aqueous solution of NaCl. The combined organic layers were dried over anhydrous Na_2_SO_4_ and filtered, and the solvent was concentrated using a temperature of 40 °C and a pressure of 600 mbar. Et_2_O was added several times and CH_2_Cl_2_ removed to give a solution of **4** (1.62 g, >100% yield), which was used “as is” in the next step. ^1^H-NMR (400 MHz, CDCl_3_): δ 9.80–9.62 (m, 1H), 2.61–2.44 (m, 2H), 2.25–2.09 (m, 2H), 1.99–1.85 (m, 1H), 1.85–1.70 (m, 2H). ^13^C-NMR (100 MHz, CDCl_3_): δ 201.5, 83.1, 69.3, 42.4, 20.8, 17.7.

Compound **5**: A solution of NaHDMS (47.2 mL, 1.0 M in THF, 47.2 mmol) in THF (64.3 mL) was added to a solution of (*S*)-(+)-4-benzyl-3-propionyl-2-oxazolidinone (10.0 g, 42.87 mmol) in THF (21.4 mL) at −78 °C. Once the addition was finished, the reaction mixture was stirred for 1 h at −78 °C. Methyl iodide (5.3 mL, 85.74 mmol) was then added dropwise and the reaction stirred for 4 h at −78 °C, followed by TLC (8:2 Hex/EtOAc). After that, the cold-bath was removed and a saturated aqueous solution of NH_4_Cl was added. The solution was acidified with H_2_SO_4_ to pH 2. The layers were separated, and the aqueous layer extracted with EtOAc (3×). The organic extracts were washed with a saturated aqueous solution of NaHCO_3_, a saturated aqueous solution of Na_2_S_2_O_3_ and a saturated aqueous solution of NaCl. The combined organic layers were then dried over anhydrous Na_2_SO_4_ and filtered, and the solvent was removed under reduced pressure. The crude product obtained was purified using an automatic system for flash chromatography (SiO_2_, Hex:EtOAc mixtures from 100:0 to 30:70) to afford **5** (8.56 g, 81% yield). ^1^H-NMR (400 MHz, CDCl_3_): δ 7.39–7.16 (m, 5H), 4.68 (ddt, *J* = 10.6, 7.1, 3.2 Hz, 1H), 4.26–4.13 (m, 2H), 3.76 (dq, *J* = 13.3, 6.7 Hz, 1H), 3.27 (dd, *J* = 13.4, 3.2 Hz, 1H), 2.77 (dd, *J* = 13.3, 9.6 Hz, 1H), 1.22 (dd, *J* = 18.8, 6.8 Hz, 6H). ^13^C-NMR (100 MHz, CDCl_3_): δ 177.6, 153.0, 135.3, 129.4, 128.9, 127.3, 55.3, 37.9, 32.6, 19.2, 18.7. (+)ESIMS: *m*/*z* 248.1 [M + H]^+^, 270.2 [M + Na]^+^.

Compound **6**: A solution of diisopropylamine (12.2 mL, 86.76 mmol) in THF (434 mL) was added to *n*-BuLi (50 mL, 1.6 M in hexanes, 80.0 mmol) at −78 °C. The solution was warmed to 0 °C for 15 min and then cooled to −78 °C. A solution of **5** (13.4 g, 54.2 mmol) in THF (542 mL), previously cooled to −78 °C, was added dropwise to the LDA solution at −78 °C. After 30 min, chlorotriisopropoxytitanium (IV) (216 mL, 1 M in THF, 216 mmol) was added dropwise and the reaction mixture then warmed to −40 °C. After 1 h, the solution was cooled to −78 °C and **4** (15.6 g, 162.7 mmol) in THF (542 mL) was added dropwise at −78 °C and the solution warmed to −40 °C. After 3 h the reaction mixture was quenched with a saturated aqueous solution of NH_4_Cl, stirred in the presence of Celite^®^ until reaching 23 °C and filtered. The filtrate was extracted with EtOAc (4×), washed with a saturated aqueous solution of NaCl and the combined organic layers dried over anhydrous Na_2_SO_4_, and filtered, and the solvent was removed under reduced pressure. The crude product obtained was purified using an automatic system for flash chromatography (SiO_2_, Hex:EtOAc mixtures from 100:0 to 30:70) to give **6** (16.68 g, 90% yield). ^1^H-NMR (400 MHz, CDCl_3_): δ 7.38–7.18 (m, 5H), 4.75–4.65 (m, 1H), 4.24–4.05 (m, 3H), 3.27 (dd, *J* = 13.3, 3.3 Hz, 1H), 2.76 (dd, *J* = 13.3, 9.8 Hz, 1H), 2.26 (td, *J* = 6.8, 2.6 Hz, 2H), 1.94 (t, *J* = 2.6 Hz, 1H), 1.90–1.77 (m, 1H), 1.70–1.56 (m, 2H), 1.53–1.43 (m, 1H), 1.41 (s, 3H), 1.36 (s, 3H). ^13^C-NMR (100 MHz, CDCl_3_): δ 178.0, 152.3, 135.5, 129.4, 128.9, 127.3, 84.3, 75.0, 68.6, 66.3, 57.7, 50.1, 37.8, 30.2, 25.6, 20.2, 18.6, 18.2. (+)ESIMS: *m*/*z* 366.2 [M + Na]^+^.

Compound **7**: A hydrogen peroxide solution (19.2 mL, 187.66 mmol, 30%) and then lithium hydroxide monohydrate (3.15 g, 75.06 mmol) in H_2_O (94 mL) were added successively to a solution of **6** (16.1 g, 46.91 mmol) in a 4:1 mixture THF:H_2_O (338 mL:84 mL) at 0 °C. After stirring 2 h at 0 °C, Na_2_S_2_O_3_ was added. The solvent was removed under vacuum, and the residual aqueous layer was partitioned between CH_2_Cl_2_ (3×) and H_2_O. The combined aqueous layers were acidified to pH 1 with 1 N HCl. The aqueous layer was extracted with Et_2_O (3×), dried over anhydrous MgSO_4_, filtered and concentrated under reduced pressure to afford crude **7** (7.9 g, 91% yield), which was used in the next step without further purification. ^1^H-NMR (400 MHz, CDCl_3_): δ 3.67 (d, *J* = 11.9 Hz, 1H), 2.24 (td, *J* = 6.8, 2.7 Hz, 2H), 1.96–1.93 (m, 1H), 1.86–1.76 (m, 1H), 1.72–1.52 (m, 2H), 1.48–1.35 (m, 1H), 1.23 (s, 3H), 1.19 (s, 3H). ^13^C-NMR (100 MHz, CDCl_3_): δ 182.7, 76.0, 68.8, 68.7, 47.0, 30.3, 25.3, 22.6, 19.9, 18.1.

Compound **8**: A solution of **7** (6.1 g, 33.13 mmol) in CH_2_Cl_2_ (133 mL) was added to 2,6-lutidine (19.3 mL, 165.66 mmol) and *tert*-butyldimethylsilyltrifluoromethanesulphonate (30.4 mL, 132.53 mmol) at −78 °C. The reaction mixture was warmed at 0 °C and stirred 1 h. A saturated aqueous solution of NaHCO_3_ was then added, and the layers were separated. The aqueous layer was extracted with CH_2_Cl_2_ (3×). The combined organic layers were washed with a saturated aqueous solution of NaCl, dried over anhydrous Na_2_SO_4_, filtered and concentrated under reduced pressure. The crude product obtained was purified using an automatic system for flash chromatography (SiO_2_, Hex:EtOAc mixtures from 100:0 to 80:20) to give pure **8** (9.74 g, 71% yield). ^1^H-NMR (400 MHz, CDCl_3_): δ 3.86 (t, *J* = 4.8 Hz, 1H), 2.18–2.11 (m, 2H), 1.91 (td, *J* = 2.6, 1.4 Hz, 1H), 1.75–1.61 (m, 1H), 1.55–1.38 (m, 3H), 1.19–1.14 (m, 3H), 1.07 (d, *J* = 0.8 Hz, 3H), 0.94 (s, 9H), 0.89 (s, 9H), 0.26 (d, *J* = 1.1 Hz, 3H), 0.25 (d, *J* = 1.1 Hz, 3H), 0.08 (d, *J* = 0.9 Hz, 3H), 0.06 (d, *J* = 0.9 Hz, 3H). ^13^C-NMR (100 MHz, CDCl_3_): δ 177.6, 84.0, 76.4, 68.5, 49.3, 33.8, 26.0, 25.7, 25.5, 24.2, 18.7, 18.4, −3.0, −3.7, −4.0, −5.0. (+)ESIMS: *m*/*z* 413.3 [M + H]^+^, 435.4 [M + Na]^+^.

Fragment **2**: Compound **8** (9.7 g, 23.5 mmol) was cooled to 0 °C, and a solution of KOH (2.0 g, 35.79 mmol) in H_2_O (23.5 mL) was added dropwise. The reaction mixture was allowed to reach 23 °C, stirred for 30 min and diluted with hexane and H_2_O. The layers were separated and the aqueous layer extracted with Hexane (2×). The aqueous layer was acidified to pH 1–2 with HCl 1M. *Tert*-butyl methyl ether was then added and the aqueous layer extracted with further *tert*-butyl methyl ether (3×). The combined organic layers were dried over anhydrous Na_2_SO_4_, filtered and concentrated under reduced pressure. The crude product obtained was purified using an automatic system for flash chromatography (SiO_2_) to give pure **2** (3.81 g, 54% yield). ^1^H-NMR (400 MHz, CD_3_OD): δ 3.96 (dd, *J* = 6.8, 3.4 Hz, 1H), 2.23–2.09 (m, 3H), 1.76–1.42 (m, 4H), 1.15 (s, 3H), 1.08 (s, 3H), 0.90 (s, 9H), 0.10 (s, 3H), 0.07 (s, 3H). ^13^C-NMR (100 MHz, CD_3_OD): δ 179.6, 83.2, 76.8, 68.3, 32.6, 26.0, 25.2, 20.4, 19.7, 17.8, −5.0, −5.4. (+)ESIMS: *m*/*z* 299 [M + H]^+^, 305 [M + Li]^+^, 321 [M + Na]^+^.

#### 3.4.2. Preparation of Fragment **3** (Allyl N-(((*S*)-2-Hydroxy-3-methylbutanoyl)-L-prolyl)-N-methyl-L-phenylalaninate)

Compound **9**: A solution of (*S*)-2-hydroxy-3-methylbutanoic acid (23.5 g, 198.6 mmol) in DMF (149 mL) was added to imidazole (32.73 g, 480.7 mmol) and 4-dimethylaminopyridine (4.85 g, 39.7 mmol) at 0 °C. *Tert*-butyldimethylsilyl chloride (70.36 g, 466.8 mmol) was added in portions, controlling the temperature below 10 °C. Once the addition was finished, the reaction mixture was stirred for 15 min at 0 °C and then at 23 °C overnight. The reaction mixture was cooled to 0 °C and HCl 0.1 M was slowly added. The aqueous layer was extracted with *tert*-butyl methyl ether (3×), and the combined organic layers were washed with a saturated aqueous solution of NaHCO_3_ (2×) and a saturated aqueous solution of NaCl (2×). The combined organic layers were dried over anhydrous Na_2_SO_4_ and filtered, and the solvent was removed under reduced pressure to give crude **9** (64.54 g, 94% yield), which was used in the next step without further purification. ^1^H-NMR (400 MHz, CDCl_3_): δ 3.91 (d, *J* = 4.6 Hz, 1H), 2.11–1.93 (m, 1H), 0.94 (s, 9H), 0.91 (s, 9H), 0.27 (d, *J* = 3.7 Hz, 6H), 0.06 (s, 6H), 0.03 (s, 6H). ^13^C-NMR (100 MHz, CDCl_3_): δ 173.7, 77.5, 32.7, 25.7, 25.4, 19.1, 16.8, −5.0.

Compound **10**: Compound **9** (64.54 g, 186.2 mmol) was dissolved in a cooled solution of KOH (15.88 g, 283.6 mmol) in H_2_O (186 mL) at 0 °C, keeping the temperature at about 7.5 °C. When the addition was finished, the reaction mixture was stirred for 2 h at 23 °C and was then extracted with hexane (3×). The aqueous layer was cooled at 0 °C, acidified with 2M HCl to pH = 1–2 and extracted with *tert*-butylmethyl ether (3×). The combined organic layers were washed with a saturated aqueous solution of NaCl, dried over anhydrous Na_2_SO_4_, filtered and concentrated to give crude **10** (48 g, >100% yield), which was used in the next step without further purification. ^1^H-NMR (400 MHz, CDCl_3_): δ 4.10 (d, *J* = 3.6 Hz, 1H), 2.16–2.03 (m, 1H), 1.20 (s, 6H), 0.96 (s, 9H), 0.13 (d, *J* = 5.8 Hz, 6H). ^13^C-NMR (100 MHz, CDCl_3_): δ 175.9, 76.8, 32.9, 25.8, 19.1, 18.6, 16.8, −4.8.

Compound **11**: A solution of L-proline benzyl ester hydrochloride (3.0 g, 12.41 mmol) in CH_2_Cl_2_ (37.2 mL) was added to 4-methylmorpholine (4.1 mL, 37.23 mmol) at 0 °C. The reaction mixture was stirred for 10 min at 0 °C and a solution of **10** (3.2 g, 12.91 mmol) in CH_2_Cl_2_ (32.3 mL) was added at 0 °C. Then *N*, *N*′-dicyclohexylcarbodiimide (2.6 g, 12.41 mmol) and 1-hydroxybenzotriazole (2.0 g, 14.89 mmol) were added at 0 °C. The reaction mixture was allowed to reach 23 °C and stirred for 3 h. The precipitate was filtered and washed with EtOAc. The filtrate was concentrated under reduced pressure, and the residue obtained was dissolved in Et_2_O. The solution was filtrated, and the solid was washed with Et_2_O and EtOAc. The filtrate was washed with citric acid, a saturated aqueous solution of NaHCO_3_ and a saturated aqueous solution of NaCl. The combined organic layers were dried over anhydrous Na_2_SO_4_, filtered and concentrated under reduced pressure. The crude product obtained was purified using an automatic system for flash chromatography (SiO_2_, Hex:EtOAc mixtures from 100:0 to 70:30) to give pure **11** (2.5 g, 46% yield). ^1^H-NMR (400 MHz, CDCl_3_): δ 7.45–7.29 (m, 5H), 5.15 (s, 2H), 4.59–4.51 (m, 1H), 3.97 (d, *J* = 7.1 Hz, 1H), 3.81–3.63 (m, 2H), 2.24–2.07 (m, 1H), 2.05–1.95 (m, 2H), 1.94–1.83 (m, 2H), 0.96 (d, *J* = 6.8 Hz, 3H), 0.95–0.88 (m, 12H), 0.07 (s, 3H), 0.05 (s, 3H). ^13^C-NMR (100 MHz, CDCl_3_): δ 172.0, 171.6, 135.8, 128.6, 128.4, 128.1, 78.8, 66.6, 59.4, 32.3, 28.5, 25.8, 25.7, 25.5, 18.8, 18.3, 18.3, −4.7, −5.4. (+)ESIMS: *m*/*z* 420.4 [M + H]^+^, 442.3 [M + Na]^+^, 861.5 [2M + Na]^+^.

Compound **12**: A solution of **11** (2.3 g, 5.48 mmol) and Pd(OH)_2_ (1.15 g, 50% wt) in a 2:1 mixture of *i*-PrOH:H_2_O (120 mL:60 mL) was stirred for 3 h at 23 °C under an H_2_ atmosphere. The reaction mixture was filtered through Celite^®^ and washed with *i*-PrOH, and the solvent was concentrated under reduced pressure to yield crude **12** (1.7 g, 94% yield), which was used in the next step without further purification. ^1^H-NMR (400 MHz, CD_3_OD): δ 4.37 (dd, *J* = 8.6, 4.8 Hz, 1H), 4.09 (d, *J* = 6.9 Hz, 1H), 3.71 (t, *J* = 6.1 Hz, 2H), 2.27–2.12 (m, 1H), 2.11–1.82 (m, 4H), 0.98 (dd, *J* = 6.7 Hz, 3H), 0.97 (d, *J* = 6.8 Hz, 3H), 0.92 (s, 9H), 0.07 (s, 6H). ^13^C-NMR (100 MHz, CD_3_OD): δ 172.5, 77.1, 60.2, 32.2, 28.5, 25.0, 24.9, 24.8, 23.8, 17.8, 17.7, 17.1, −5.9, −6.4. (+)ESIMS: *m*/*z* 330.2 [M + H]^+^.

Compound **13**: A solution of Boc-*N*-Methyl-*L*-Phenylalanine (5 g, 17.9 mmol) in DMF (537 mL) was sequentially added to K_2_CO_3_ (9.89 g, 71.6 mmol), tetrabutylammonium bromide (0.69 g, 2.15 mmol) and allyl bromide (4.65 mL, 53.70 mmol) at 23 °C before being stirred for 1 h. The reaction mixture was then diluted with H_2_O and EtOAc, and the layers were separated. The aqueous layer was extracted with EtOAc (3×); the combined organic layers were washed with a saturated aqueous solution of NaCl, dried over anhydrous Na_2_SO_4_, filtered and concentrated. The crude product obtained was purified by flash chromatography on silica gel (Hex:EtOAc from 100:0 to 70:30) to yield **13** (5.21 g, 91% yield). ^1^H-NMR (400 MHz, CDCl_3_): δ 7.28–7.13 (m, 6H), 6.00–5.80 (m, 1H), 5.40–5.18 (m, 2H), 4.71–4.53 (m, 3H), 3.39–3.23 (m, 1H), 3.12–2.95 (m, 1H), 2.74 (s, 3H), 1.33 (s, 9H). ^13^C-NMR (100 MHz, CDCl_3_): δ 173.0, 157.9, 156.9*, 140.4, 140.2*, 134.8, 134.6*, 131.6, 131.5*, 130.9, 130.8*, 129.0, 128.9*, 120.5, 120.2*, 82.0, 81.8*, 67.8, 67.7*, 64.2, 62.5*, 38.0, 37.4*, 35.2, 34.8, 30.6, 30.5*. *indicates rotamer. (+)ESIMS: *m*/*z* 342.2 [M + Na]^+^.

Compound **14**: A solution of **13** (5.10 g, 16 mmol) in CH_2_Cl_2_ (84.6 mL) was added to TFA (35.1 mL) at 23 °C. The reaction mixture was stirred at 23 °C for 45 min. The mixture was concentrated in vacuum by co-evaporating with toluene (3×). A saturated aqueous solution of NaHCO_3_ was added to the residue obtained, which was then extracted with CH_2_Cl_2_ (3×), dried over anhydrous Na_2_SO_4_, filtered and concentrated to afford crude **14** (2.42 g, yield 69%), which was used in the next step without further purification. ^1^H-NMR (400 MHz, CDCl_3_): δ 7.33–7.13 (m, 5H), 5.82 (ddt, *J* = 16.3, 10.5, 5.9 Hz, 1H), 5.31–5.16 (m, 2H), 4.60–4.51 (m, 2H), 3.48 (t, *J* = 6.9 Hz, 1H), 2.97 (d, *J* = 6.8 Hz, 2H), 2.38 (s, 3H). ^13^C-NMR (100 MHz, CDCl_3_): δ 173.9, 137.0, 131.8, 129.2, 128.4, 126.7, 118.6, 65.3, 64.6, 39.4, 34.7. (+)ESIMS: *m*/*z* 242.2 [M + Na]^+^.

Compound **15**: A solution of **12** (1 g, 3.03 mmol) in CH_2_Cl_2_ (3 mL) was added to bis(2-oxo-3-oxazolidinyl)phosphinic chloride (0.81 g, 3.19 mmol) and 4-methylmorpholine (367 μL, 3.33 mmol) at 0 °C. The reaction mixture was cooled to −15 °C and stirred for 30 min. Then a solution of **14** (0.66 g, 3.03 mmol) in CH_2_Cl_2_ (6 mL) was added at −15 °C, followed by additional 4-methylmorpholine (1.1 mL, 10.01 mmol). The reaction mixture was stirred for 15 min at −15 °C, warmed to 0 °C and stirred overnight. EtOAc was added, and the organic layer was washed with 10% HCl, a 5% aqueous solution of NaHCO_3_ and a saturated aqueous solution of NaCl. The combined organic layers were dried over anhydrous Na_2_SO_4_, filtered and concentrated under reduced pressure. The crude product obtained was purified using an automatic system for flash chromatography (SiO_2_, Hex:EtOAc mixtures from 100:0 to 60:40) to give pure **15** (1.1 g, 68% yield). ^1^H-NMR (500 MHz, CDCl_3_): δ 7.33–7.27 (m, 2H), 7.25–7.17 (m, 3H), 5.97–5.81 (m, 1H), 5.37–5.12 (m, 3H), 4.88 (dd, *J* = 8.3, 4.0 Hz, 1H), 4.72–4.52 (m, 2H), 3.98 (d, *J* = 7.1 Hz, 1H), 3.83–3.56 (m, 2H), 3.32 (dd, *J* = 14.3, 6.6 Hz, 1H), 3.11–3.03 (m, 1H), 3.01 (s, 3H), 2.21–2.11 (m, 1H), 2.05–1.82 (m, 4H), 0.98–0.86 (m, 15H), 0.08 (s, 3H), 0.06 (s, 3H). ^13^C-NMR (100 MHz, CDCl_3_): δ 172.6*, 172.5, 171.1, 170.6*, 170.3, 137.4, 136.8*, 131.9, 131.5*, 129.3*, 129.0, 128.7*, 128.6*, 128.5*, 128.4, 126.8*, 126.5, 118.9*, 118.3, 78.3, 66.0*, 65.7, 60.3, 59.4, 58.7*, 56.5, 55.7*, 47.1*, 47.0, 34.7, 34.2*, 33.3, 32.4, 31.9*, 27.9, 25.9, 25.8, 25.7, 25.2, 21.0*, 20.3*, 18.8, 18.3 (×2), 17.9*, 17.8*, 14.2, −4.6, −5.0*, −5.3, −5.6*.* indicates rotamers. (+)ESIMS: *m*/*z* 531.5 [M + H]^+^, 553.4 [M + Na]^+^, 1083.5 [2M + Na]^+^.

Fragment **3**: A solution of **15** (604 mg, 1.14 mmol) in MeOH (6.3 mL) was added to acetyl chloride (16 μL, 0.23 mmol) at 0 °C. The reaction mixture was stirred for 6 h at 23 °C and concentrated under reduced pressure. The crude product obtained was purified using an automatic system for flash chromatography (SiO_2_, Hex:EtOAc mixtures from 100:0 to 20:80) to give pure **3** (423 mg, 89% yield). ^1^H-NMR (500 MHz, CDCl_3_): δ 7.36–7.26 (m, 3H), 7.25–7.14 (m, 2H), 5.95–5.81 (m, 1H), 5.31–5.20 (m, 2H), 5.10–5.04 (m, 1H), 4.83–4.89 (m, 1H), 4.75–4.64 (m, 1H), 4.58 (qdt, *J* = 13.1, 5.7, 1.3 Hz, 2H), 4.10 (d, *J* = 2.9 Hz, 1H), 3.53–3.67 (m, 3H), 3.36 (dd, *J* = 14.6, 5.8 Hz, 1H), 3.11 (dd, *J* = 14.6, 9.8 Hz, 1H), 2.96 (s, 3H), 2.19–2.10 (m, 2H), 2.08–1.99 (m, 2H), 1.98–1.83 (m, 4H), 1.07 (d, *J* = 6.9 Hz, 3H), 0.81 (d, *J* = 6.7 Hz, 3H). ^13^C-NMR (100 MHz, CDCl_3_): δ 172.0, 170.3, 137.1, 131.8, 128.8, 128.5, 126.6, 118.5, 73.4, 65.8, 59.7, 56.7, 46.7, 34.3, 33.6, 31.0, 28.0, 25.0, 19.7, 14.9. (+)ESIMS: *m*/*z* 417.4 [M + H]^+^, 439.3 [M + Na]^+^, 855.3 [2M + Na]^+^.

#### 3.4.3. Preparation of **1**

Compound **16**: A solution of **2** (2.25 g, 5.40 mmol) in CH_2_Cl_2_ (54 mL) was sequentially added **3** (3.22 g, 10.80 mmol), EDC^.^HCl (10.36 g, 54.02 mmol,) and DMAP (6.6 g, 54.02 mmol) at 0 °C. The reaction mixture was stirred overnight at 23 °C and then cooled to 0 °C and diluted with H_2_O and CH_2_Cl_2_. The layers were separated, and the aqueous layer was extracted with CH_2_Cl_2_ (3×). The combined organic layers were washed with a saturated aqueous solution of NaCl, dried over anhydrous Na_2_SO_4_, filtered and concentrated under reduced pressure. The crude product obtained was purified using an automatic system for flash chromatography (SiO_2_, Hex:EtOAc mixtures from 100:0 to 30:70) to yield pure **16** (3.51 g, 93% yield). ^1^H-NMR (400 MHz, CDCl_3_): δ 7.31–7.13 (m, 5H), 5.92–5.79 (m, 1H), 5.29–5.15 (m, 1H), 5.10–5.01 (m, 1H), 4.88 (dd, *J* = 7.9, 3.9 Hz, 1H), 4.71 (d, *J* = 6.7 Hz, 1H), 4.58–4.51 (m, 1H), 3.93–3.79 (m, 2H), 3.70–3.60 (m, 1H), 3.31 (dd, *J* = 14.3, 6.4 Hz, 1H), 3.07 (dd, *J* = 14.4, 8.8 Hz, 1H), 2.95 (s, 3H), 2.26–2.05 (m, 6H), 2.03–1.94 (m, 1H), 1.91–1.87 (m, 1H), 1.86–1.79 (m, 1H), 1.74–1.57 (m, 2H), 1.57–1.39 (m, 3H), 1.19 (s, 3H), 1.13 (s, 3H), 1.06–0.97 (m, 6H), 0.89 (s, 9H), 0.09 (s, 3H), 0.05 (s, 3H). ^13^C-NMR (100 MHz, CDCl_3_): δ 176.8, 172.2, 170.3, 167.2, 137.2, 131.9, 128.8, 128.3, 126.4, 118.2, 84.2, 76.5, 76.1, 68.2, 65.6, 60.1, 59.7, 56.4, 53.4, 48.3, 46.8, 34.4, 33.7, 33.4, 30.0, 28.1, 26.1, 25.9, 24.8, 24.0, 20.8, 18.5, 18.2, 17.8, −3.7, −4.1. (+)ESIMS: *m*/*z* 697.4 [M + H]^+^, 719.3 [M + Na]^+^.

Compound **17**: A solution of HCl (37.65 mL, 4.0 M in 1,4-dioxane, 150.6 mmol) was added to **16** (3.5 g, 5.02 mmol) at 23 °C. The reaction mixture was stirred for 1 h at 23 °C and concentrated under reduced pressure. The crude product obtained was purified using an automatic system for flash chromatography (SiO_2_, Hex:EtOAc mixtures from 100:0 to 40:60) to obtain pure **17** (2.44 g, 83% yield). ^1^H-NMR (400 MHz, CDCl_3_): δ 7.33–7.11 (m, 5H), 5.96–5.80 (m, 1H), 5.27–5.17 (m, 2H), 5.15–5.06 (m, 1H), 4.89–4.80 (m, 2H), 4.65–4.48 (m, 2H), 3.80–3.68 (m, 2H), 3.67–3.57 (m, 2H), 3.50 (d, *J* = 10.2 Hz, 1H), 3.34 (dd, *J* = 14.9, 5.6 Hz, 1H), 3.08 (dd, *J* = 14.7, 10.0 Hz, 1H), 2.88 (s, 3H), 2.31–2.19 (m, 4H), 2.18–2.08 (m, 2H), 1.94 (bs, 1H), 1.89–1.79 (m, 1H), 1.69–1.56 (m, 2H), 1.55–1.43 (m, 1H), 1.24 (s, 3H), 1.14 (s, 3H), 1.07 (d, *J* = 6.8 Hz, 3H), 0.99 (d, *J* = 6.7 Hz, 3H). ^13^C-NMR (100 MHz, CDCl_3_): δ 178.1, 171.9, 170.5, 168.5, 136.8, 131.8, 128.7, 128.6, 126.7, 118.6, 84.6, 76.8, 76.2, 72.3, 71.1, 68.3, 65.9, 60.4, 59.1, 56.9, 47.8, 46.9, 34.2, 33.2, 29.3, 27.8, 25.7, 24.8, 22.3, 19.4, 18.3, 17.4, 16.7. (+)ESIMS: *m*/*z* 583.4 [M + H]^+^, 605.3 [M + Na]^+^.

Compound **18**: A solution of **17** (2.44 g, 4.19 mmol) in CH_2_Cl_2_ (42 mL) was sequentially added to Boc-β-alanine (3.97 g, 20.95 mmol), EDC^.^HCl (8.03 g, 41.90 mmol) and DMAP (5.12 g, 41.90 mmol) at 0 °C. The reaction mixture was stirred for 3 h at 23 °C and then cooled to 0 °C and diluted with H_2_O and CH_2_Cl_2_. The layers were separated, and the aqueous layer was extracted with CH_2_Cl_2_ (3×). The combined organic layers were washed with a saturated aqueous solution of NaCl, dried over anhydrous Na_2_SO_4_, filtered and concentrated under reduced pressure. The crude product obtained was purified using an automatic system for flash chromatography (SiO_2_, Hex:EtOAc mixtures from 100:0 to 30:70) to obtain pure **18** (2.86 g, 91% yield). ^1^H-NMR (400 MHz, CDCl_3_): δ 7.32–7.22 (m, 3H), 7.22–7.14 (m, 2H), 5.93–5.79 (m, 1H), 5.28–5.16 (m, 4H), 5.05 (dd, *J* = 9.3, 6.2 Hz, 1H), 4.88–4.83 (m, 1H), 4.70 (d, *J* = 6.0 Hz, 1H), 4.58–4.53 (m, 2H), 3.85–3.75 (m, 1H), 3.69–3.60 (m, 1H), 3.40–3.26 (m, 3H), 3.07 (dd, *J* = 14.5, 9.3 Hz, 1H), 2.92 (s, 3H), 2.57–2.49 (m, 2H), 2.28–2.17 (m, 4H), 2.16–2.07 (m, 2H), 1.94–1.92 (m, 1H), 1.88–1.78 (m, 1H), 1.76–1.55 (m, 4H), 1.42 (s, 9H), 1.21 (s, 3H), 1.20 (s, 3H), 1.03 (d, *J* = 11.1 Hz, 3H), 1.01 (d, *J* = 10.9 Hz, 3H). ^13^C-NMR (100 MHz, CDCl_3_): δ 157.2, 153.7, 153.3, 152.0, 151.9, 148.7, 118.6, 113.3, 110.3, 109.9, 108.0, 99.9, 65.4, 58.4, 50.1, 47.2, 41.8, 41.0, 38.0, 28.3, 28.0,17.7, 15.9, 14.8, 11.3, 10.5, 9.1, 9.5, 6.6, 6.2, 2.9, 2.4, 2.1, 0.1, −0.5, −0.9, −4.40. (+)ESIMS: *m*/*z* 654.3 [M + H-Boc]^+^, 754.4 [M + H]^+^, 776.3 [M + Na]^+^.

Compound **19**: A solution of **18** (2 g, 2.65 mmol) in CH_2_Cl_2_ (40 mL) was added to Pd(PPh_3_)_4_ (307 mg, 0.26 mmol) and diethylamine (1.4 mL, 13.27 mmol) at 0 °C. The reaction mixture was stirred for 15 min at 23 °C and then concentrated under reduced pressure. The crude product obtained was purified by flash chromatography on silica gel (Hex:EtOAc from 80:20 to 20:80) to obtain pure **19** (1.68 g, 89% yield). ^1^H-NMR (400 MHz, CDCl_3_): δ 7.34–7.27 (m, 3H), 7.14–7.09 (m, 2H), 5.23 (dd, *J* = 0.9, 3.3 Hz, 1H), 5.07–4.91 (m, 1H), 4.59 (d, *J* = 5.8 Hz, 1H), 4.26–4.16 (m, 1H), 3.79–3.66(m, 1H), 3.53–3.43 (m, 1H), 3.41–3.29 (m, 2H), 3.23 (dd, *J* = 14.5, 3.1 Hz, 1H), 3.04 (dd, *J* = 14.4, 11.7 Hz, 1H), 2.90 (s, 3H), 2.54 (t, *J* = 6.1 Hz, 2H), 2.29–2.17 (m, 2H), 2.18–2.08 (m, 1H), 2.06–1.96 (m, 1H), 1.96–1.90 (m, 1H), 1.74–1.59 (m, 4H), 1.55–1.45 (m, 2H), 1.43 (s, 9H), 1.29–1.21 (m, 2H), 1.19 (s, 3H), 1.18 (s, 3H), 0.97 (d, *J* = 11.2 Hz, 3H), 0.95 (d, *J* = 11.1, 3H). ^13^C-NMR (100 MHz, CD_3_OD): δ 175.6, 172.3, 171.17, 168.2, 138.4, 129.1, 128.5, 128.0, 125.8, 78.7, 76.8, 59.7, 57.1, 46.7, 46.4, 36.0, 34.4, 34.0, 31.5, 29.7, 28.9, 27.7, 27.3, 25.1, 24.4, 20.9, 20.7, 19.2, 17.8, 17.3, 16.8, 7.9. (+)ESIMS: *m*/*z* 614.5 [M + H − Boc]^+^, 714.4 [M + H]^+^, 736.3 [M + Na]^+^.

Compound **20**: A solution of **19** (1.5 g, 2.10 mmol) in CH_2_Cl_2_ (33.4 mL) was added to TFA (4.6 mL) at 23 °C. The reaction mixture was stirred for 45 min at 23 °C and then concentrated under reduced pressure. The crude product obtained was purified by flash chromatography on silica gel (CH_2_Cl_2_:CH_3_OH from 100:0 to 0:100) to yield pure **20** (812 mg, 63% yield). ^1^H-NMR (400 MHz, CD_3_OD): δ 7.36–7.15 (m, 5H), 5.30–5.21 (m, 1H), 4.96–4.74 (m, 5H), 3.90–3.74 (m, 1H), 3.70–3.58 (m, 1H), 3.25–3.09 (m, 4H), 3.00–2.93 (m, 1H), 2.92 (s, 3H), 2.86–2.70 (m, 2H), 2.28–2.15 (m, 4H), 2.13–1.94 (m, 2H), 1.84–1.58 (m, 2H), 1.56–1.42 (m, 2H), 1.23 (s, 3H), 1.19 (s, 3H), 1.08 (d, *J* = 6.8 Hz, 3H), 1.02 (d, *J* = 6.7 Hz, 3H). ^13^C-NMR (100 MHz, CD_3_OD): δ 175.6, 172.5, 172.2, 170.6, 168.2, 137.6, 129.2, 128.6, 128.5, 128.2, 126.3, 83.2, 77.5, 76.8, 68.7, 60.3, 57.2, 46.4, 35.0, 33.8, 30.8, 29.6, 28.8, 27.6, 25.0, 24.4, 20.2, 19.8, 17.9, 17.3, 16.6. (+)ESIMS: *m*/*z* 614.3 [M + H]^+^.

Compound **1**: A solution of **20** (771 mg, 1.26 mmol) in CH_2_Cl_2_ (931 mL) was sequentially added to HATU (1.1 g, 2.89 mmol), DIPEA (0.98 mL, 5.66 mmol) and HOAt (4.8 mL, 2.89 mmol) at 0 °C. The reaction mixture was stirred overnight at 23 °C and washed with a saturated aqueous solution of NH_4_Cl and a saturated aqueous solution of NaCl. The combined organic layers were dried over anhydrous Na_2_SO_4_, filtered and concentrated under reduced pressure. The crude product obtained was purified by flash chromatography on silica gel and then by semipreparative reversed phase HPLC (SymmetryPrep C18, gradient H_2_O:CH_3_CN from 60 to 80% in 20 min, UV detection, flow 1 mL/min) to give pure **1** (430 mg, 57% yield). Synthetic PM170453 exhibited physical, spectroscopic (^1^H, ^13^C NMR and MS) and biological characteristics identical to those obtained for the natural product. [α]_D_: −38.2 (c 0.07, CH_2_Cl_2_).

### 3.5. Biological Activity

#### 3.5.1. Bioassay for the Detection of Antitumor Activity

The aim of this assay is to evaluate the in vitro cytostatic (ability to delay or arrest tumor cell growth) or cytotoxic (ability to kill tumor cells) activity of the samples being tested. A colorimetric assay, using a sulforhodamine B (SRB) reaction, has been adapted to provide a quantitative measurement of cell growth and viability [12,13]. This form of assay employs 96-well cell culture microplates. All the cell lines used in this study were obtained from the American Type Culture Collection (ATCC), unless otherwise indicated, and derived from different types of human cancer: A-549 (ATCC CCL-185), lung carcinoma; HT-29 (ATCC HTB-38), colorectal carcinoma; MDA-MB-231 (ATCC HTB-26), breast adenocarcinoma; and PSN-1, pancreatic adenocarcinoma [14]. Cell lines were maintained in Dulbecco’s Modified Eagle Medium (DMEM) (for A-549, HT-29 and MDA-MB-231) or RPMI (for PSN-1) supplemented with 10% Fetal Bovine Serum (FBS), 2 mM *L-*glutamine, 100 U/mL penicillin and 100 U/mL streptomycin, at 37 °C, 5% CO_2_ and 98% humidity. For the experiments, cells were harvested from subconfluent cultures using trypsinization and resuspended in fresh medium before counting and plating.

Cells were seeded in 96-well microtiter plates at 5 × 10^3^ cells per well in aliquots of 150 μL and allowed to attach to the plate surface for 18 h (overnight) in drug-free medium. After that, one control (untreated) plate of each cell line was fixed (as described below) and used for time zero reference value. Culture plates were then treated with test compounds (50 μL aliquots of 4× concentrated compound stock solutions made in complete culture medium) using ten serial dilutions (concentrations ranging from 10 to 0.000262 μg/mL) and triplicate cultures (final concentration of DMSO being 1%). After 72 h treatment, the antitumor effect was measured using the SRB methodology: briefly, cells were washed twice with PBS, fixed for 15 min in 1% glutaraldehyde solution at room temperature, rinsed twice in PBS and stained in 0.4% SRB solution for 30 min at room temperature. Cells were then rinsed several times with 1% acetic acid solution and air-dried at room temperature. SRB was then extracted in 10 mM trizma base solution and the absorbance measured in an automated spectrophotometric plate reader at 490 nm. Effects on cell growth and survival were estimated by applying the NCI algorithm [15]. Doxorubicin and DMSO (solvent) were used as the positive and negative controls, respectively, in this assay. Prism 9.1.0. from GraphPad was used for the statistical analysis of the cell growth inhibition results. Using the mean ± SD of triplicates, a dose–response curve was automatically generated using nonlinear regression analysis to a 4-parameter logistic curve. Three reference parameters were calculated (NCI algorithm) by automatic interpolation: GI_50_ = compound concentration that produces 50% cell growth inhibition, as compared to control cultures; TGI = total cell growth inhibition (cytostatic effect), as compared to control cultures and LC_50_ = compound concentration that produces 50% net cell killing (cytotoxic effect).

#### 3.5.2. PathHunter PD-1 Assay

A PD-1 signalling assay was used for quantifying SHP phosphatase recruitment to PD-1, using enzyme fragment complementation (EFC) technology. PD-1 harbours immunoreceptor tyrosine inhibitory motifs (ITIMs) in its cytoplasmic tail. When its ligand (PL-L1) binds to PD-1, the ITIM motif is phosphorylated, resulting in the recruitment of SHP-1 and SHP-2 (SH2-domain containing phosphatases), inhibiting the T-cell response. Full-length PD-1 receptor was engineered with a small β-gal fragment fused to its C-terminus, and the SH2-domain of SHP-1 was engineered with the complementing β-gal fragment. These constructs were stably expressed in Jurkat cells, while untagged full-length PD-L1 was stably expressed in U-2 OS cells (ligand-presenting cells). Ligand engagement, through co-culture with ligand-presenting cells, results in phosphorylation of PD-1 fusion protein, leading to the recruitment of SHP-1 which forces complementation of the EFC components to create an active β-gal enzyme. This active enzyme hydrolyzes substrate to create chemiluminescence as a measure of receptor activity.

The assay was performed following the manufacturer’s instructions (Eurofins DiscoverX, Fremont, CA, USA). Briefly, 8 × 10^3^ PathHunter^®^ Jurkat PD-1 (SHP1) signaling cells (DiscoverX, Cat. 93-1104C19) were seeded into a 384-microwell assay plate with assay medium, followed by addition of the crude extract (75 μg/mL) or serial dilutions (ranging from 100 to 0.05 μM) of **1** or serial dilutions of control BMS202 (Selleckchem, Cologne, Germany) (ranging from 24 to 0.0012 μM). The cells were incubated at 37 °C in 5% CO_2_ incubator for 1 h to allow for compounds action. Subsequently, 12 × 10^3^ PathHunter^®^ U2OS PD-L1 Ligand Cell Line signalling cells (DiscoverX, Cat. No 93-1066C3) were then added to each well with treated PathHunter^®^ Jurkat PD-1 (SHP1) cells and incubated for 2 h at room temperature. The assay plate was processed using the PathHunter Bioassay Detection Kit (DiscoverX, 93-0933), and the detection of the assay chemiluminescence signal was evaluated on a PerkinElmer Envision reader. Data were plotted using GraphPad Prism 9.1.0.; EC_50_ values were calculated using a sigmoidal dose–response curve fit with variable slope (four parameter) with no constraints.

A PrestoBlue cell proliferation assay was performed following the manufacturer’s instructions (ThermoFisher scientific, Waltham, MA USA). Cellular viability was estimated from conversion of resazurin to its colored reaction product, resorufin. Absorbance was measured at 570 nm in a Perkin-Elmer EnVision reader. Cell survival was expressed as percentage of control cell growth.

#### 3.5.3. Homogeneous Time-Resolved Fluorescence (HTRF) Binding Assay

The assay kit of PD-1/PD-L1 interaction (64ICP01PEG) was purchased from Cisbio (Shanghai, China). Binding assays were performed according to the manufacturer’s instructions. The binding of Tag2-PD-1 and Tag1-PD-L1 was detected by anti-Tag1-EuK (HTRF donor) and anti-Tag2-XL665 (HTRF acceptor). Compounds blocking PD-1/PD-L1 complex formation reduce the HTRF signal. The inhibitory percentage was calculated following the instructions of the assay kit. Briefly, Tag2-PD-1 (25 nM final) and 1/3 serial dilutions PM170453 (ranging from 100 to 0.05 μM final), or 1/3 serial dilutions BMS-936559 (ranging from 4 to 0.0002 μM final), were added one after the other to a 384-well plate and incubated for 1 h at room temperature (RT). Then, Tag1-PD-L1 (5 nM final) was added for a total volume of 10 μL. After 1 h of incubation, detection reagent (10 μL) containing 1.83 nM anti-Tag1-EuK and 66.7 nM anti-Tag2-XL665 was added into the assay well. The signals were measured by a microplate reader (Perkin-Elmer EnVision) with the excitation wavelength located at 320 nm and the emission wavelength of 665 and 620 nm, respectively. The HTRF ratio = (665 nm/620 nm) was obtained on the microplate reader.

## 4. Conclusions

In this study, we present the isolation and structural elucidation of a new cyclic depsipeptide, PM170453 (**1**) from a marine cyanobacterial strain *Lyngbya* sp. collected in Buru, Indonesia. The structure of **1** was determined by spectroscopic methods including HRMS and extensive 1D and 2D NMR. To explore its pharmacological potential and biological applications, the total synthesis of **1** comprising a total of 20 chemical steps in a convergent process has been carried out, which confirmed the stereochemistry proposed. Although initial indications suggested inhibition of PD1-PDL1 interactions by a crude extract of the original marine sample, the pure compound failed to show significant cytotoxicity against four human tumor cell lines and also failed to impair PD1-PDL1 interaction. These findings underscore the complexity of natural product discovery and emphasize the need for comprehensive screening approaches to identify bioactive molecules effectively.

## Figures and Tables

**Table 1:** ^1^H (500 MHz) and ^13^C (125 MHz) NMR data of **1** in CD_3_OD.

Unit		δ_C_, Type	δ_H_, (*J* in Hz)
**Dhoya**	**1**	178.2, C	-
	**2**	47.7, C	-
	**3**	78.4, CH	5.18, dd (10.7, 2.3)
	**4**	29.0, CH_2_	1.79, m; 1.59, m
	**5**	26.0, CH_2_	1.48, m; 1.43, m
	**6**	18.7, CH_2_	2.22, m
	**7**	84.4, C	-
	**8**	70.1, CH	2.24, s
	**9**	17.2, CH_3_	1.31, s
	**10**	24.5, CH_3_	1.16, s
**β** **-Ala**	**11**	173.0, C	-
	**12**	32.2, CH_2_	2.88, ddd (18.6, 13.0, 2.7); 2.65, ddd (18.6, 4.7, 2.0)
	**13**	36.7, CH_2_	3.67, m; 3.18, m
	**NH**	-	8.57, brs ^a^
***N*Me-Phe**	**14**	171.6, C	-
	**15**	64.2, CH	4.99, dd (11.6, 3.3)
	**16**	34.6, CH_2_	3.30, m; 2.98, dd (14.7, 11.3)
	**17**	139.4, C	-
	**18**	130.5, CH	7.22, m
	**19**	129.8, CH	7.31, m
	**20**	127.9, CH	7.23, m
	*N*Me	29.9, CH_3_	2.84, s
**Pro**	**21**	174.9, C	-
	**22**	57.3, CH	4.55, dd (7.8, 7.8)
	**23**	29.5, CH_2_	1.02, m; 0.87, m
	**24**	26.5, CH_2_	1.84, m; 1.70, m
	**25**	48.7, CH_2_ ^b^	3.75, ddd (10.8, 7.6, 3.7); 3.44, m
**Hiv**	**26**	169.5, C	-
	**27**	78.4, CH	4.60, d (5.6)
	**28**	31.0, CH	2.11, m
	**29**	19.0, CH_3_	1.05, d (6.9)
	**30**	17.7, CH_3_	0.99, d (6.7)

^a^ in CD_3_OH; ^b^ under solvent.

**Table 2:** Antiproliferative activity data (M) of Compound **1**.

GI_50_ (M)	Lung A-549	Colon HT-29	Breast MDA-MB-231	Pancreas PSN-1
PM170453 (**1**)	9.74 × 10^−6^	9.06 × 10^−6^	>1.68 × 10^−5^	>1.68 × 10^−5^
Doxorubicin	3.97 × 10^−7^	4.14 × 10^−7^	3.28 × 10^−7^	2.07 × 10^−7^

**Table 3:** PD-1/PD-L1 inhibition data (M) of **1**.

EC_50_ (M)	PD-1	PR Blue	HTRF
PM170453 (**1**)	1.2 × 10^−4^	>1.0 × 10^−4^	>2.0 × 10^−4^
BMS202	1.1 × 10^−6^	1.0 × 10^−5^	7.3 × 10^−8^

## Data Availability

The data are contained within the article or Supplementary Materials.

## Supplementary Materials

The following supporting information can be downloaded at: https://www.mdpi.com/article/10.3390/md22070303/s1, Figures S1–S9: NMR spectra of **1**; Figure S10: HRESITOFMS spectrum of **1**; Figures S11 and S12: Dose-response curves and PD-1/PDL-1 interaction curves for **1**; Figures S13–S50: NMR spectra of the synthesis intermediates of **1**; Figure S51: ^1^H NMR spectra of synthetic vs. natural **1**.
