# Supplementary material for: Isolation and Total Synthesis of PM170453, a New Cyclic Depsipeptide Isolated from Lyngbya sp
Source: Mar Drugs. 2024 Jun 28;22(7):303. doi: 10.3390/md22070303 (PMC11277655; doi:10.3390/md22070303)

Supplementary Material

Isolation and Total Synthesis of PM170453, a New Cyclic Depsipeptide Isolated from *Lyngbya* sp

Rogelio Fernández,* Marta Pérez, Alejandro Losada, Silvia Reboredo, Asier Gómez-San Juan, María Jesús Martín,* Andrés Francesch, Simon Munt and Carmen Cuevas

Research and Development, PharmaMar S.A., Avda. de los Reyes 1, Pol. In. La Mina, 28770 - Colmenar Viejo, Madrid, Spain.

*Corresponding autor: *rfernandez@pharmamar.com*; *mjmartin@pharmamar.com*

Table of contents

1. NMR Spectra of natural PM170453 (1)

- **Figure S1.** ^{1}H NMR spectrum of **1**, (500 MHz, CD_3OD)……………...…………………………...**5**
- **Figure S2.** ^{1}H NMR spectrum of **1**, (500 MHz, CD_3OH)……………...…………………………...**6**
- **Figure S3.** ^{13}C NMR spectrum of **1**, (CD_3OD)…………………...……………………………...**7**
- **Figure S4.** *g*-COSY spectrum of **1**. (CD_3OD)………………...……………………………………**8**
- **Figure S5.** *g*-HSQC spectrum of **1**. (CD_3OD)……………………...………………………………**9**
- **Figure S6.** *g*-HSQC (J = 250 Hz) spectrum of **1**. (CD_3OD)…………………...…………………**10**
- **Figure S7.** *g*-HMBC spectrum of **1**. (CD_3OD)……………………...…………………………**11**
- **Figure S8.** ROESY spectrum of **1**. (CD_3OD)……………………...…………………………...**12**
- **Figure S9.** ROESY spectrum of **1**. (CD_3OH)…………………...……………………………...**13**

2. MS spectrum of 1

- **Figure S10.** (+)-HRESITOFMS spectrum of **1**……………………………………………………...**14**

3. Dose-response curves for 1

- **Figure S11** Dose-response curves for **1** in a panel of 4 tumour cell lines…….…………………..**15**
- **Figure S12.** PD-1/PDL-1 interaction curves for **1**………………………………………………...**16**

4. Spectra of synthesis intermediates of 1

- **Figure S13.** ^{1}H NMR spectrum of hex-5-ynal (**4**) (400 MHz, $CDCl_3$)………………………..….**17**
- **Figure S14.** ^{13}C NMR spectrum of hex-5-ynal (**4**) (100 MHz, $CDCl_3$)…………………………..**18**
- **Figure S15.** ^{1}H NMR spectrum of (*S*)-4-benzyl-3-isobutyryloxazolidin-2-one (**5**) (400 MHz, $CDCl_3$)……………………………………………………………………………………..**19**
- **Figure S16.** ^{13}C NMR spectrum of (*S*)-4-benzyl-3-isobutyryloxazolidin-2-one (**5**) (100 MHz, $CDCl_3$)……………………………………………………………………………………..**20**
- **Figure S17.** ^{1}H NMR spectrum of (*S*)-4-benzyl-3-((S)-3-hydroxy-2,2-dimethyloct-7-ynoyl)oxazolidin-2-one (**6**) (400 MHz, $CDCl_3$)……………………………………………...……**21**
- **Figure S18.** ^{13}C NMR spectrum of (*S*)-4-benzyl-3-((S)-3-hydroxy-2,2-dimethyloct-7-ynoyl)oxazolidin-2-one (**6**) (100 MHz, $CDCl_3$)………………………………………………...**22**
- **Figure S19.** ^{1}H NMR spectrum of (*S*)-3-hydroxy-2,2-dimethyloct-7-ynoic acid (**7**) (400 MHz, $CDCl_3$)……………………………………………………………………………………..**23**
- **Figure S20.** ^{13}C NMR spectrum of (*S*)-3-hydroxy-2,2-dimethyloct-7-ynoic acid (**7**) (100 MHz, $CDCl_3$)……………………………………………………………………………………..**24**
- **Figure S21.** ^{1}H NMR spectrum of *tert*-butyldimethylsilyl (*S*)-3-((*tert*-butyldimethylsilyl)oxy)-2,2-dimethyloct-7-ynoate (**8**) (400 MHz, $CDCl_3$)……………………………………………………..**25**
- **Figure S22.** ^{13}C NMR spectrum of *tert*-butyldimethylsilyl (*S*)-3-((*tert*-butyldimethylsilyl)oxy)-2,2-dimethyloct-7-ynoate (**8**) (100 MHz, $CDCl_3$)……………………………………………....**26**
- **Figure S23.** ^{1}H NMR spectrum of (*S*)-3-((*tert*-butyldimethylsilyl)oxy)-2,2-dimethyloct-7-ynoic acid (**2**) (400 MHz, CD_3OD)……………………………………………………………………**27**
- **Figure S24.** ^{13}C NMR spectrum of (*S*)-3-((*tert*-butyldimethylsilyl)oxy)-2,2-dimethyloct-7-ynoic acid (**2**) (100 MHz, CD_3OD)……………………………………………………………………**28**
- **Figure S25.** ^{1}H NMR spectrum of *tert*-butyldimethylsilyl (*S*)-2-((*tert*-butyldimethylsilyl)oxy)-3-methylbutanoate (**9**) (400 MHz, $CDCl_3$)…………………………………………………………..**29**

- **Figure S26.** ^{13}C NMR spectrum of *tert*-butyldimethylsilyl (*S*)-2-((*tert*-butyldimethylsilyl)oxy)-3-methylbutanoate (**9**) (100 MHz, $CDCl_3$)…………………………………………………………..**30**
- **Figure S27.** ^{1}H NMR spectrum of (*S*)-2-((*tert*-butyldimethylsilyl)oxy)-3-methylbutanoic acid (**10**) (400 MHz, $CDCl_3$)……………………………………………………………………………...**31**
  Figure S28. ^{13}C NMR spectrum of (*S*)-2-((*tert*-butyldimethylsilyl)oxy)-3-methylbutanoic acid (**10**) (100 MHz, $CDCl_3$)…………………………………………………………………………**32**
- **Figure S29.** ^{1}H NMR spectrum of benzyl ((*S*)-2-((*tert*-butyldimethylsilyl)oxy)-3-methylbutanoyl)-L-prolinate (**11**) (400 MHz, $CDCl_3$)…………………………………………………………….**33**
- **Figure S30.** ^{13}C NMR spectrum of benzyl ((*S*)-2-((*tert*-butyldimethylsilyl)oxy)-3-methylbutanoyl)-L-prolinate (**11**) (100 MHz, $CDCl_3$)……………………………………….…**34**
- **Figure S31.** ^{1}H NMR spectrum of ((*S*)-2-((*tert*-butyldimethylsilyl)oxy)-3-methylbutanoyl)-L-proline (**12**) (400 MHz, CD_3OD)……………………………………………………………….**35**
- **Figure S32.** ^{13}C NMR spectrum of ((*S*)-2-((*tert*-butyldimethylsilyl)oxy)-3-methylbutanoyl)-L-proline (**12**) (100 MHz, CD_3OD)……………………………………………………………….**36**
- **Figure S33.** ^{1}H NMR spectrum of allyl *N*-(*tert*-butoxycarbonyl)-*N*-methyl-L-phenylalaninate (**13**) (400 MHz, $CDCl_3$)……………………………………………………………………………...**37**
- **Figure S34.** ^{13}C NMR spectrum of allyl *N*-(*tert*-butoxycarbonyl)-*N*-methyl-L-phenylalaninate (**13**) (100 MHz, $CDCl_3$)……………………………………………………………………………...**38**
- **Figure S35.** ^{1}H NMR spectrum of allyl methyl-L-phenylalaninate (**14**) (400 MHz, $CDCl_3$)…....**39**
- **Figure S36.** ^{13}C NMR spectrum of allyl methyl-L-phenylalaninate (**14**) (100 MHz, $CDCl_3$)……**40**
- **Figure S37.** ^{1}H NMR spectrum of allyl *N*-(((*S*)-2-((*tert*-butyldimethylsilyl)oxy)-3-methylbutanoyl)-L-prolyl)-*N*-methyl-L-phenylalaninate (**15**) (500 MHz, $CDCl_3$)……………….**41**
- **Figure S38.** ^{13}C NMR spectrum of allyl *N*-(((*S*)-2-((*tert*-butyldimethylsilyl)oxy)-3-methylbutanoyl)-L-prolyl)-*N*-methyl-L-phenylalaninate (**15**) (100 MHz, $CDCl_3$)……………….**42**
- **Figure S39.** ^{1}H NMR spectrum of allyl *N*-(((*S*)-2-hydroxy-3-methylbutanoyl)-L-prolyl)-*N*-methyl-L-phenylalaninate (**3**) (500 MHz, $CDCl_3$)…………………………………………………….….**43**
- **Figure S40.** ^{13}C NMR spectrum of allyl *N*-(((*S*)-2-hydroxy-3-methylbutanoyl)-L-prolyl)-*N*-methyl-L-phenylalaninate (**3**) (100 MHz, $CDCl_3$)……………………………………………...**44**
- **Figure S41.** ^{1}H NMR spectrum of (*S*)-1-((*S*)-2-(((*S*)-1-(allyloxy)-1-oxo-3-phenylpropan-2-yl)(methyl)carbamoyl)pyrrolidin-1-yl)-3-methyl-1-oxobutan-2-yl (*S*)-3-((tert-butyldimethylsilyl)oxy)-2,2-dimethyloct-7-ynoate (**16**) (400 MHz, $CDCl_3$)……………………...**45**
- **Figure S42.** ^{13}C NMR spectrum of (*S*)-1-((*S*)-2-(((*S*)-1-(allyloxy)-1-oxo-3-phenylpropan-2-yl)(methyl)carbamoyl)pyrrolidin-1-yl)-3-methyl-1-oxobutan-2-yl (*S*)-3-((tert-butyldimethylsilyl)oxy)-2,2-dimethyloct-7-ynoate (**16**) (100 MHz, $CDCl_3$)…………………...…**46**
- **Figure S43.** ^{1}H NMR spectrum of (*S*)-1-((*S*)-2-(((*S*)-1-(allyloxy)-1-oxo-3-phenylpropan-2-yl)(methyl)carbamoyl)pyrrolidin-1-yl)-3-methyl-1-oxobutan-2-yl (*S*)-3-hydroxy-2,2-dimethyloct-7-ynoate (**17**) (400 MHz, $CDCl_3$)………………………………………………………………**47**
- **Figure S44.** ^{13}C NMR spectrum of (*S*)-1-((*S*)-2-(((*S*)-1-(allyloxy)-1-oxo-3-phenylpropan-2-yl)(methyl)carbamoyl)pyrrolidin-1-yl)-3-methyl-1-oxobutan-2-yl (*S*)-3-hydroxy-2,2-dimethyloct-7-ynoate (**17**) (100 MHz, $CDCl_3$)……………………………………………………...…..**48**
- **Figure S45.** ^{1}H NMR spectrum of (*S*)-1-((*S*)-2-(((*S*)-1-(allyloxy)-1-oxo-3-phenylpropan-2-yl)(methyl)carbamoyl)pyrrolidin-1-yl)-3-methyl-1-oxobutan-2-yl (*S*)-3-((3-((*tert*-butoxycarbonyl)amino)propanoyl)oxy)-2,2-dimethyloct-7-ynoate (**18**) (400 MHz, $CDCl_3$)…..…**49**
- **Figure S46.** ^{13}C NMR spectrum of (*S*)-1-((*S*)-2-(((*S*)-1-(allyloxy)-1-oxo-3-phenylpropan-2-yl)(methyl)carbamoyl)pyrrolidin-1-yl)-3-methyl-1-oxobutan-2-yl (*S*)-3-((3-((*tert*-butoxycarbonyl)amino)propanoyl)oxy)-2,2-dimethyloct-7-ynoate (**18**) (100 MHz, $CDCl_3$)……..**50**
- **Figure S47.** ^{1}H NMR spectrum of *N*-(((10*S*,14*S*)-14-isopropyl-2,2,11,11-tetramethyl-4,8,12-trioxo-10-(pent-4-yn-1-yl)-3,9,13-trioxa-5-azapentadecan-15-oyl)-L-prolyl)-*N*-methyl-L-phenylalanine (**19**) (400 MHz, $CDCl_3$)…………………………………………………………**51**
- **Figure S48.** ^{13}C NMR spectrum of *N*-(((10*S*,14*S*)-14-isopropyl-2,2,11,11-tetramethyl-4,8,12-trioxo-10-(pent-4-yn-1-yl)-3,9,13-trioxa-5-azapentadecan-15-oyl)-L-prolyl)-*N*-methyl-L-phenylalanine (**19**) (100 MHz, CD_3OD)………………………………………………………..**52**
- **Figure S49.** ^{1}H NMR spectrum of *N*-(((*S*)-2-(((S)-3-((3-aminopropanoyl)oxy)-2,2-dimethyloct-7-ynoyl)oxy)-3-methylbutanoyl)-L-prolyl)-*N*-methyl-L-phenylalanine (**20**) (400 MHz, CD_3OD)…**53**

- **Figure S50.** ^{13}C NMR spectrum of *N*-(((*S*)-2-(((S)-3-((3-aminopropanoyl)oxy)-2,2-dimethyloct-7-ynoyl)oxy)-3-methylbutanoyl)-L-prolyl)-*N*-methyl-L-phenylalanine (**20**) (100 MHz, CD_3OD)...**54**

21. Spectra of synthetic vs natural PM170453

Figure S51. ^{1}H NMR spectra of synthetic *vs.* natural **PM170453**……………………………………………**55**

Figure S1. ^{1}H NMR spectrum of **PM170453**, (500 MHz, CD_3OD).

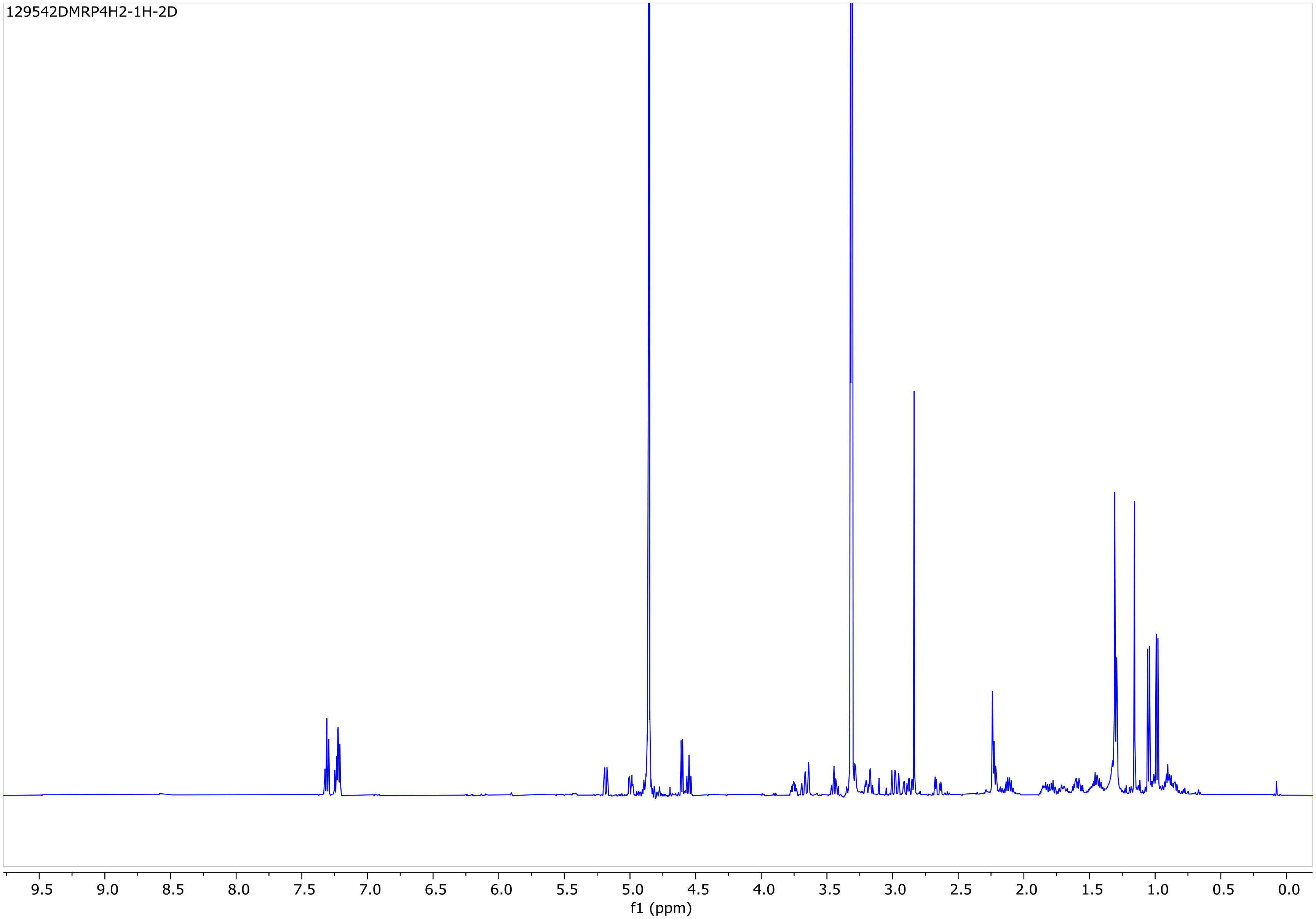

Figure S2. ^{1}H NMR spectrum of **PM170453**, (500 MHz, CD_3OH).

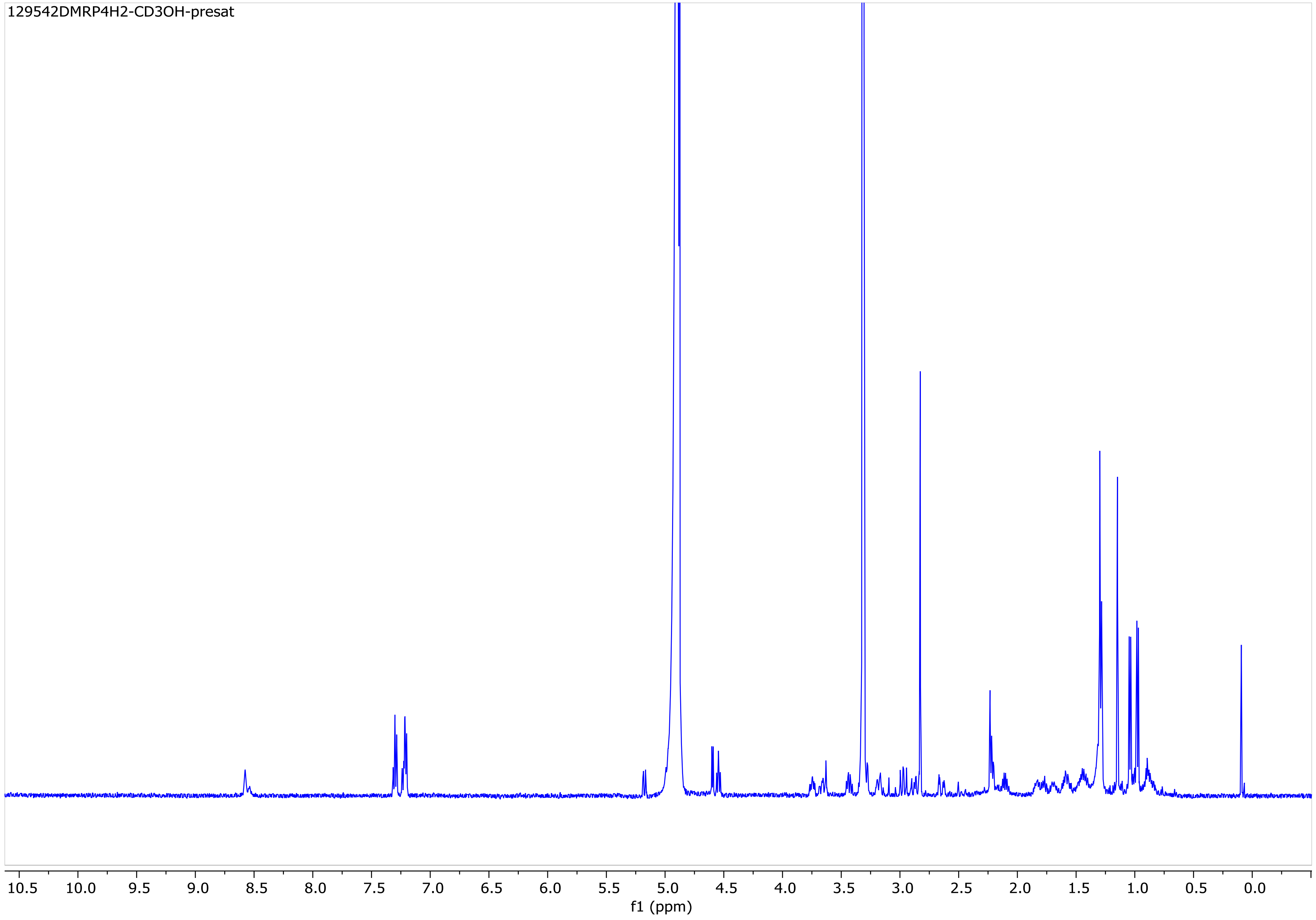

Figure S3. ^{13}C NMR spectrum of **PM170453**, (125 MHz, CD_3OD).

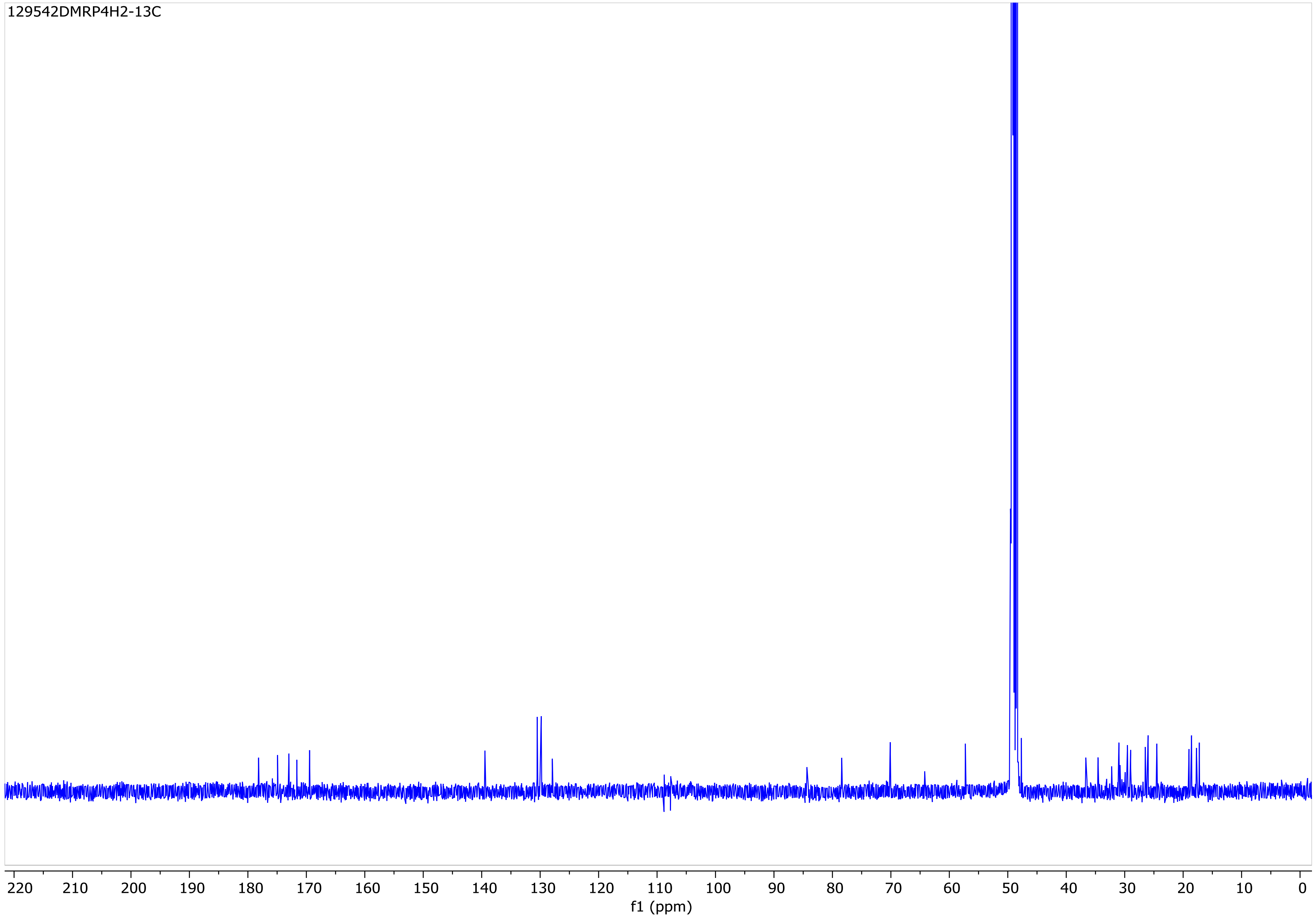

Figure S4. *g*-COSY spectrum of **PM170453**.

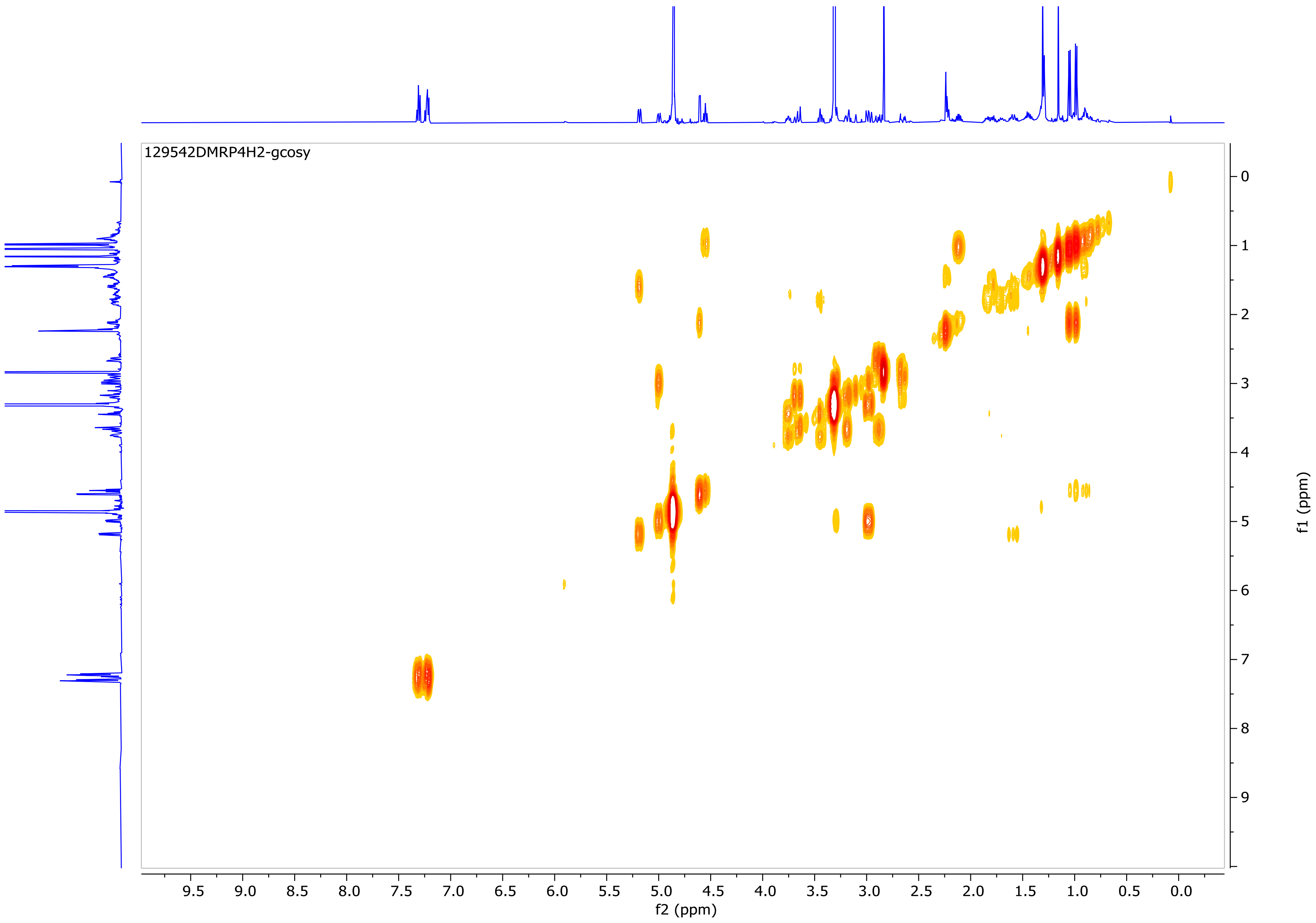

Figure S5. *g*-HSQC spectrum of **PM170453**

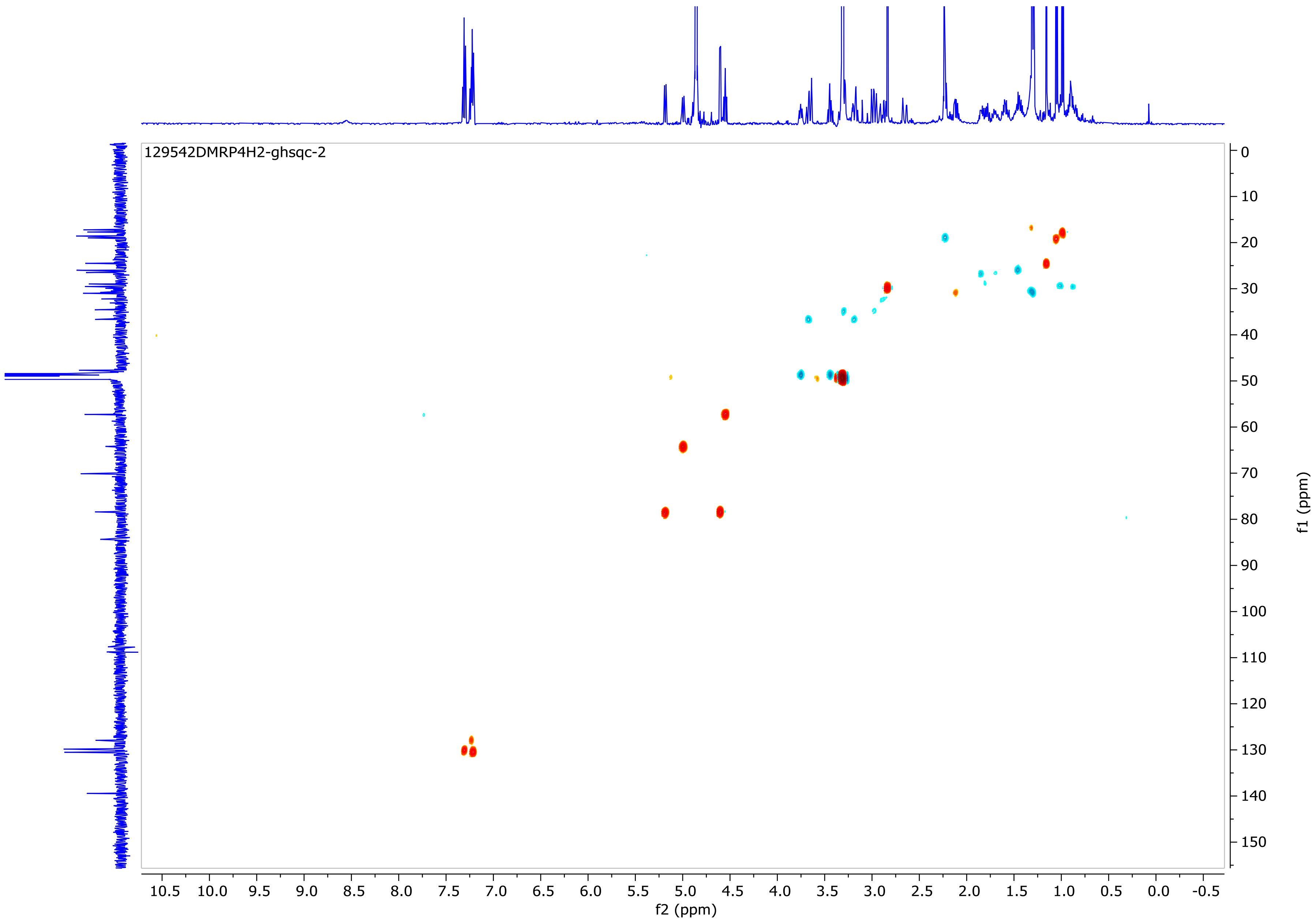

Figure S6. *g*-HSQC (J = 250 Hz) spectrum of **PM170453**.

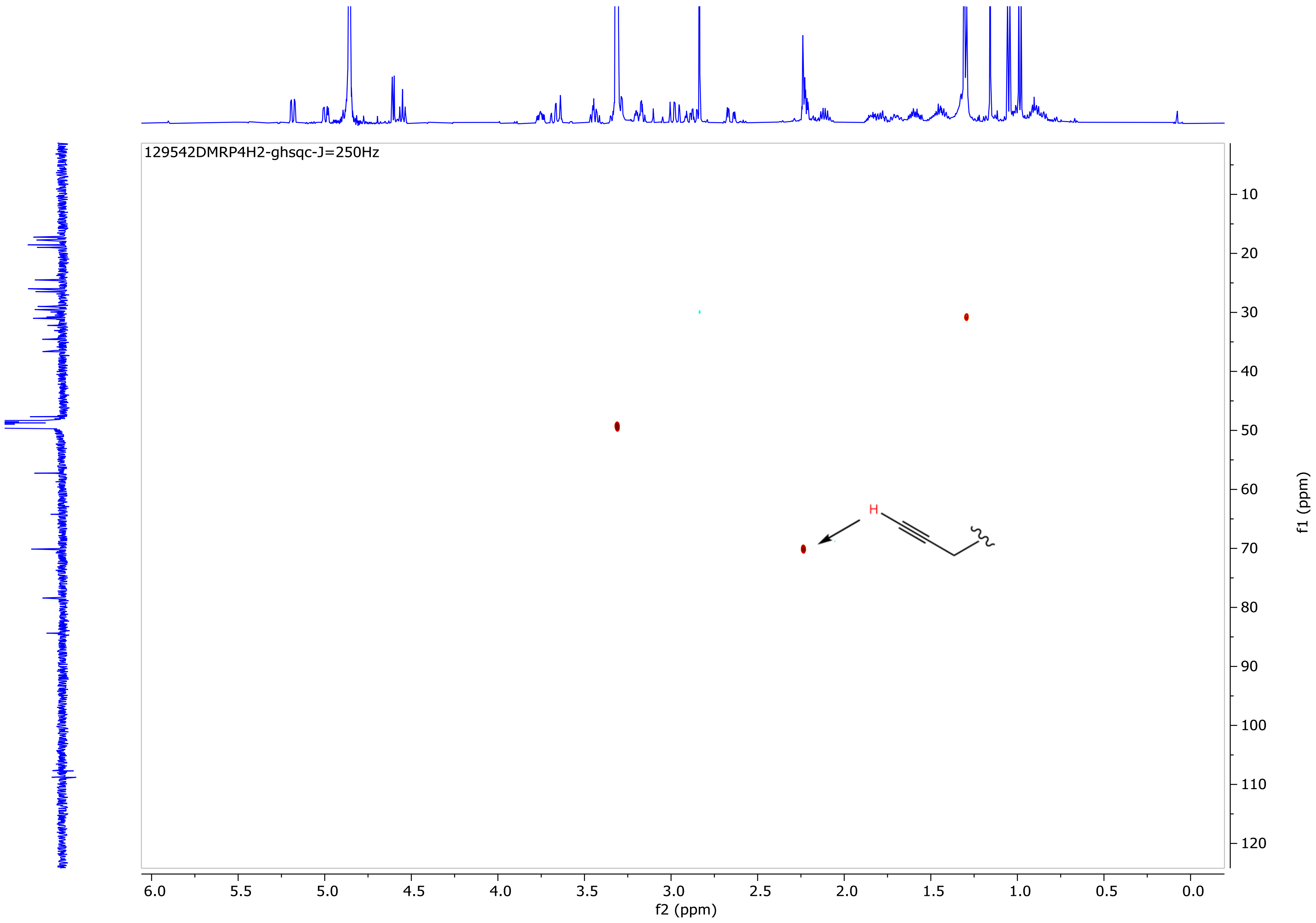

Figure S7. gHMBC spectrum of **PM170453**.

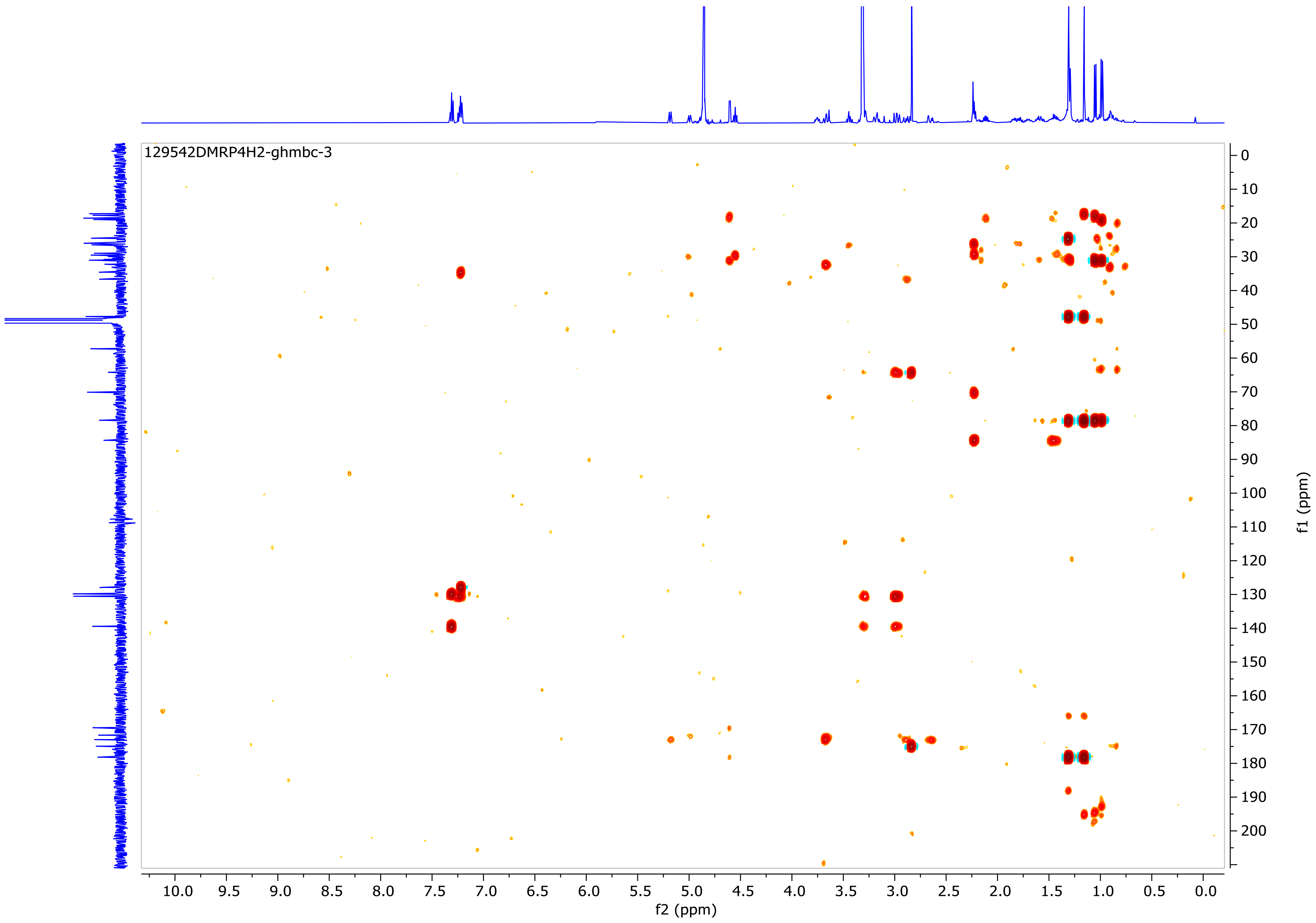

Figure S8. ROESY spectrum of **PM170453**.

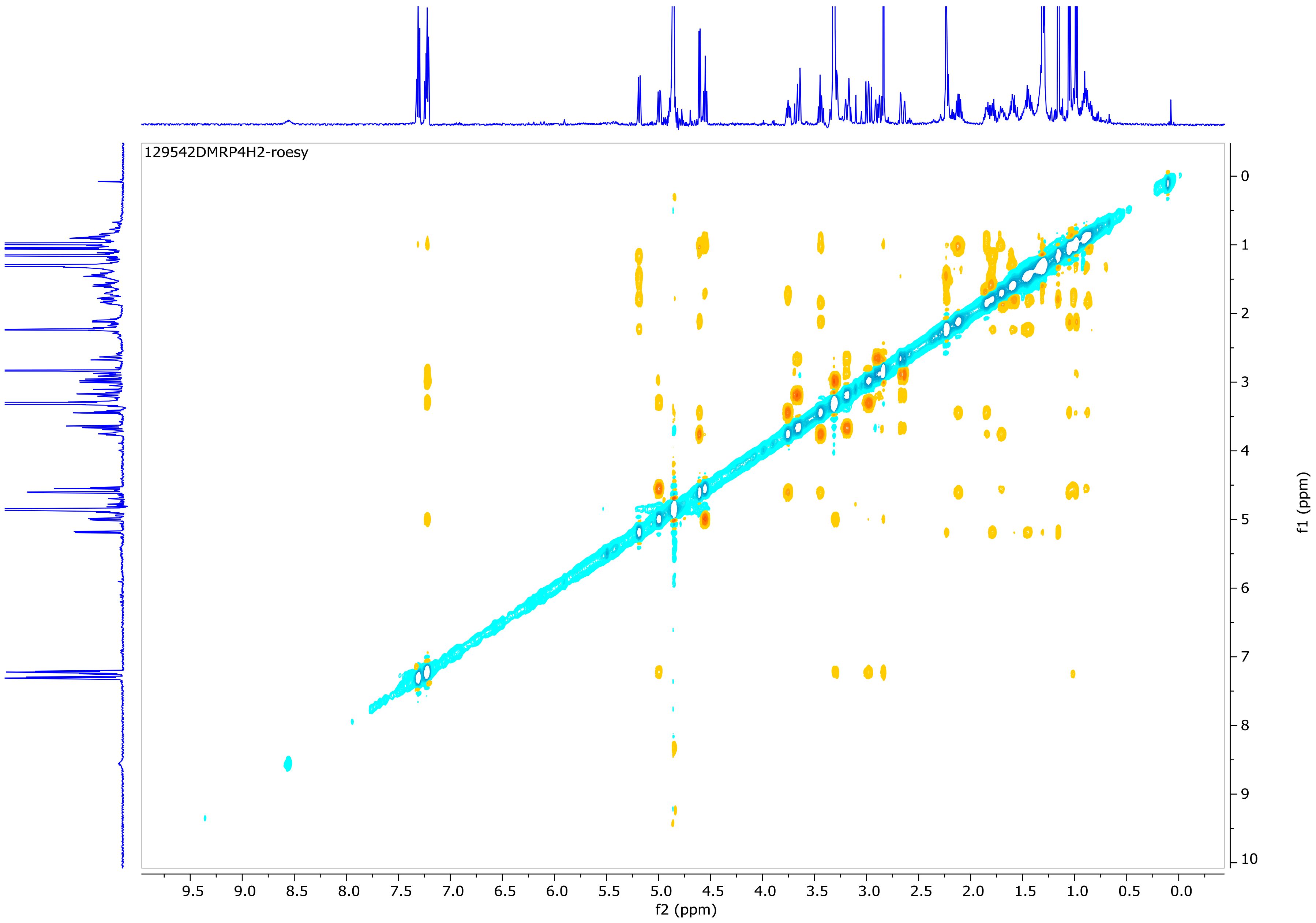

Figure S9. ROESY spectrum of **PM170453**. (CD_3OH).

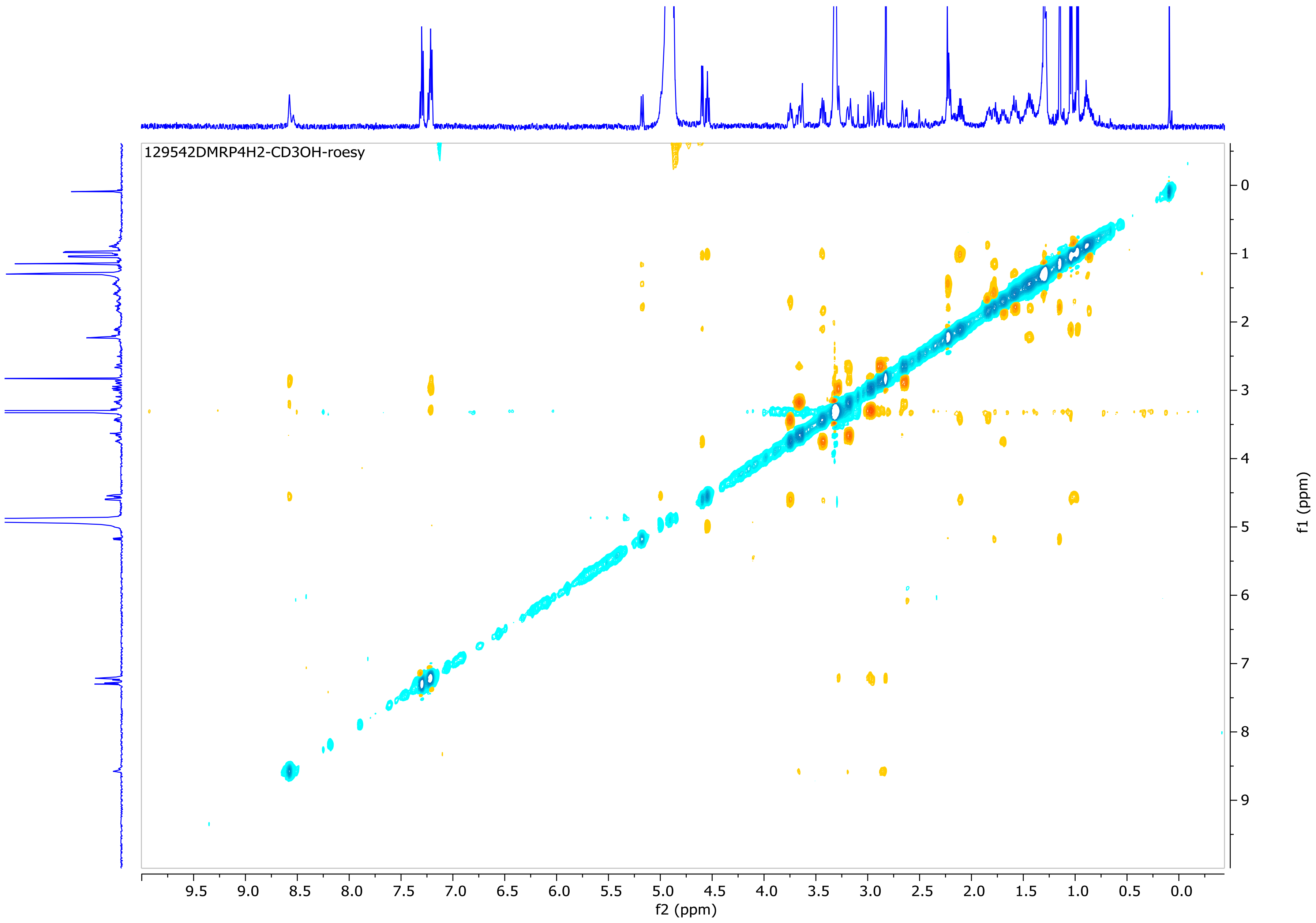

Figure S10. (+)-HRESITOFMS spectrum of **PM170453**.

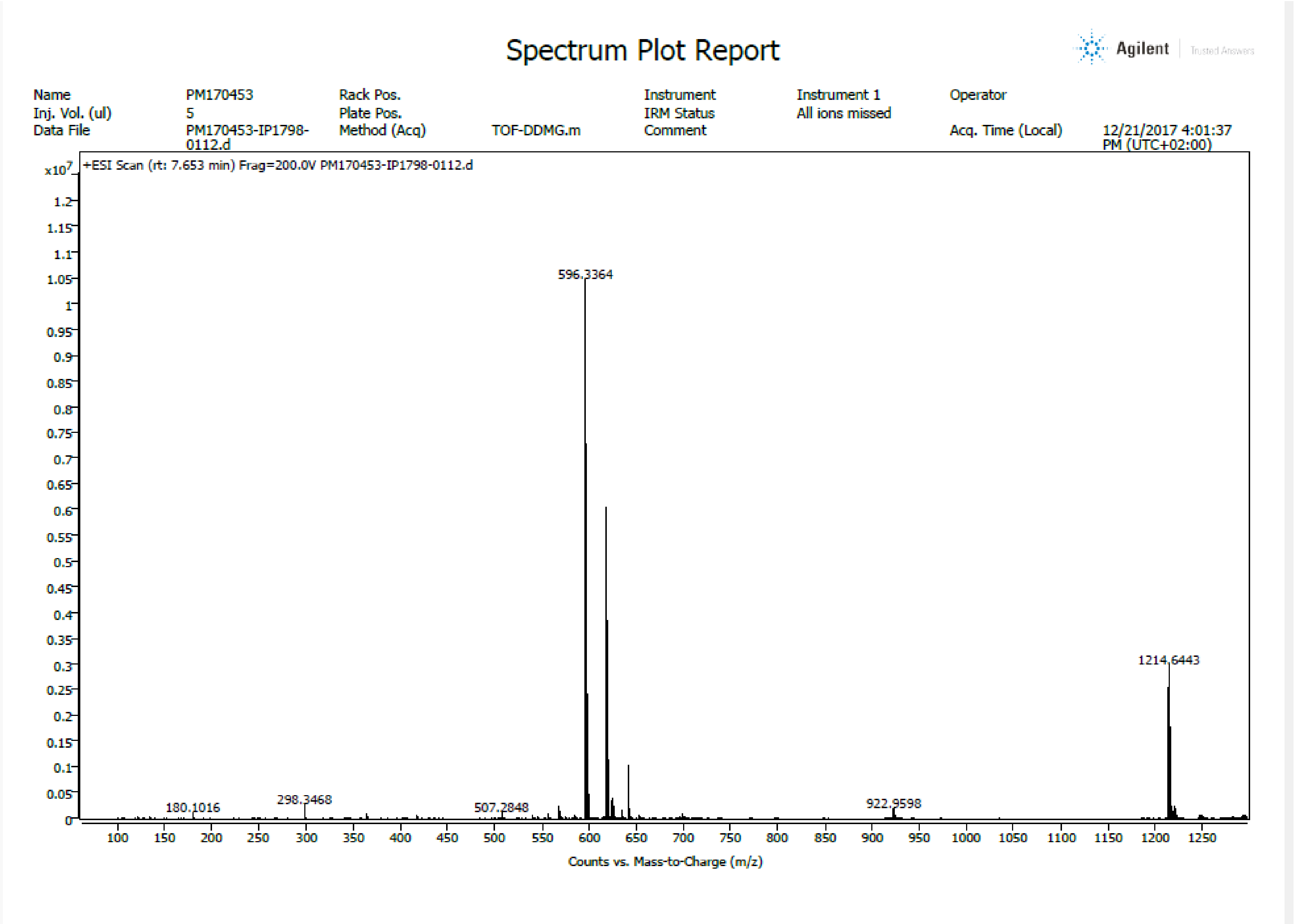

Figure S11. Dose-response curves for **1** in a panel of 4 tumour cell lines

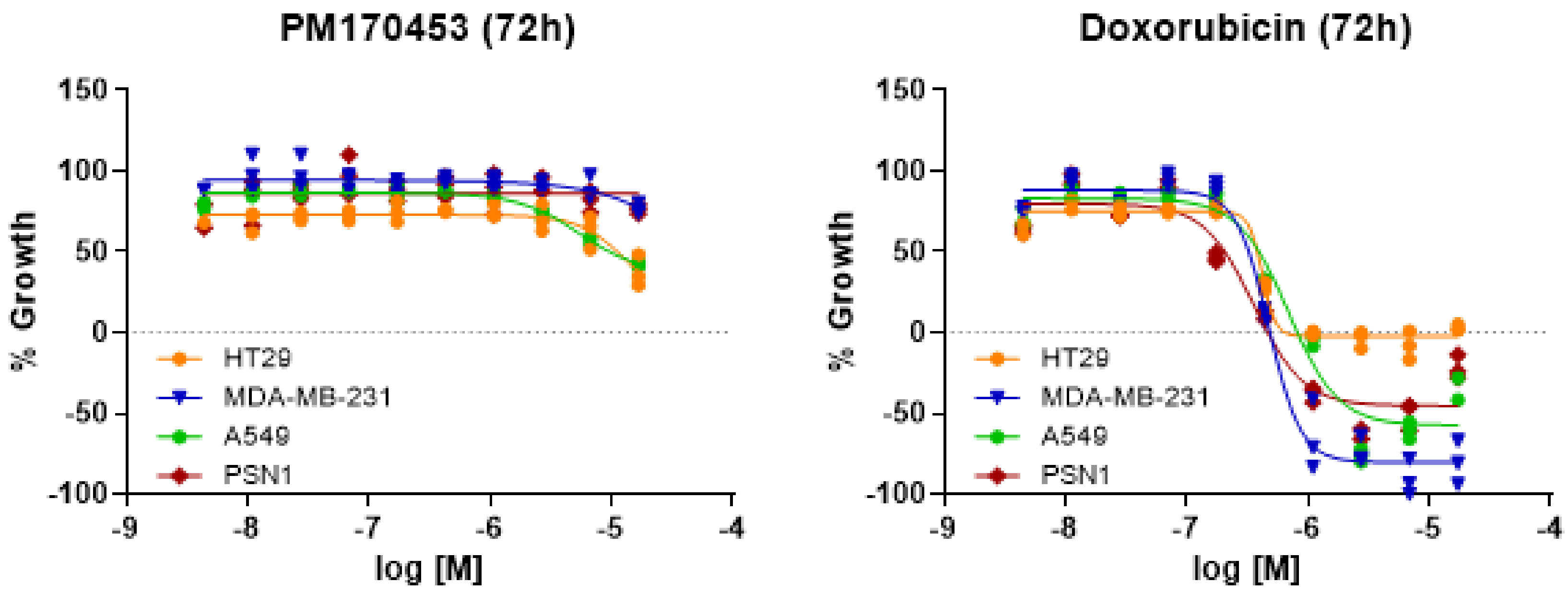

Figure S12. PD-1/PDL-1 interaction curves for **1**

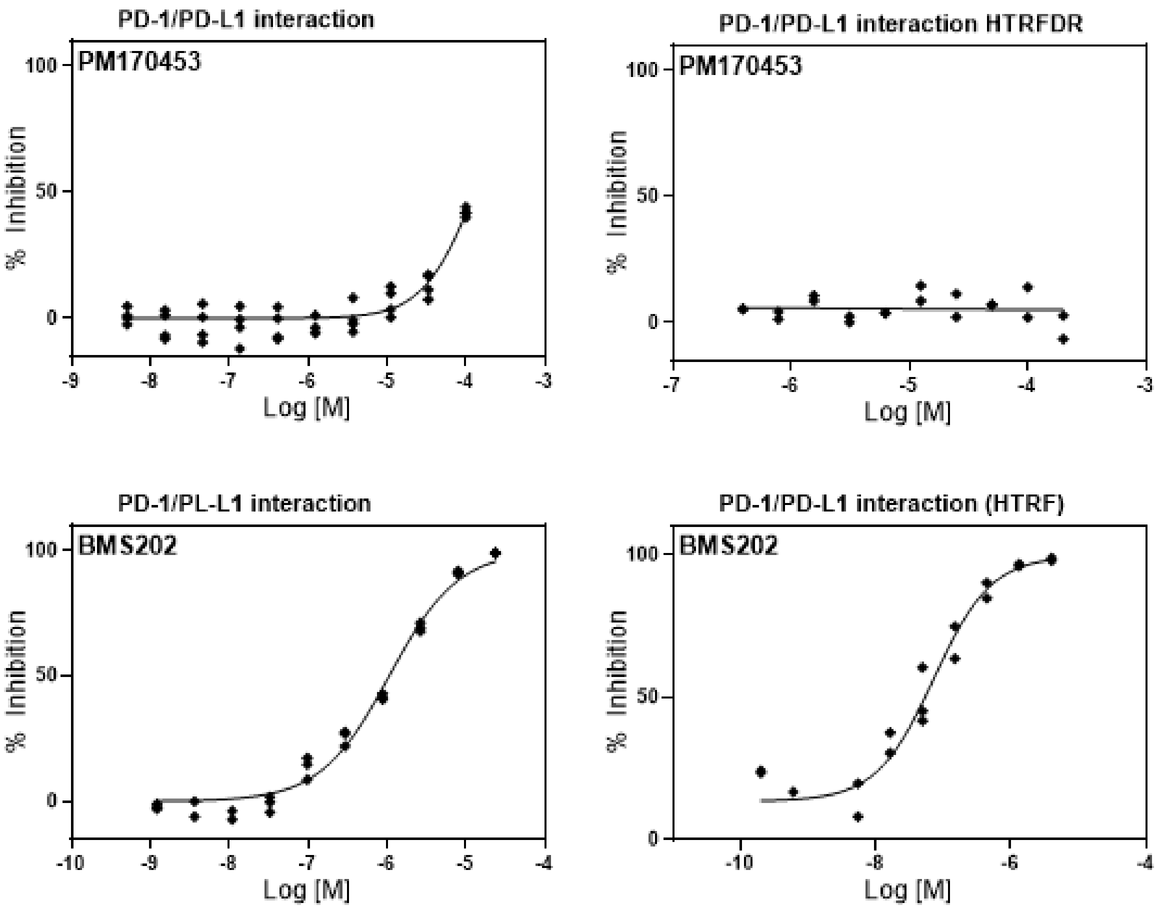

Figure S13. ^{1}H NMR spectrum of hex-5-ynal (**4**) (400 MHz, $CDCl_3$).

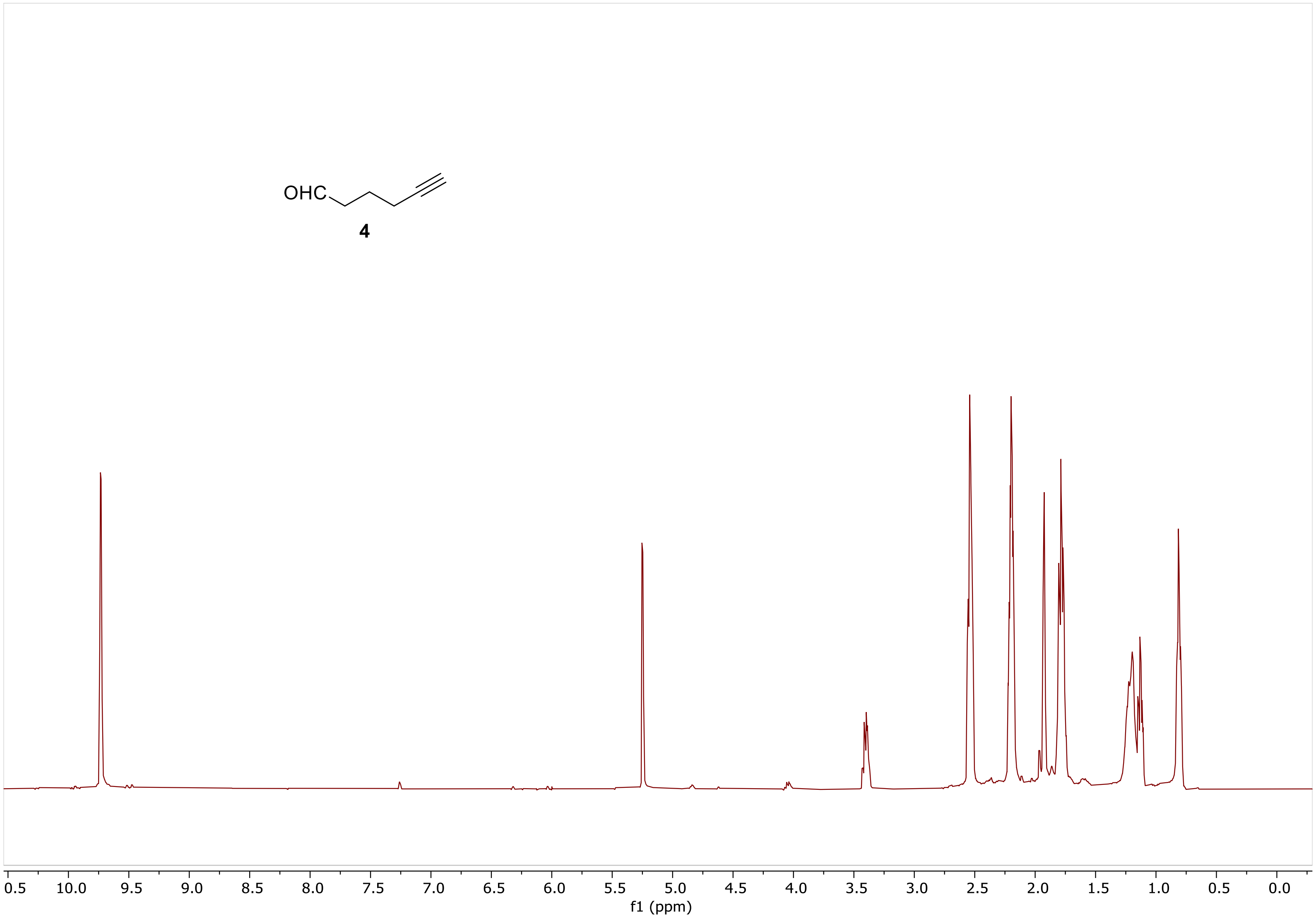

Figure S14. ^{13}C NMR spectrum of hex-5-ynal (**4**) (100 MHz, $CDCl_3$).

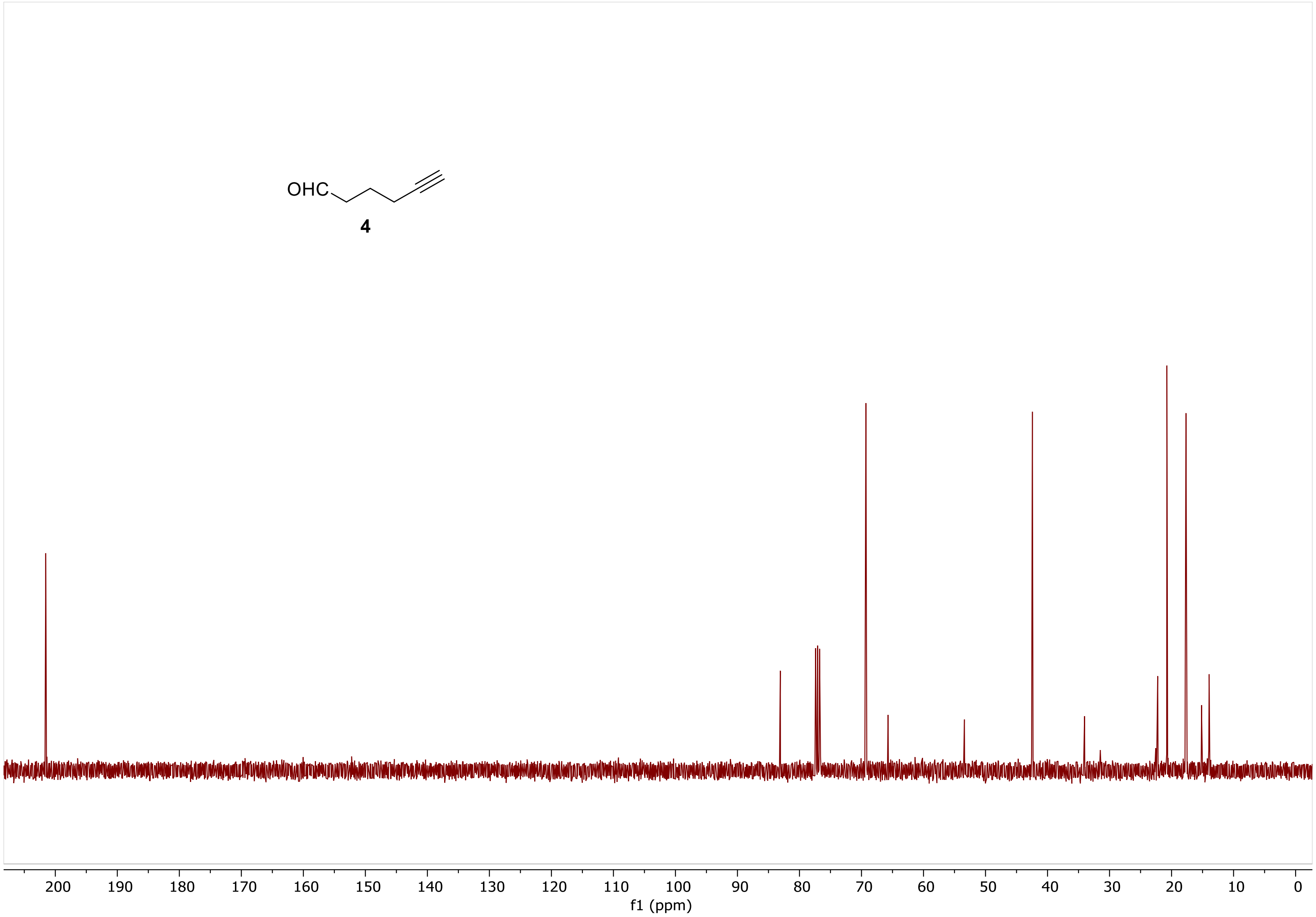

Figure S15. ^{1}H NMR spectrum of (*S*)-4-benzyl-3-isobutyryloxazolidin-2-one (**5**) (400 MHz, $CDCl_3$).

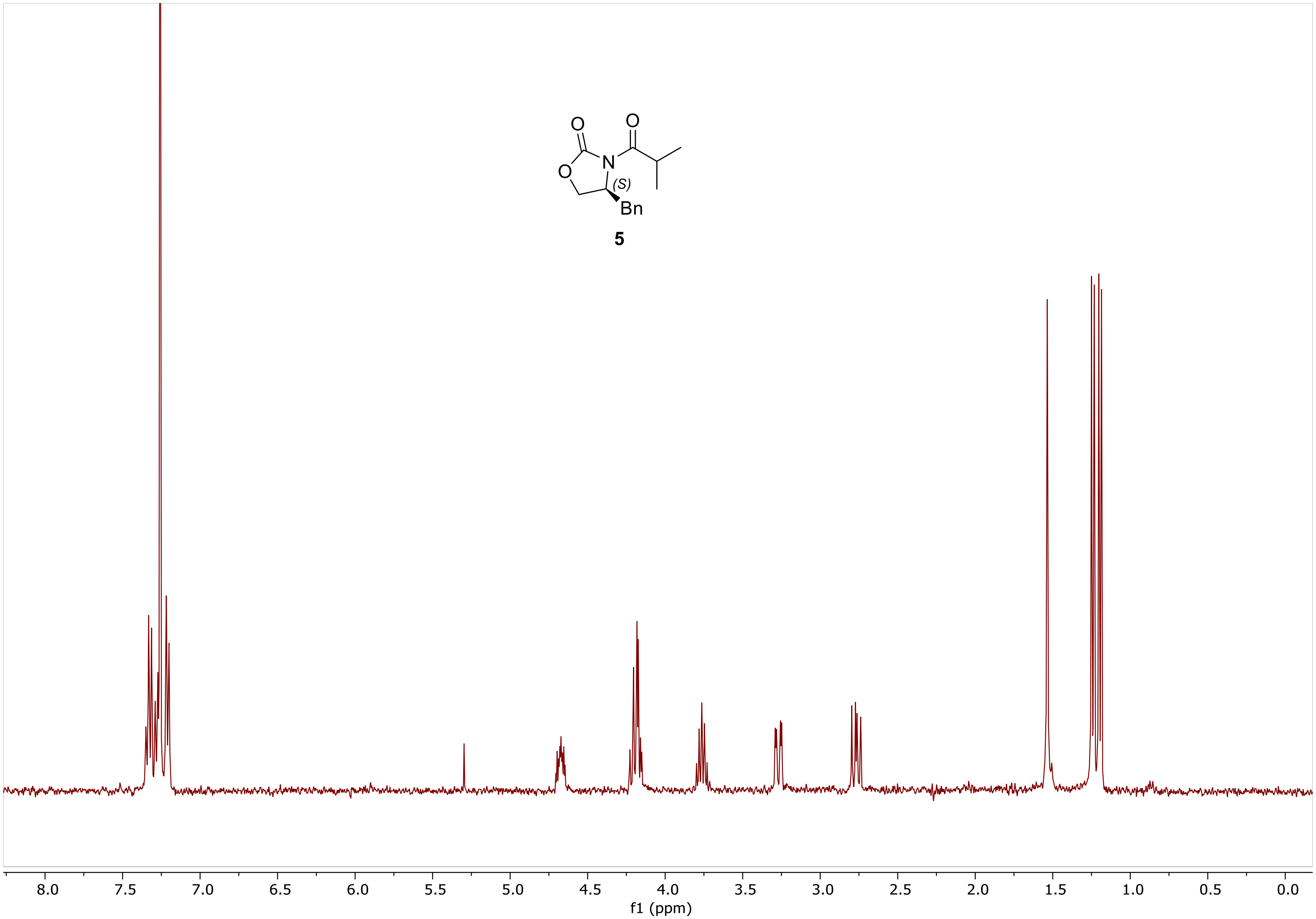

Figure S16. ^{13}C NMR spectrum of (*S*)-4-benzyl-3-isobutyryloxazolidin-2-one (**5**) (100 MHz, $CDCl_3$).

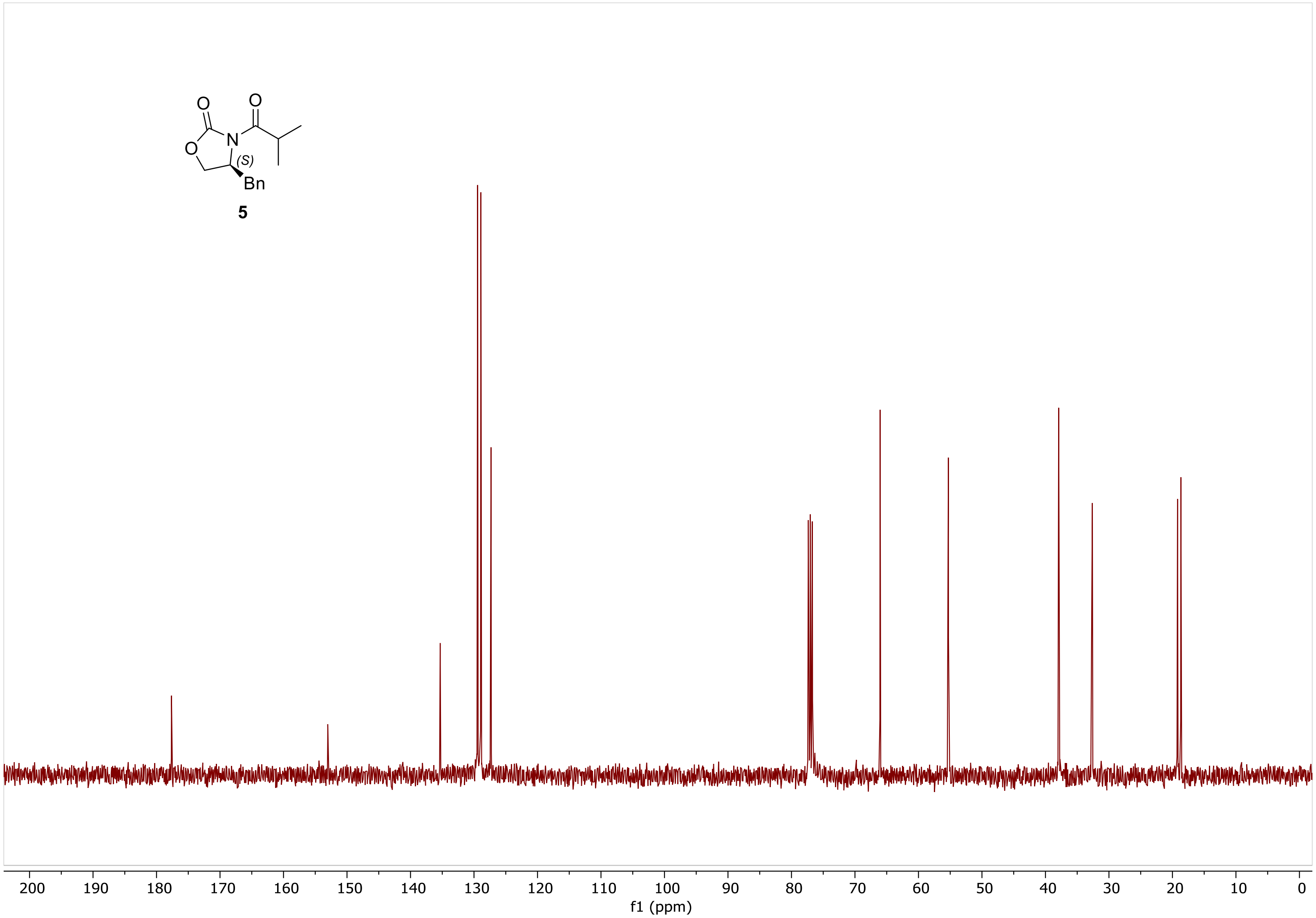

Figure S17. ^{1}H NMR spectrum of (*S*)-4-benzyl-3-((S)-3-hydroxy-2,2-dimethyloct-7-ynoyl)oxazolidin-2-one (**6**) (400 MHz, $CDCl_3$).

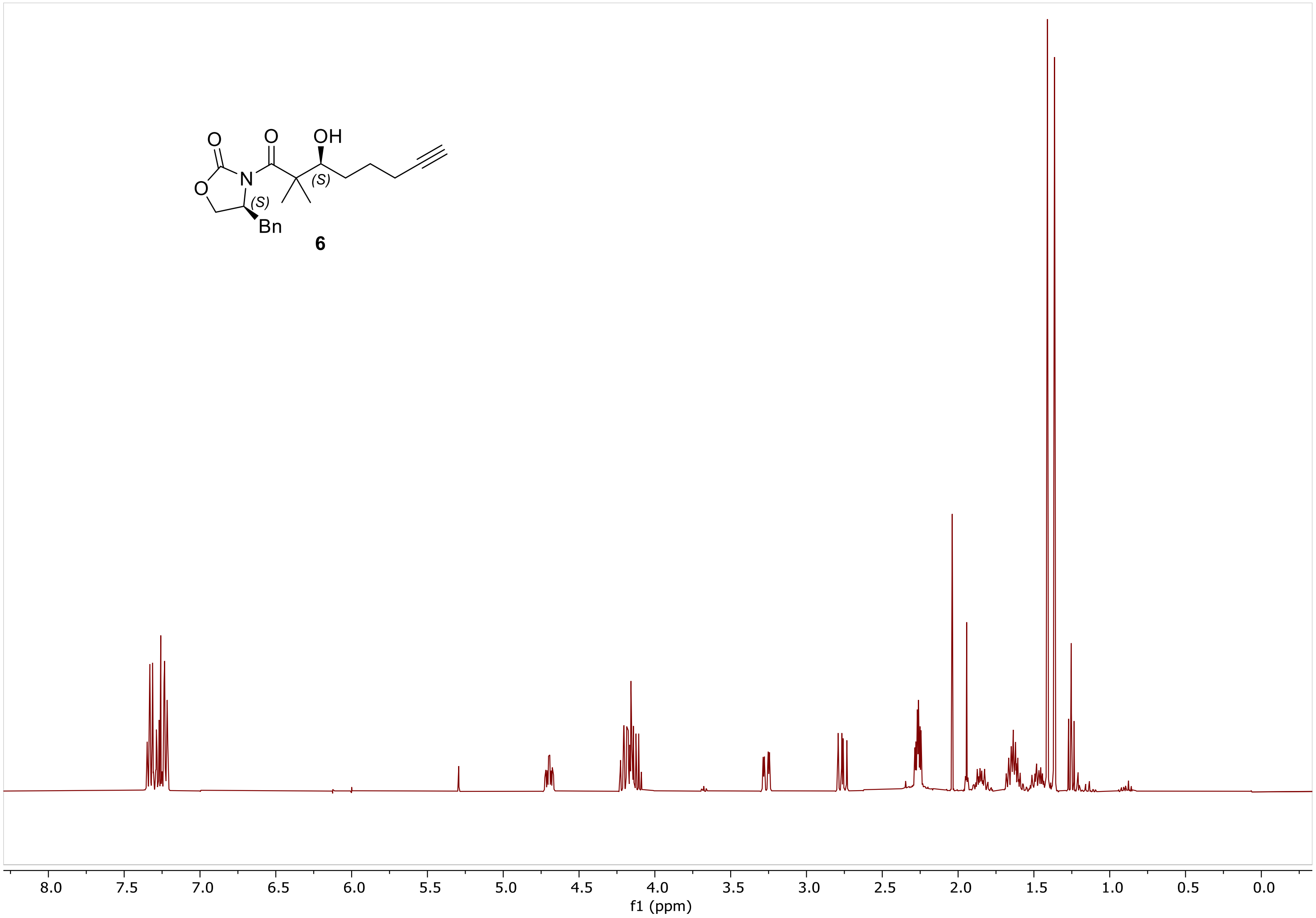

Figure S18. ^{13}C NMR spectrum of (*S*)-4-benzyl-3-((S)-3-hydroxy-2,2-dimethyloct-7-ynoyl)oxazolidin-2-one (**6**) (100 MHz, $CDCl_3$).

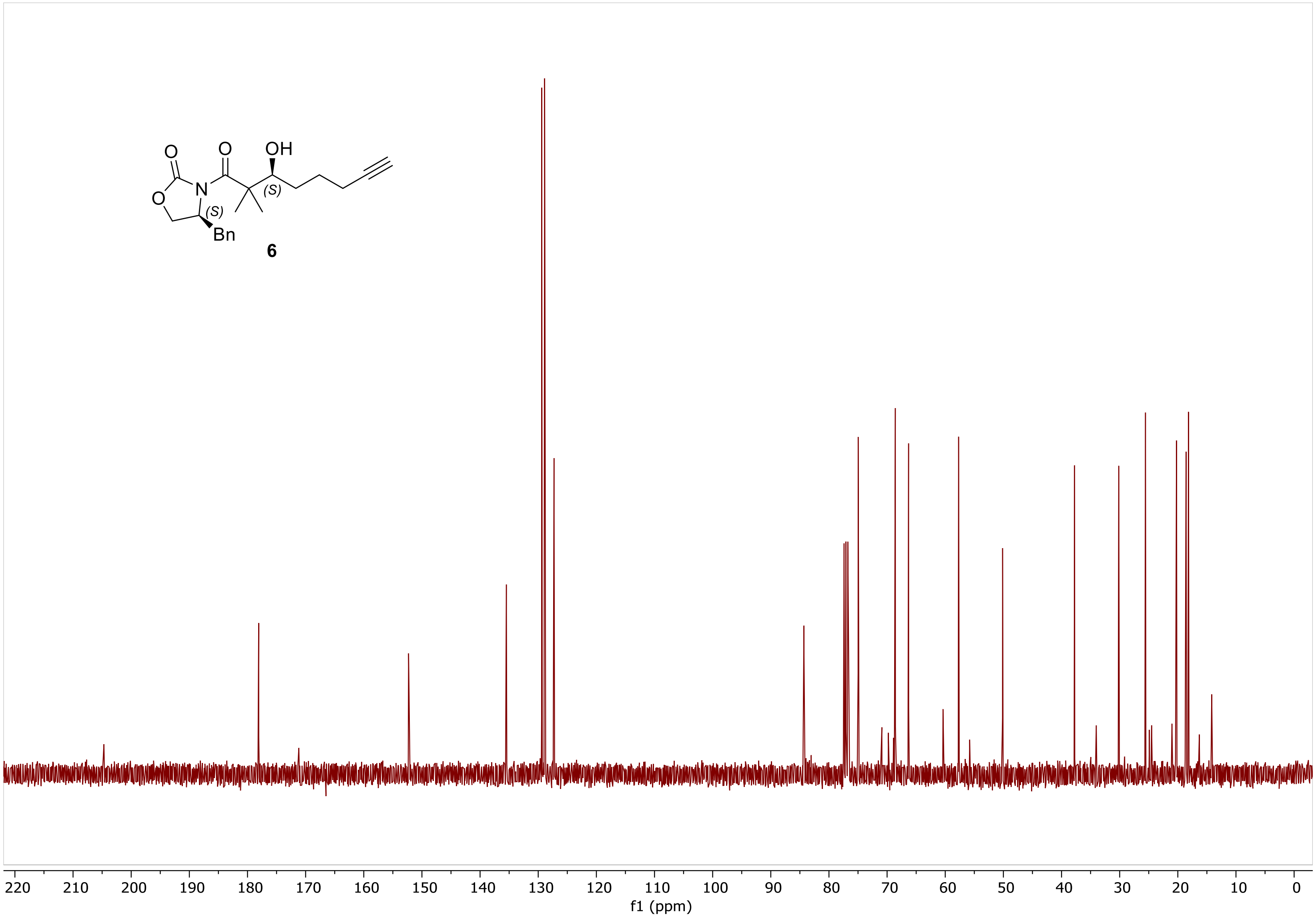

Figure S19. ^{1}H NMR spectrum of (*S*)-3-hydroxy-2,2-dimethyloct-7-ynoic acid (**7**) (400 MHz, $CDCl_3$).

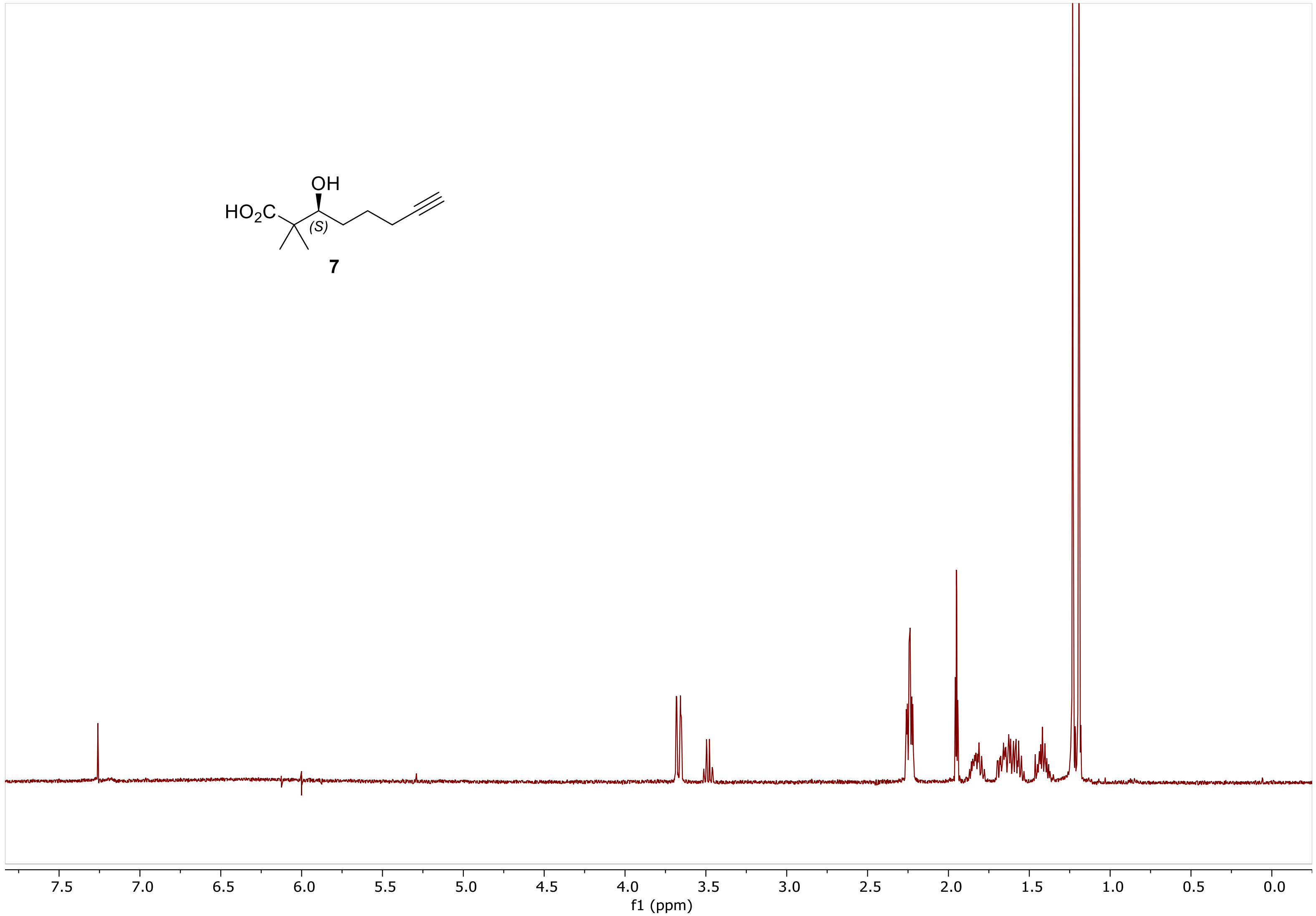

Figure S20. ^{13}C NMR spectrum of (*S*)-3-hydroxy-2,2-dimethyloct-7-ynoic acid (**7**) (100 MHz, $CDCl_3$).

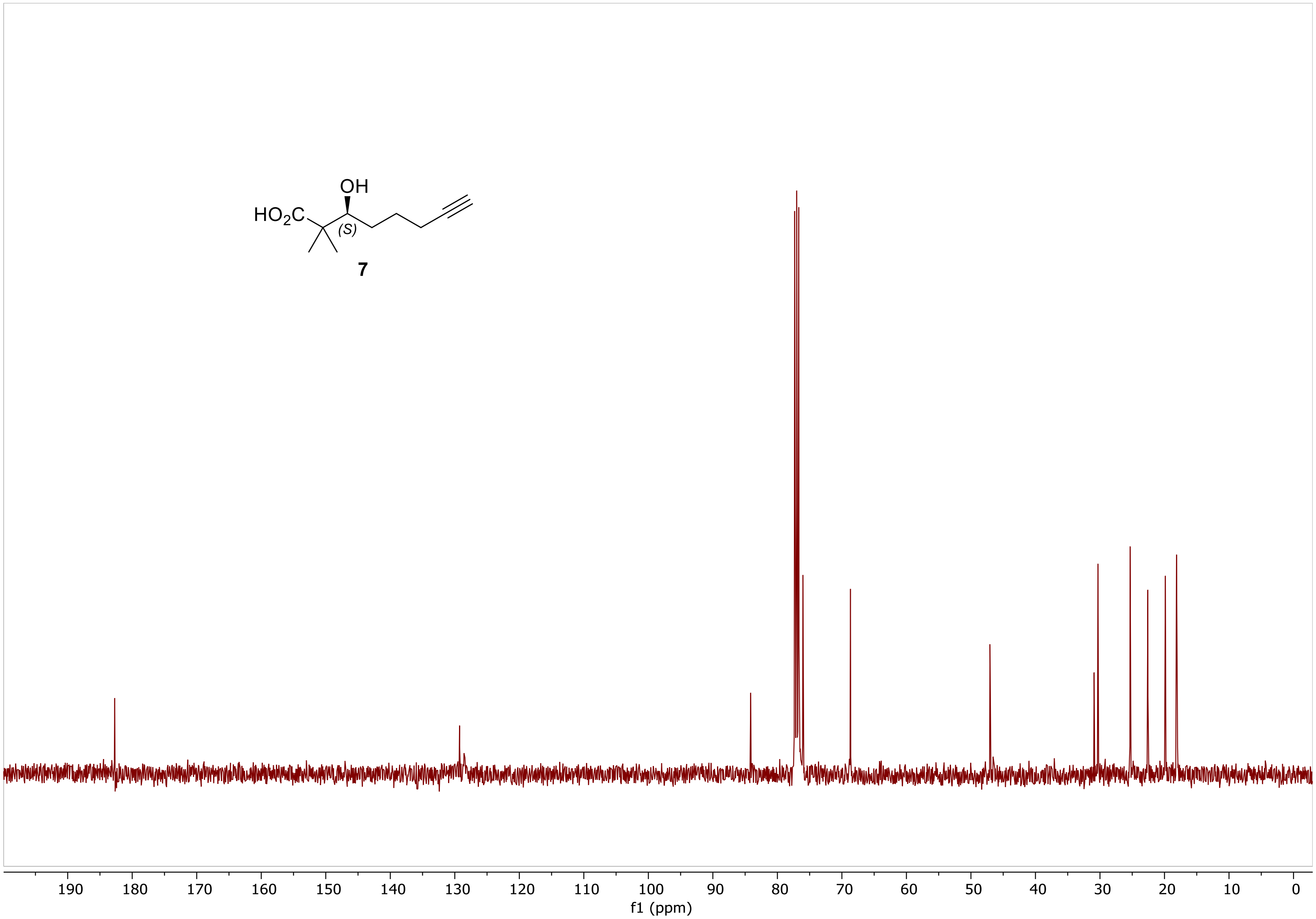

Figure S21. ^{1}H NMR spectrum of *tert*-butyldimethylsilyl (*S*)-3-((*tert*-butyldimethylsilyl)oxy)-2,2-dimethyloct-7-ynoate (**8**) (400 MHz, $CDCl_3$).

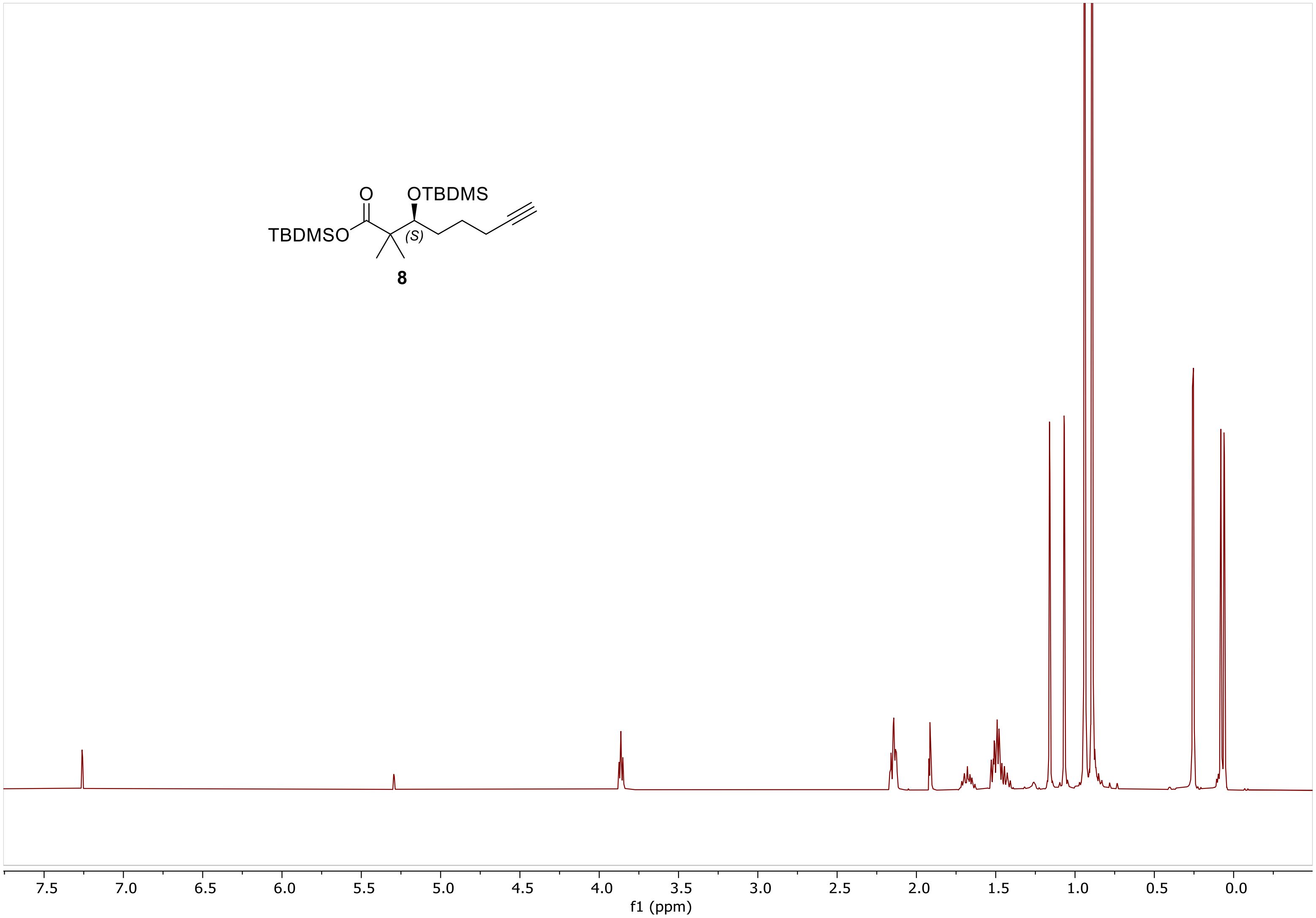

Figure S22. ^{13}C NMR spectrum of *tert*-butyldimethylsilyl (*S*)-3-((*tert*-butyldimethylsilyl)oxy)-2,2-dimethyloct-7-ynoate (**8**) (100 MHz, $CDCl_3$).

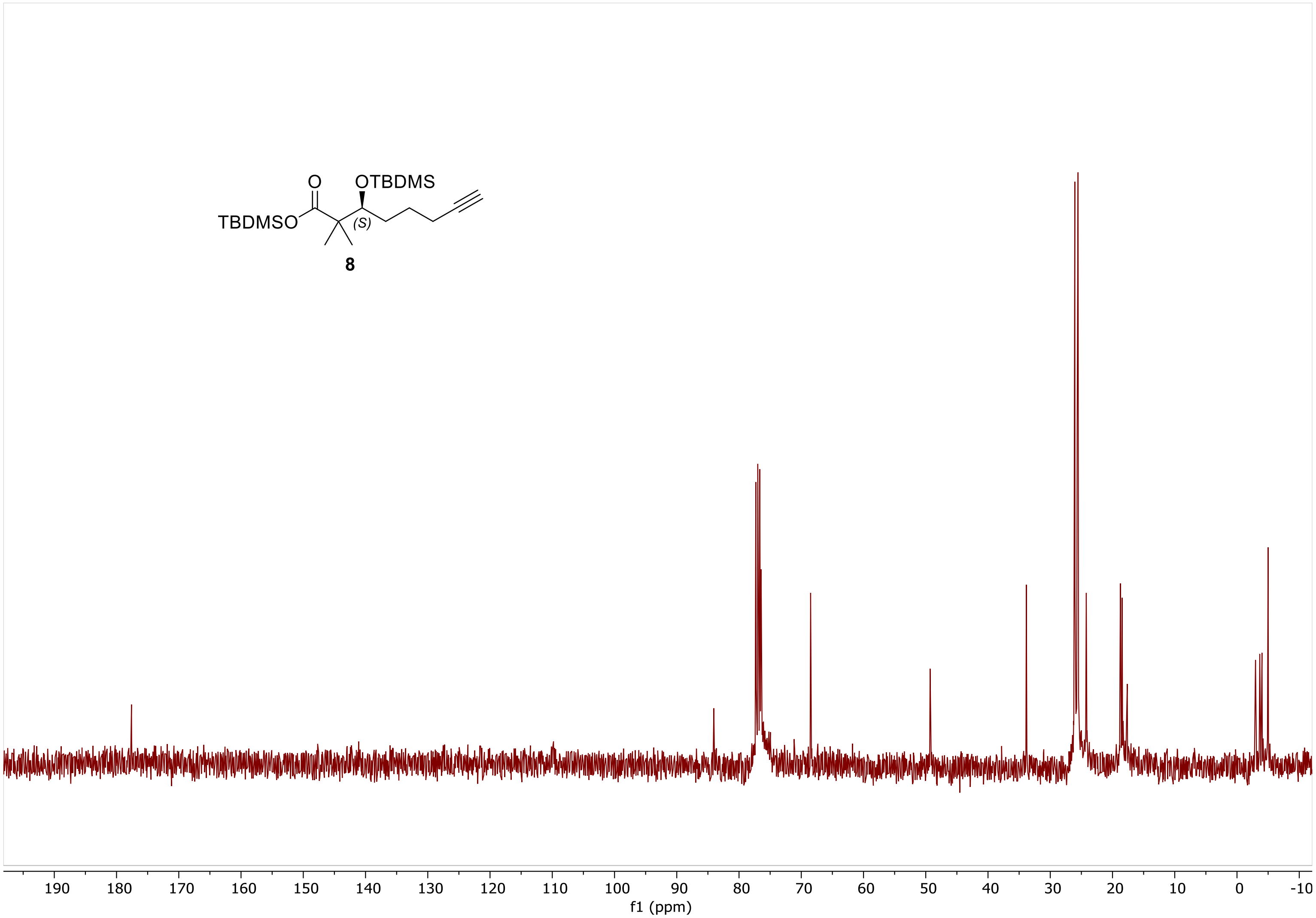

Figure S23. ^{1}H NMR spectrum of (*S*)-3-((*tert*-butyldimethylsilyl)oxy)-2,2-dimethyloct-7-ynoic acid (**2**) (400 MHz, CD_3OD).

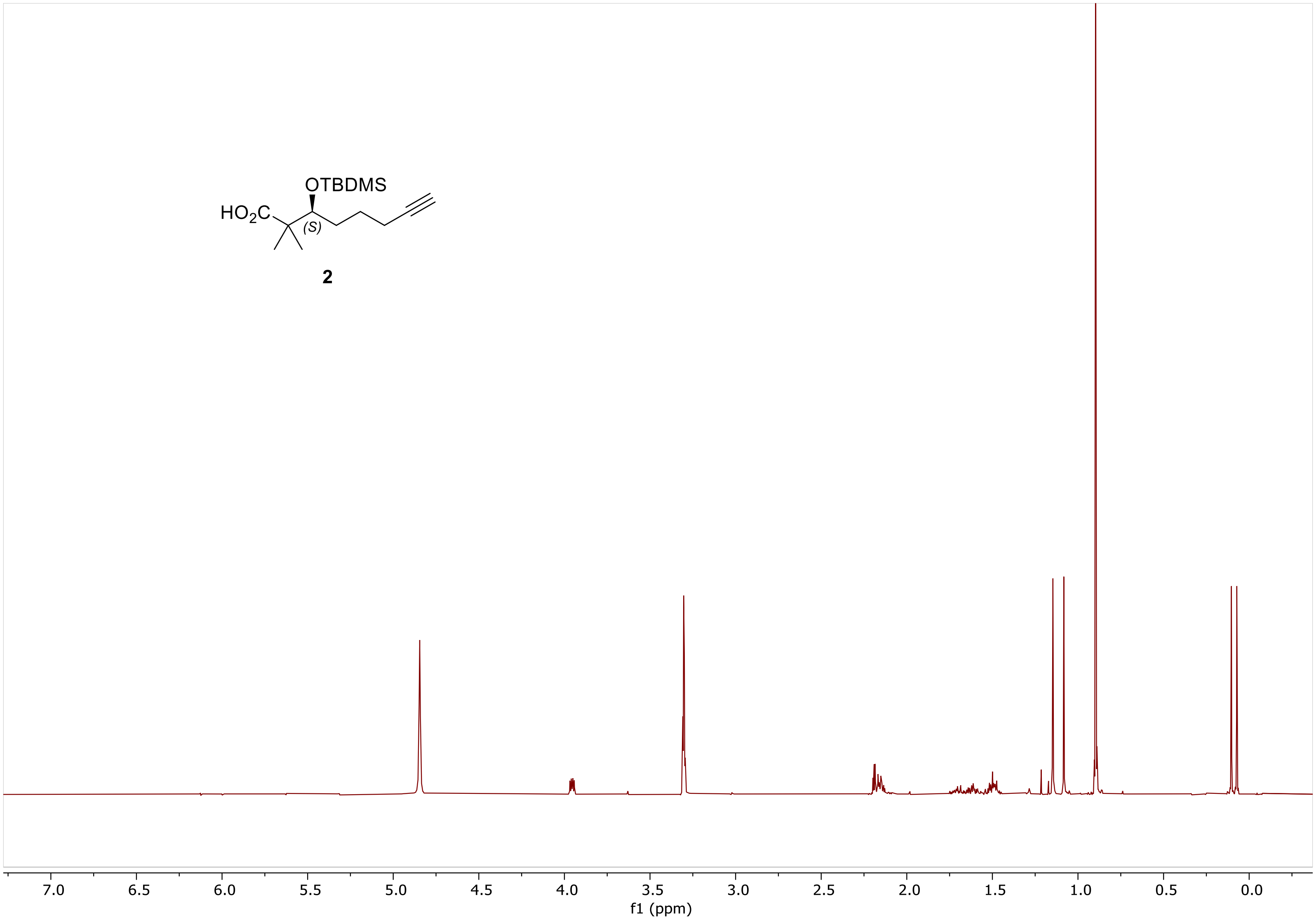

Figure S24. ^{13}C NMR spectrum of (*S*)-3-((*tert*-butyldimethylsilyl)oxy)-2,2-dimethyloct-7-ynoic acid (**2**) (100 MHz, CD_3OD).

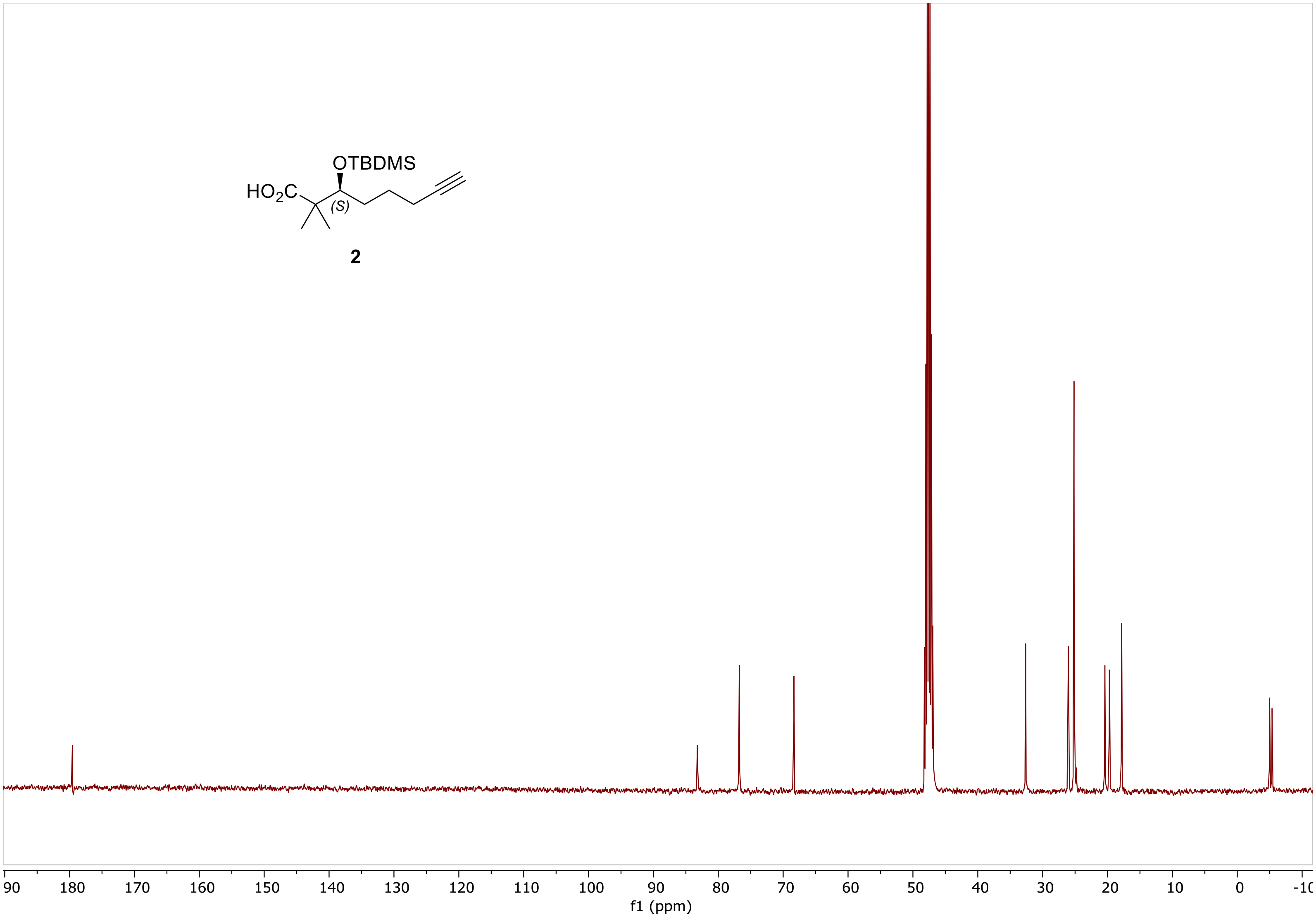

Figure S25. ^{1}H NMR spectrum of *tert*-butyldimethylsilyl (*S*)-2-((*tert*-butyldimethylsilyl)oxy)-3-methylbutanoate (**9**) (400 MHz, $CDCl_3$).

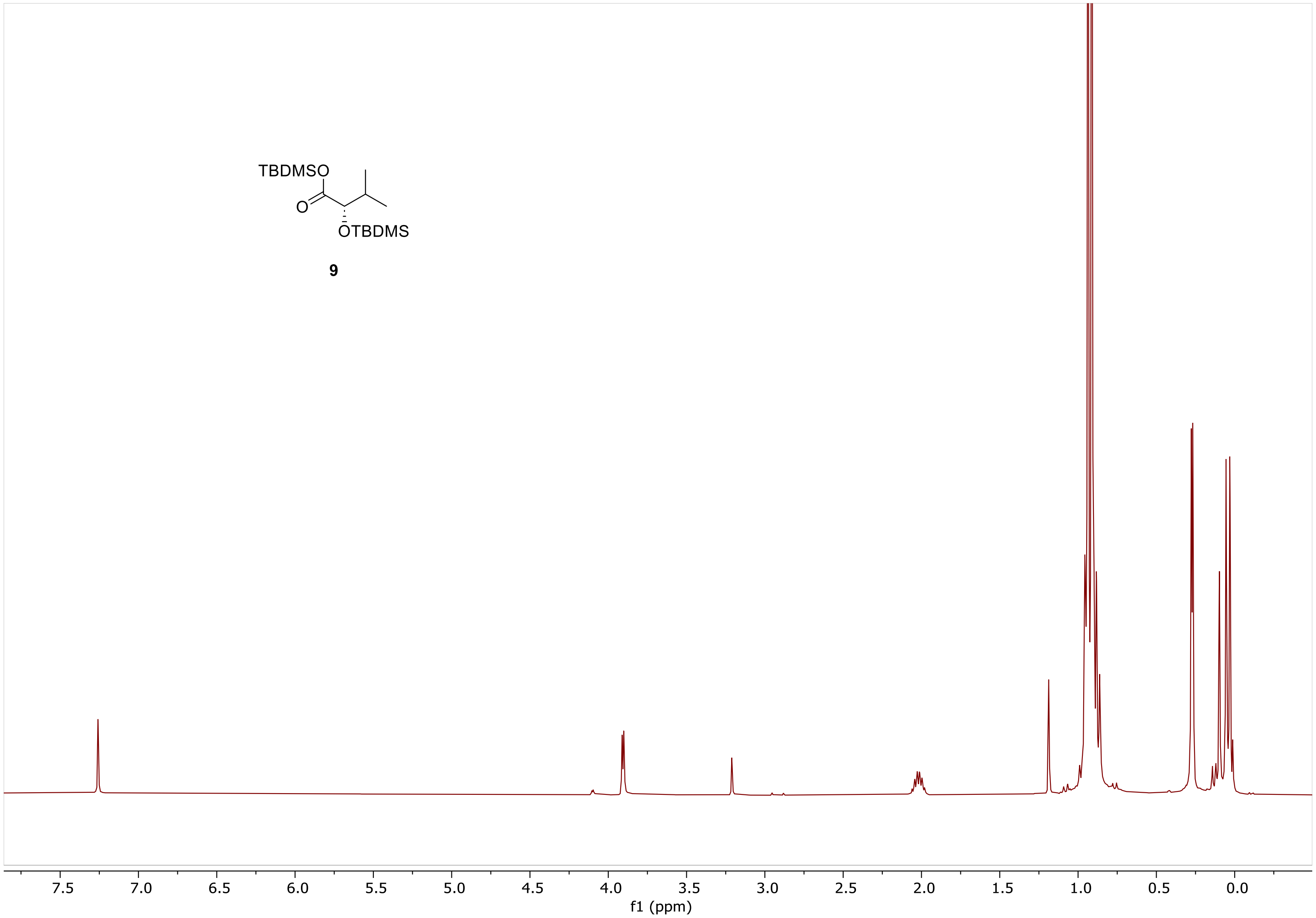

Figure S26. ^{13}C NMR spectrum of *tert*-butyldimethylsilyl (*S*)-2-((*tert*-butyldimethylsilyl)oxy)-3-methylbutanoate (**9**) (100 MHz, $CDCl_3$).

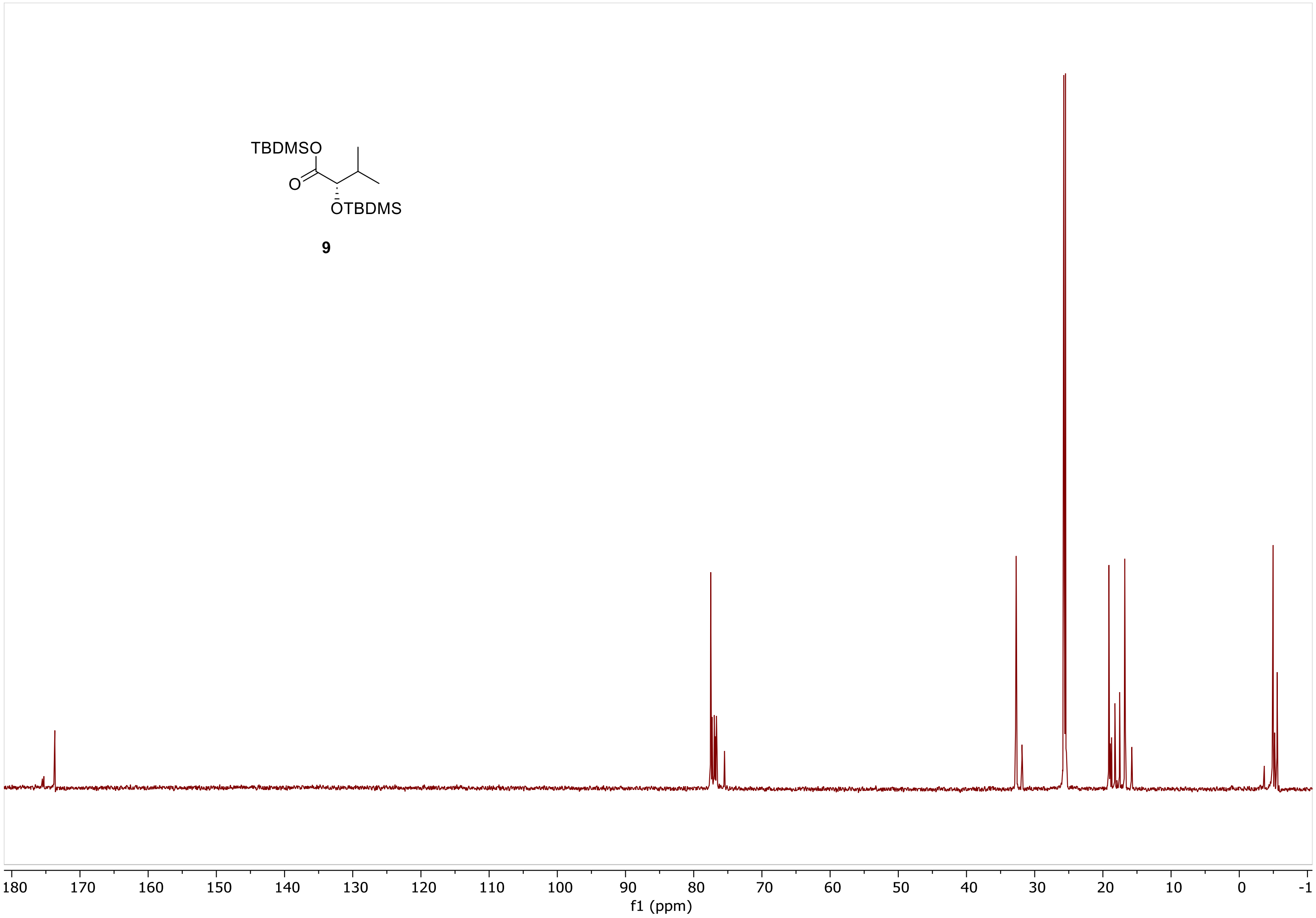

Figure S27. ^{1}H NMR spectrum of (*S*)-2-((*tert*-butyldimethylsilyl)oxy)-3-methylbutanoic acid (**10**) (400 MHz, $CDCl_3$).

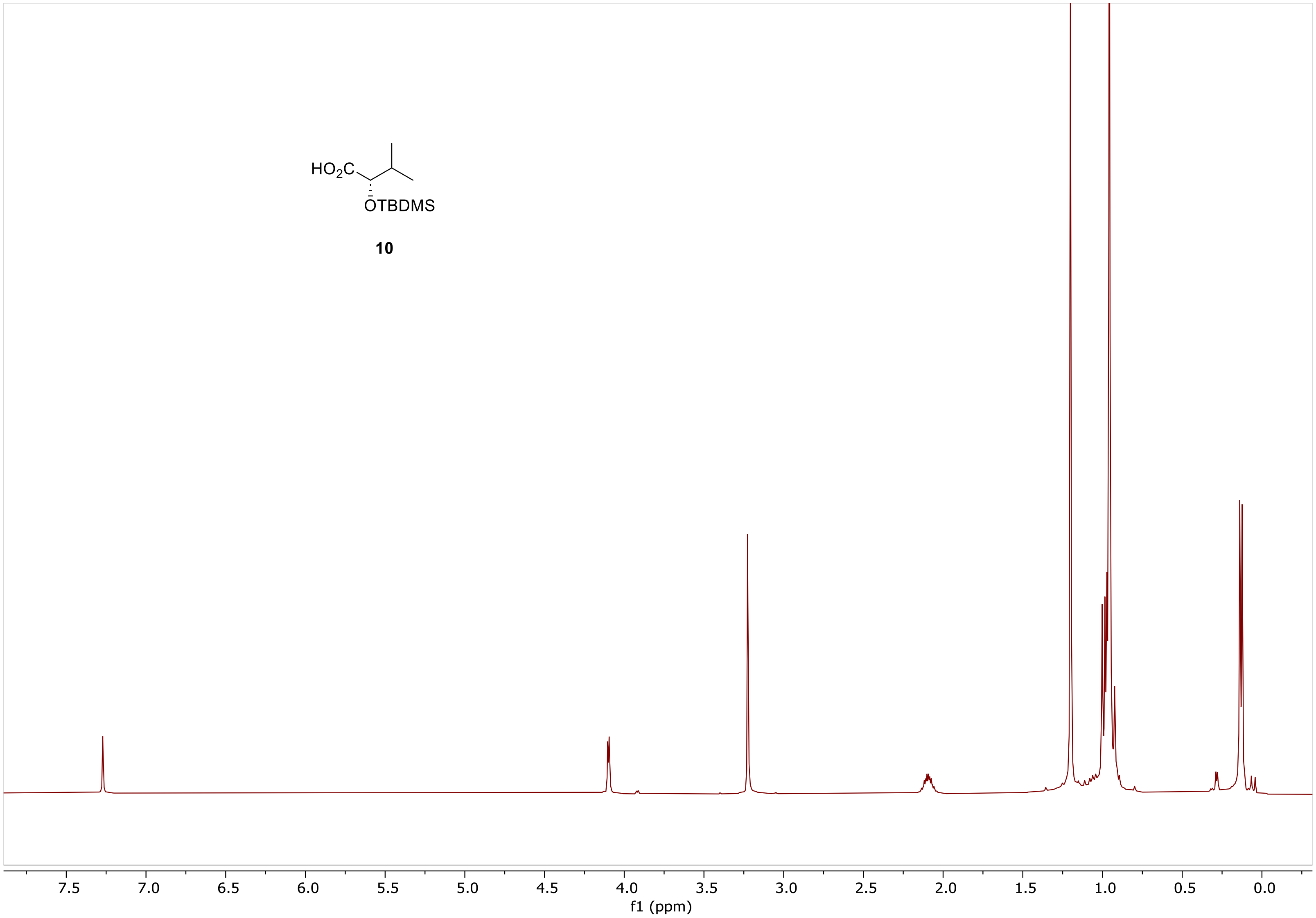

Figure S29. ^{13}C NMR spectrum of (*S*)-2-((*tert*-butyldimethylsilyl)oxy)-3-methylbutanoic acid (**10**) (100 MHz, $CDCl_3$).

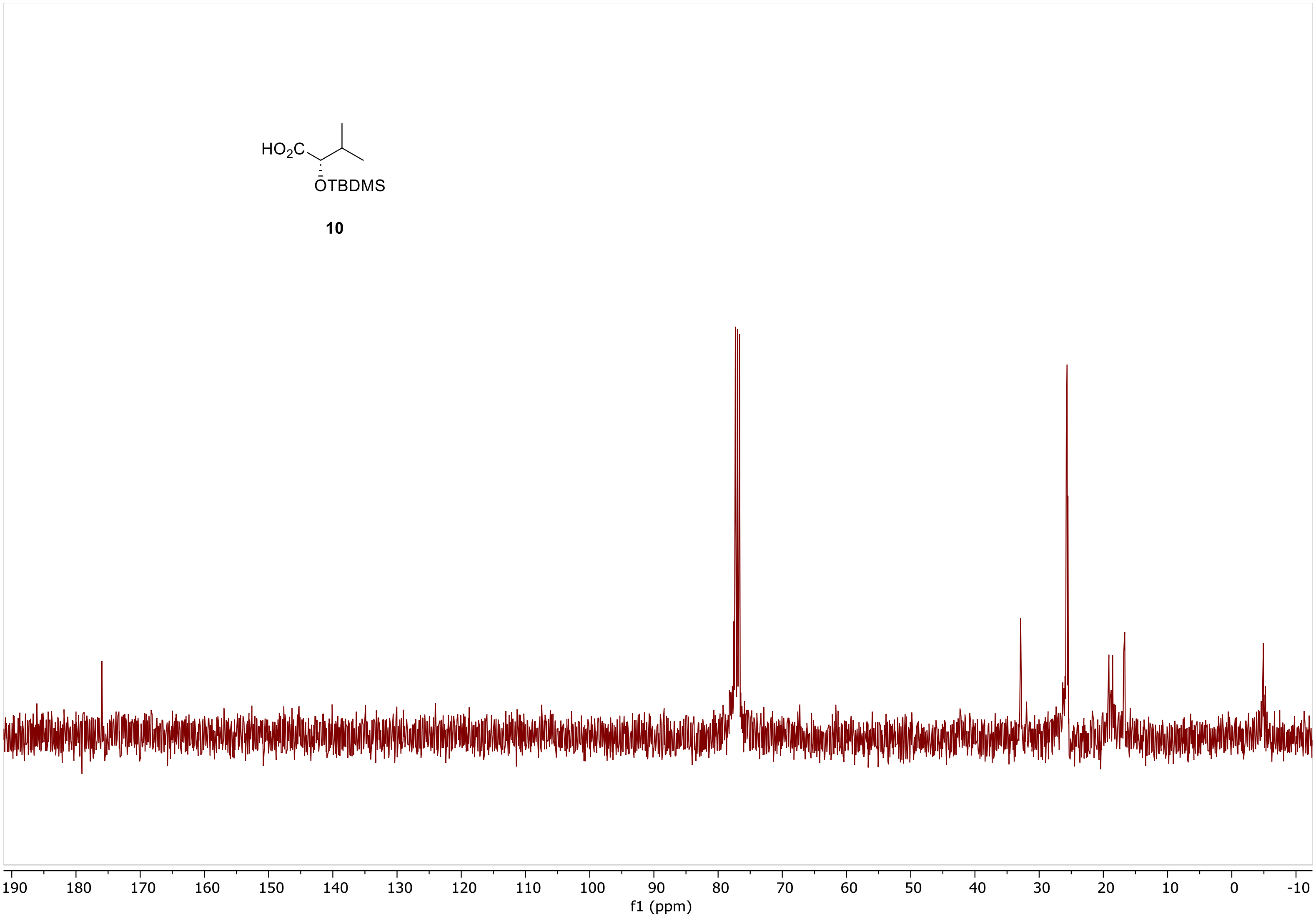

Figure S29. ^{1}H NMR spectrum of benzyl ((*S*)-2-((*tert*-butyldimethylsilyl)oxy)-3-methylbutanoyl)-L-prolinate (**11**) (400 MHz, $CDCl_3$).

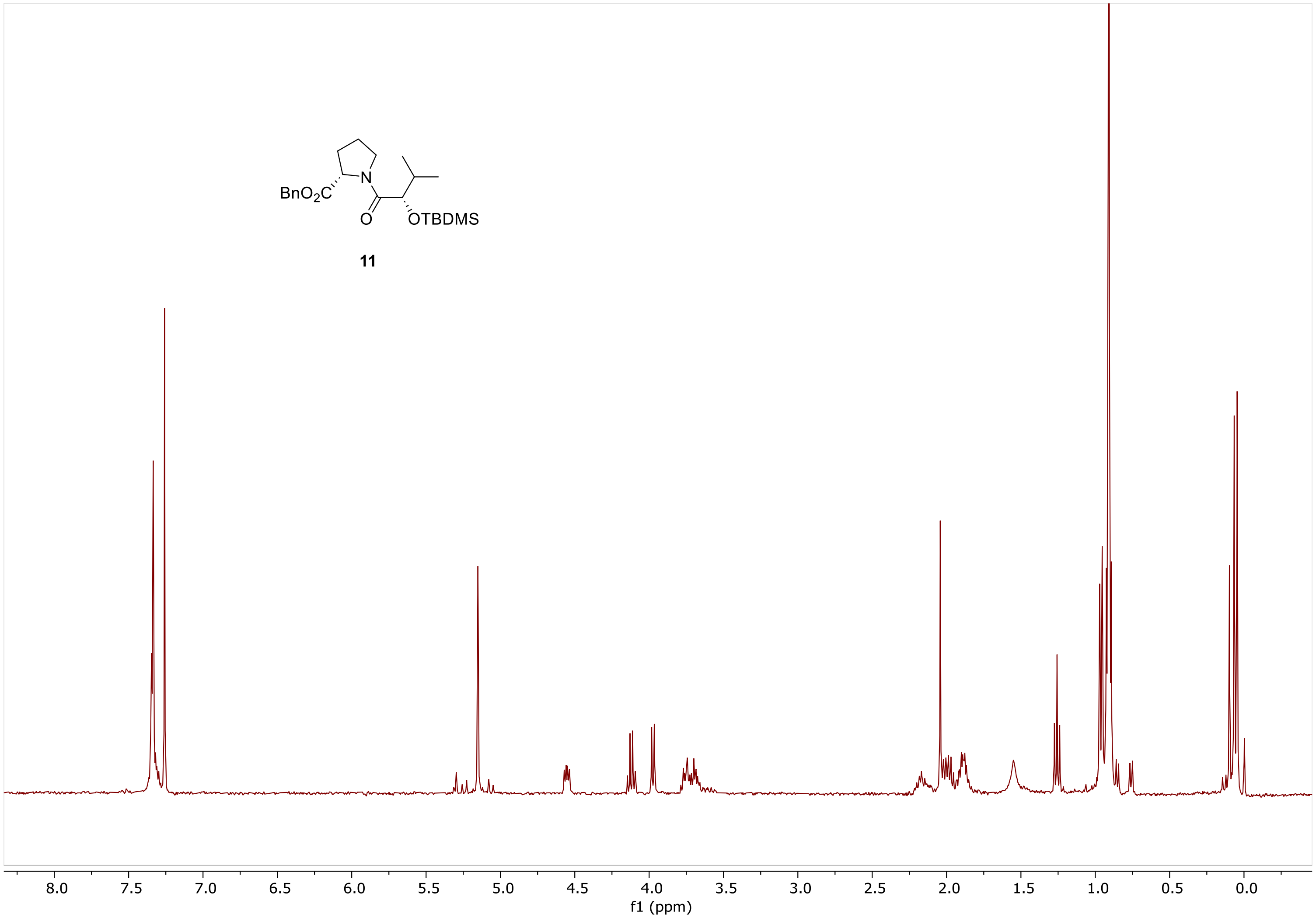

Figure S30. ^{13}C NMR spectrum of benzyl ((*S*)-2-((*tert*-butyldimethylsilyl)oxy)-3-methylbutanoyl)-L-prolinate (**11**) (100 MHz, $CDCl_3$).

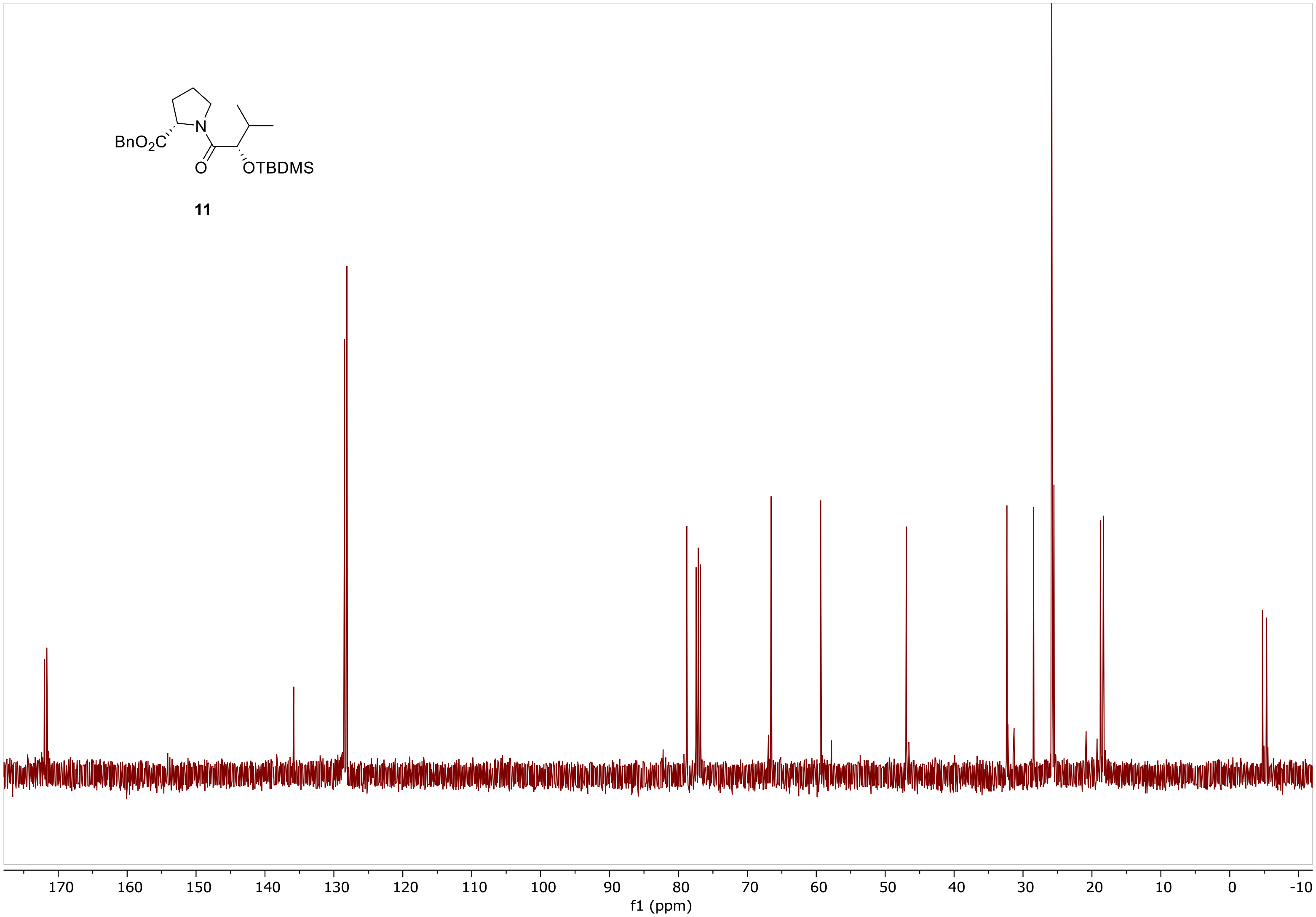

Figure S31. ^{1}H NMR spectrum of ((*S*)-2-((*tert*-butyldimethylsilyl)oxy)-3-methylbutanoyl)-L-proline (**12**) (400 MHz, CD_3OD).

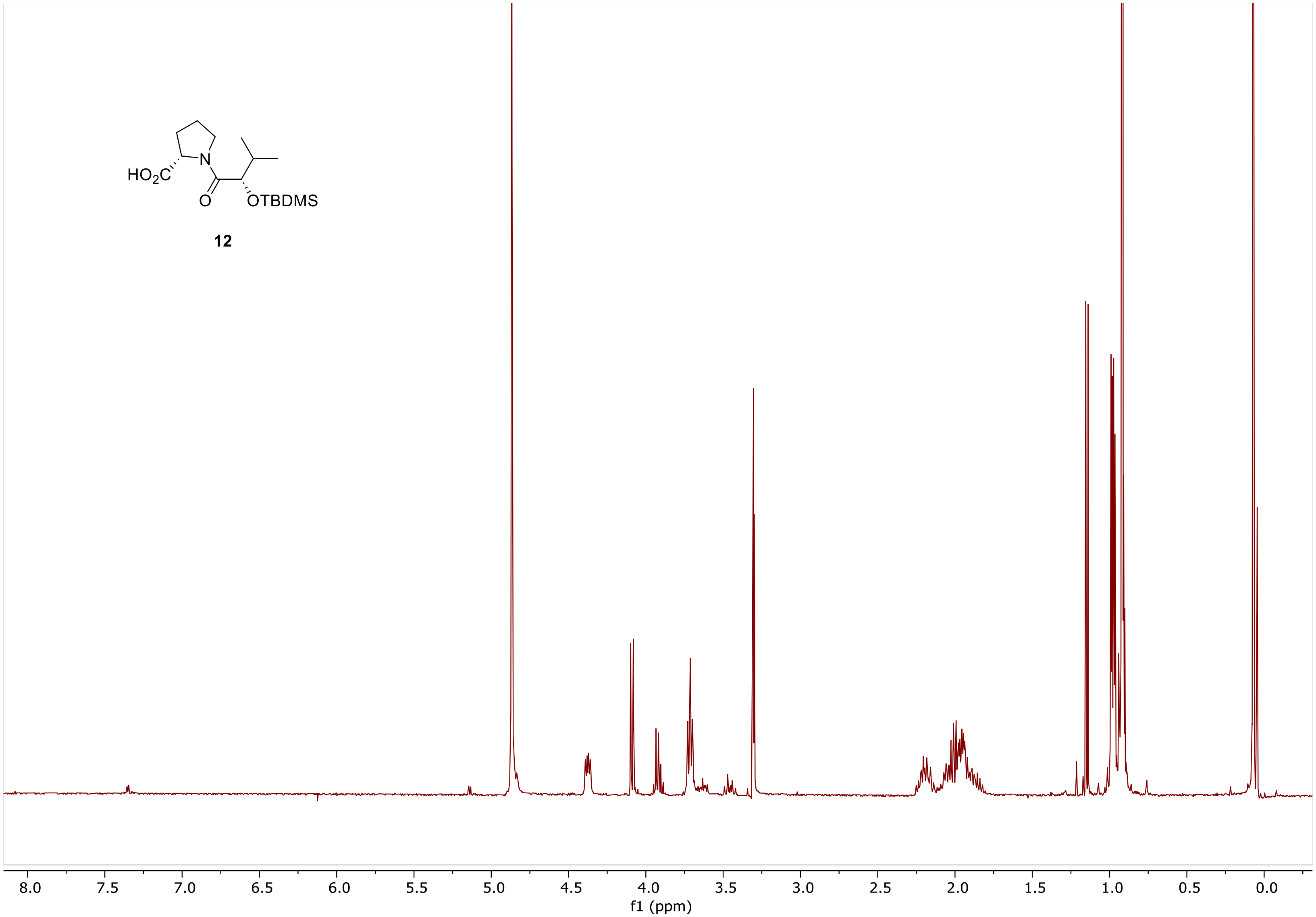

Figure S32. ^{13}C NMR spectrum of ((*S*)-2-((*tert*-butyldimethylsilyl)oxy)-3-methylbutanoyl)-L-proline (**12**) (100 MHz, CD_3OD).

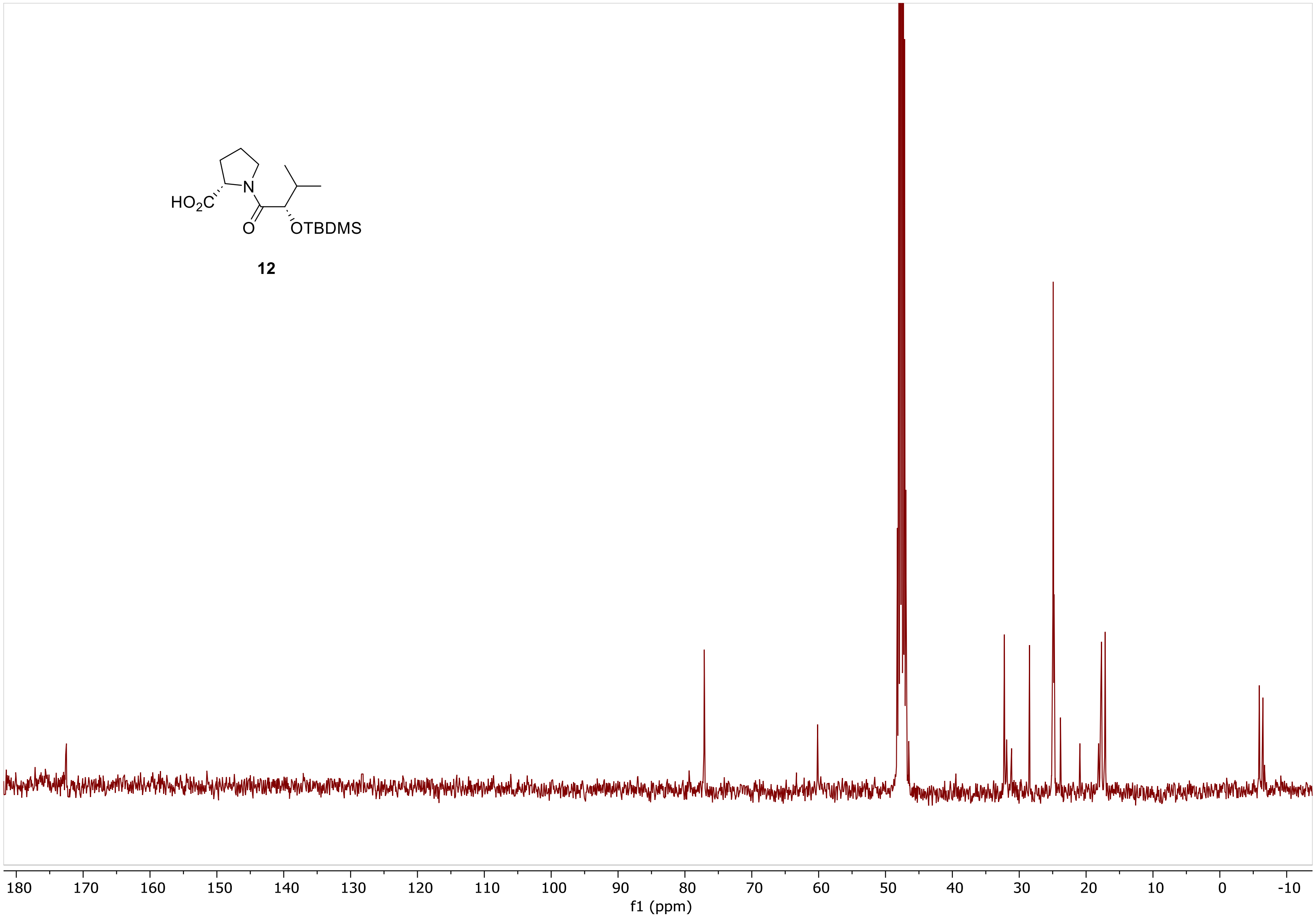

Figure S33. ^{1}H NMR spectrum of allyl *N*-(*tert*-butoxycarbonyl)-*N*-methyl-L-phenylalaninate (**13**) (400 MHz, $CDCl_3$).

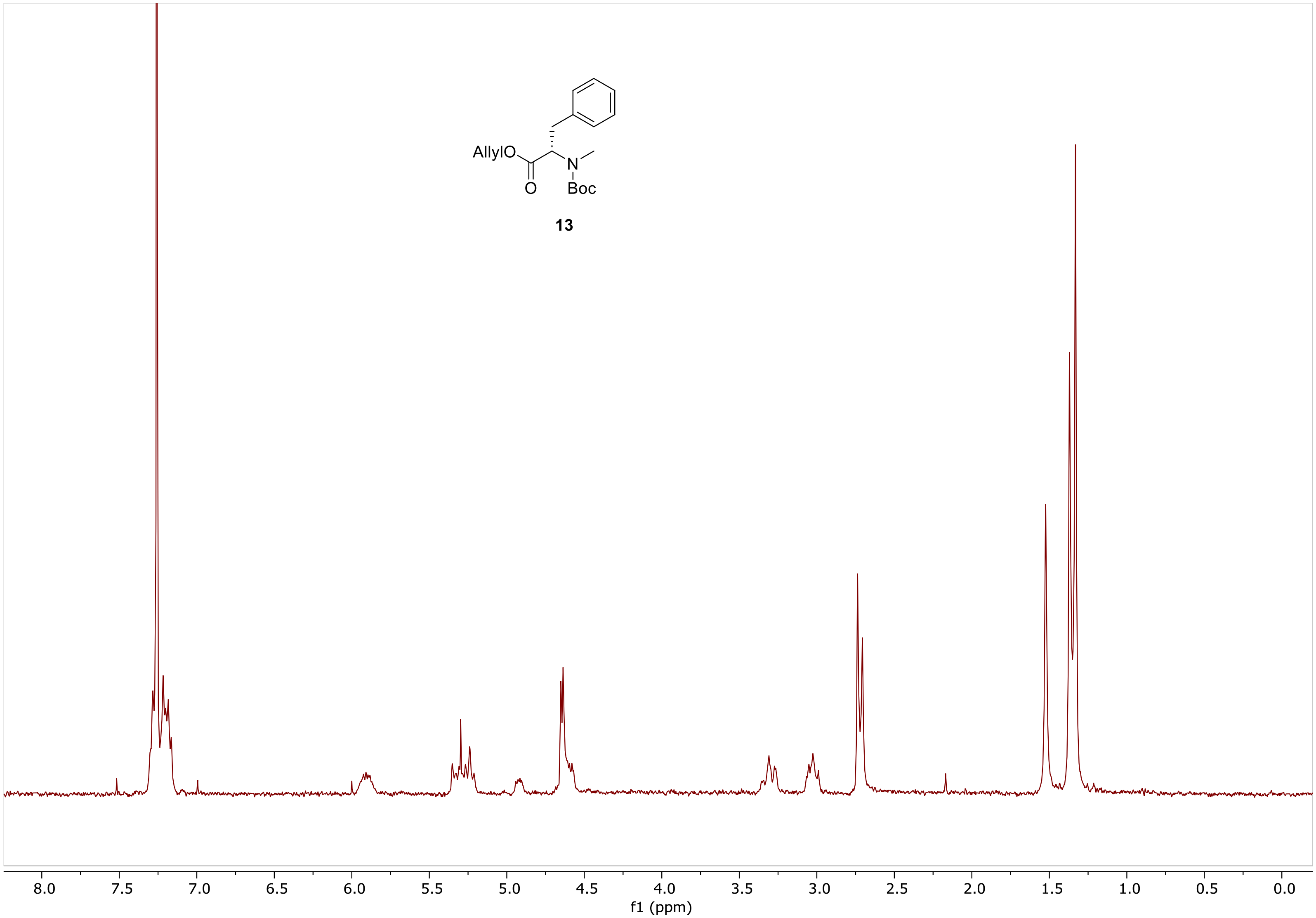

Figure S34. ^{13}C NMR spectrum of allyl *N*-(*tert*-butoxycarbonyl)-*N*-methyl-L-phenylalaninate (**13**) (100 MHz, $CDCl_3$).

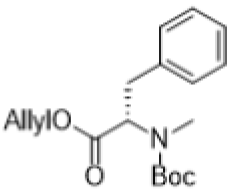

13

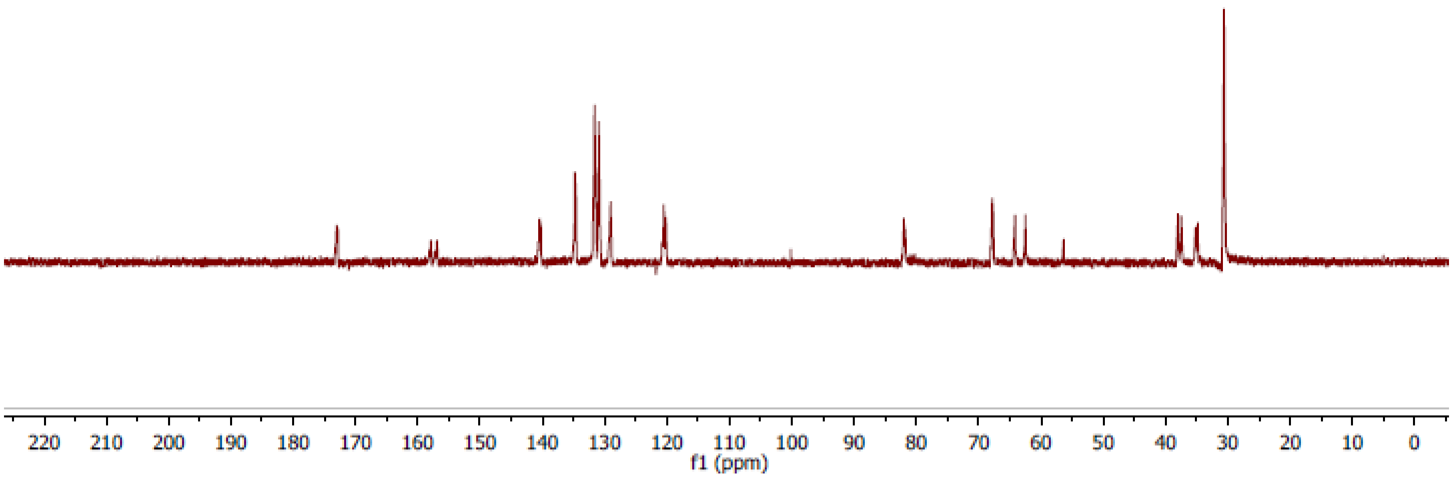

Figure S35. ^{1}H NMR spectrum of allyl methyl-L-phenylalaninate (**14**) (400 MHz, $CDCl_3$).

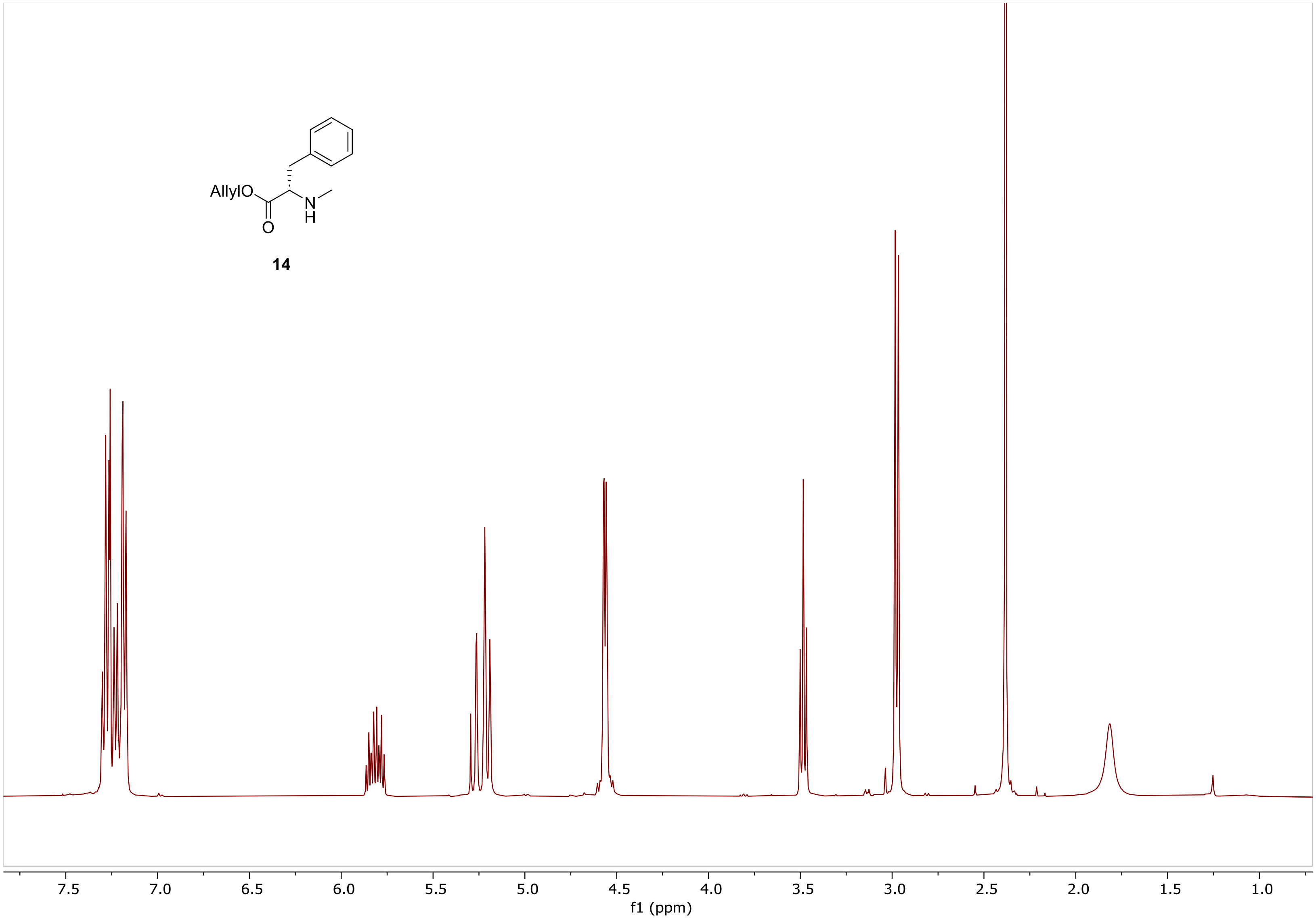

Figure S36. ^{13}C NMR spectrum of allyl methyl-L-phenylalaninate (**14**) (100 MHz, $CDCl_3$).

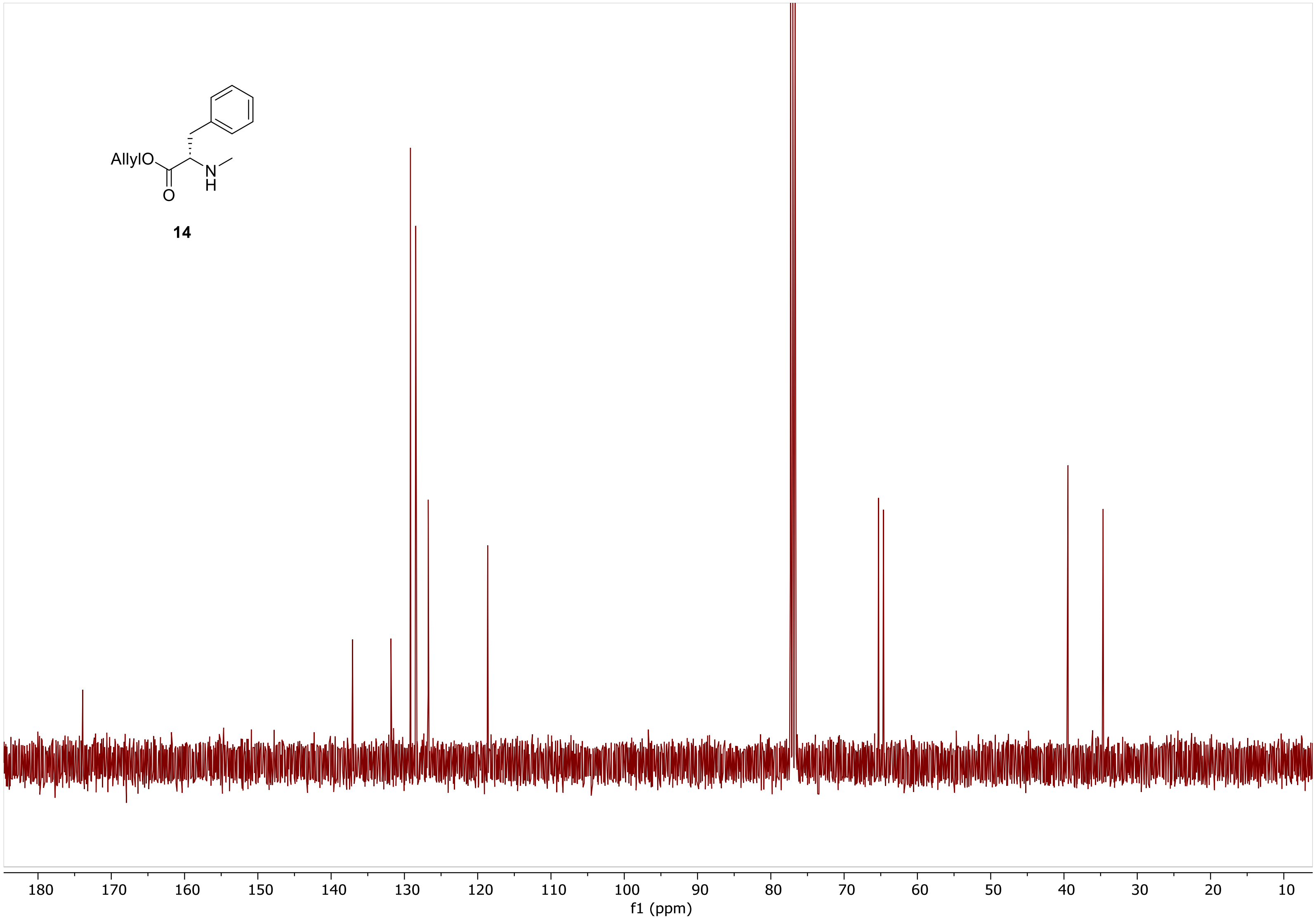

Figure S37. ^{1}H NMR spectrum of allyl *N*-(((*S*)-2-((*tert*-butyldimethylsilyl)oxy)-3-methylbutanoyl)-L-prolyl)-*N*-methyl-L-phenylalaninate (**15**) (500 MHz, $CDCl_3$).

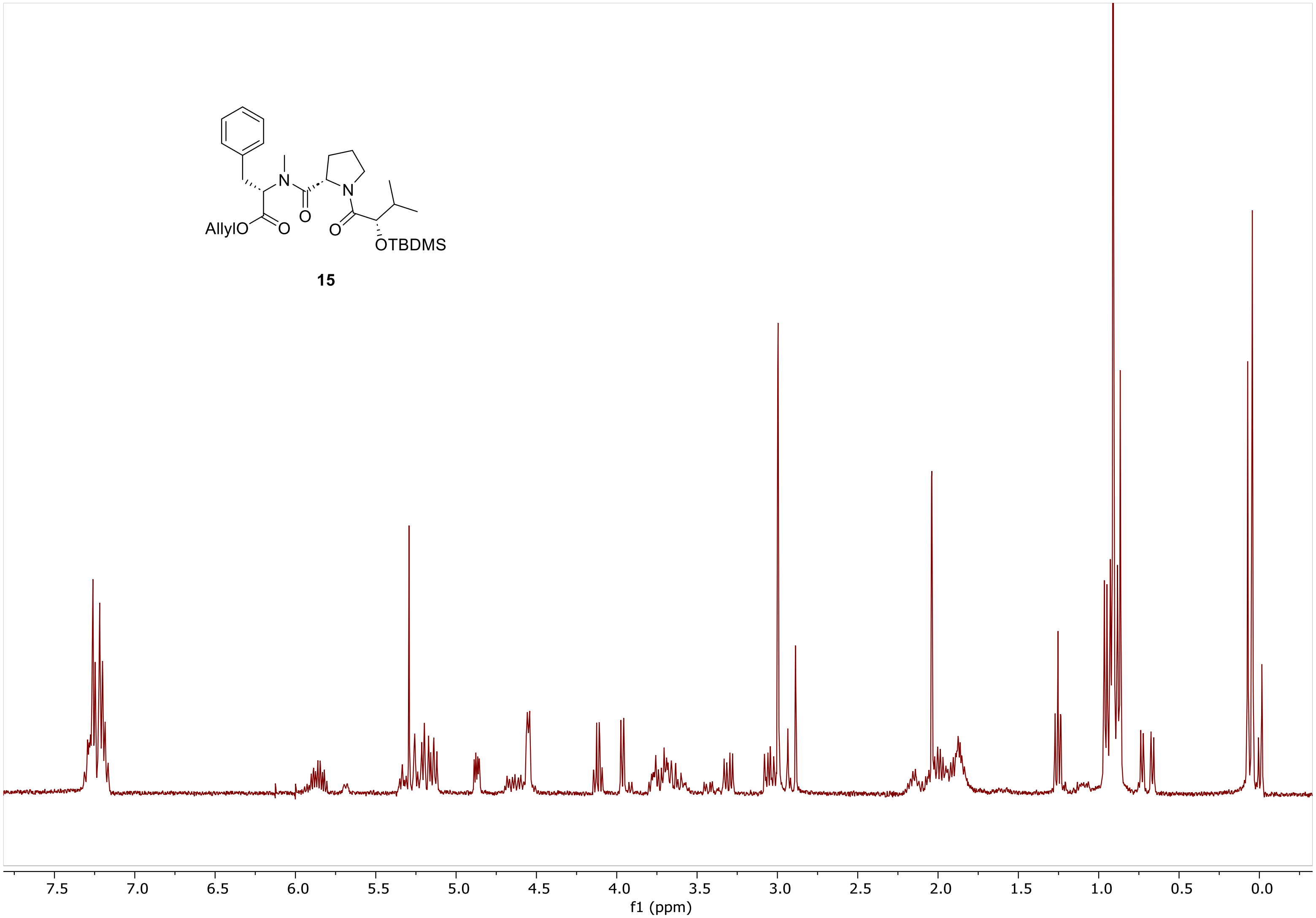

Figure S38. ^{13}C NMR spectrum of allyl *N*-(((*S*)-2-((*tert*-butyldimethylsilyl)oxy)-3-methylbutanoyl)-L-prolyl)-*N*-methyl-L-phenylalaninate (**15**) (100 MHz, $CDCl_3$).

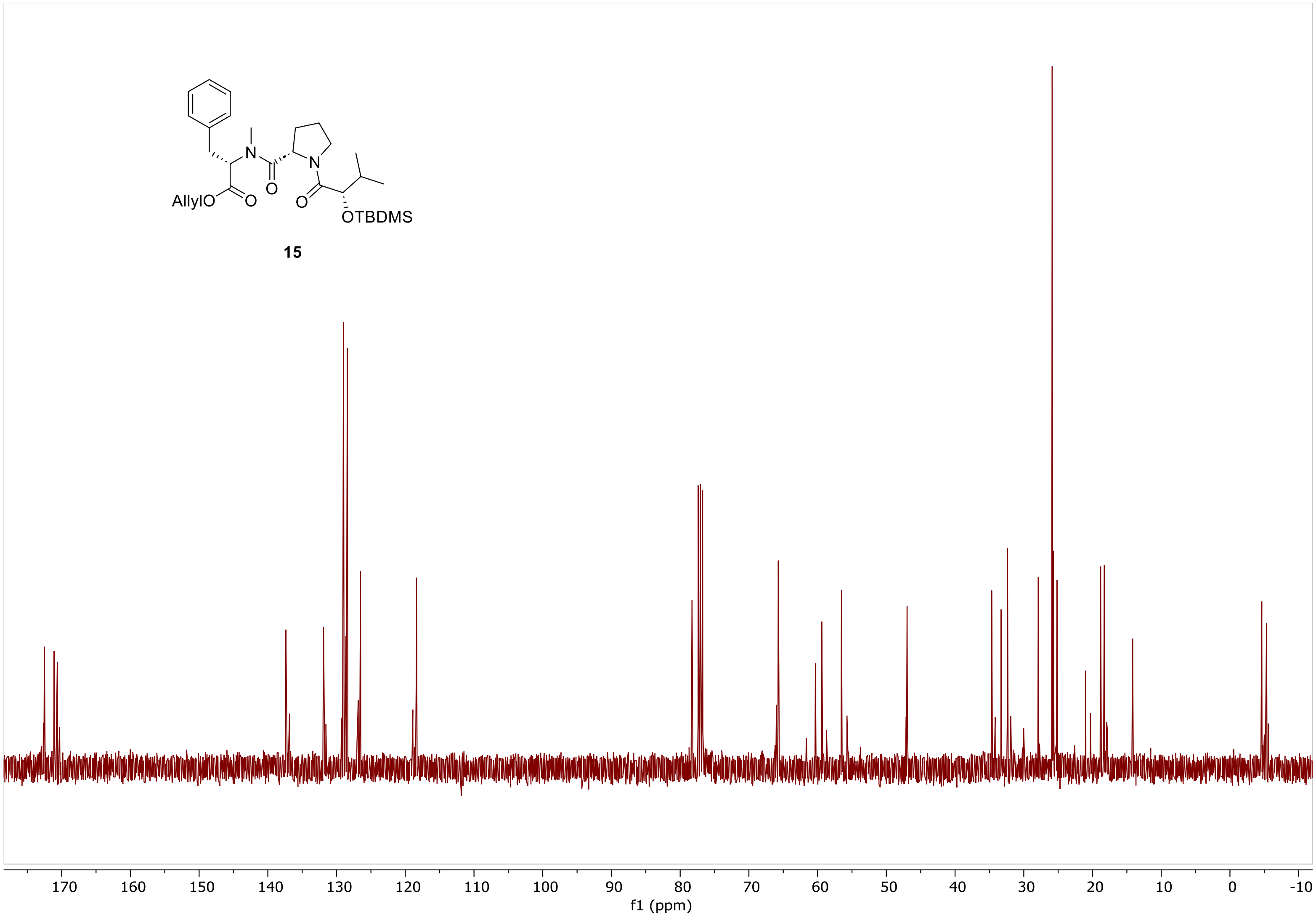

Figure S39. ^{1}H NMR spectrum of allyl *N*-(((*S*)-2-hydroxy-3-methylbutanoyl)-L-prolyl)-*N*-methyl-L-phenylalaninate (**3**) (500 MHz, $CDCl_3$).

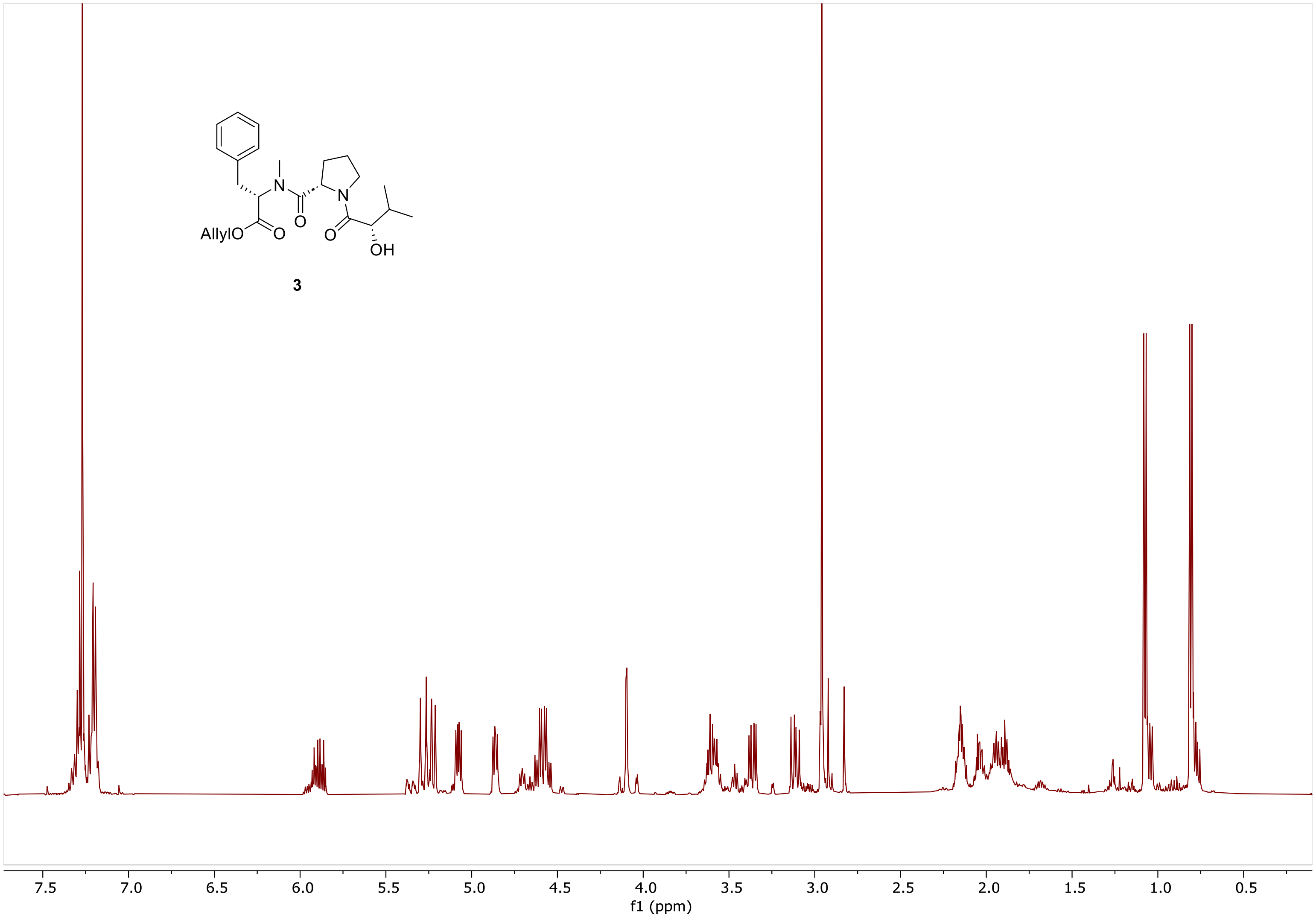

Figure S40. ^{13}C NMR spectrum of allyl *N*-(((*S*)-2-hydroxy-3-methylbutanoyl)-L-prolyl)-*N*-methyl-L-phenylalaninate (**3**) (100 MHz, $CDCl_3$).

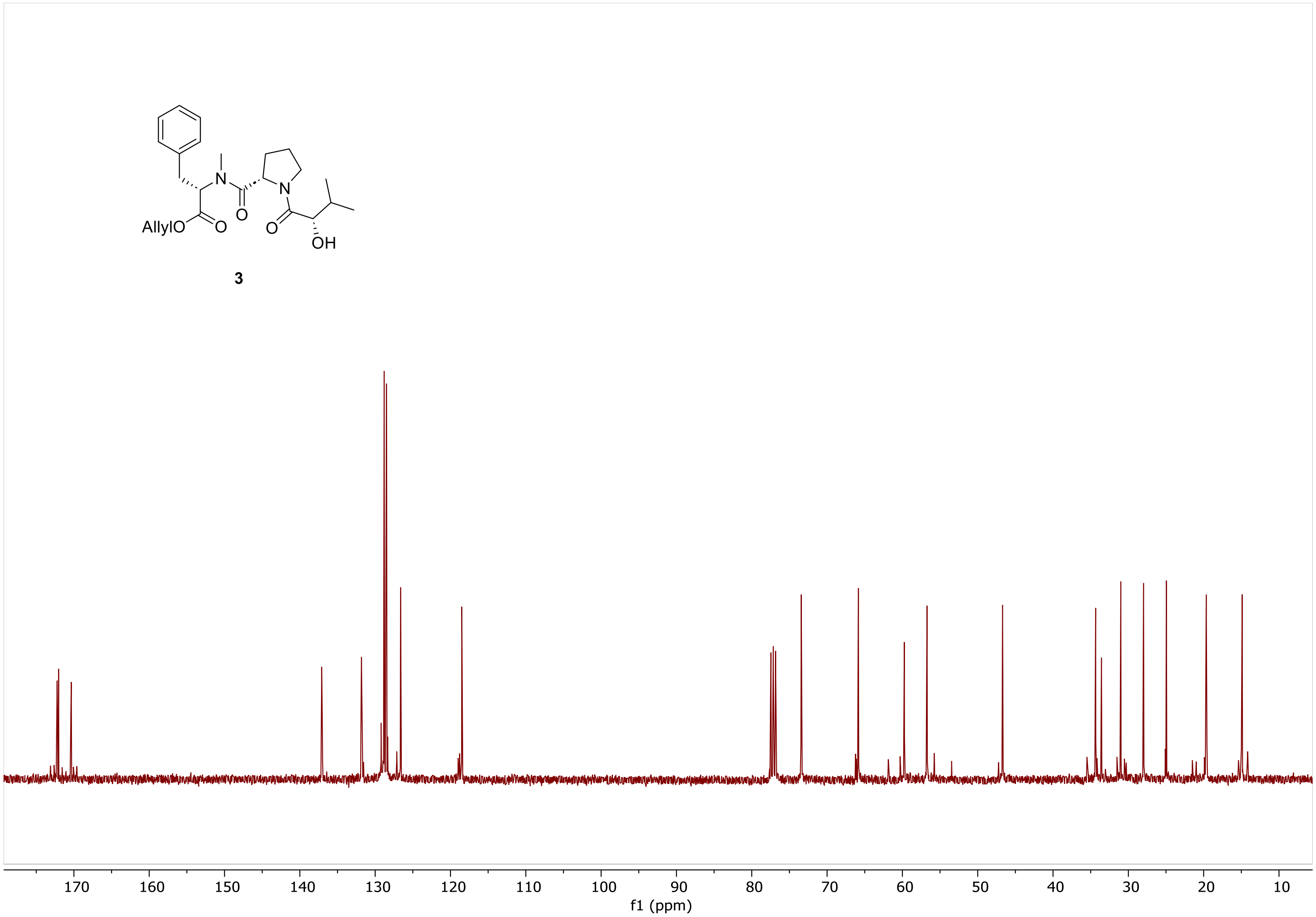

Figure S41. ^{1}H NMR spectrum of (*S*)-1-((*S*)-2-(((*S*)-1-(allyloxy)-1-oxo-3-phenylpropan-2-yl)(methyl)carbamoyl)pyrrolidin-1-yl)-3-methyl-1-oxobutan-2-yl (*S*)-3-((tert-butyldimethylsilyl)oxy)-2,2-dimethyloct-7-ynoate (**16**) (400 MHz, $CDCl_3$).

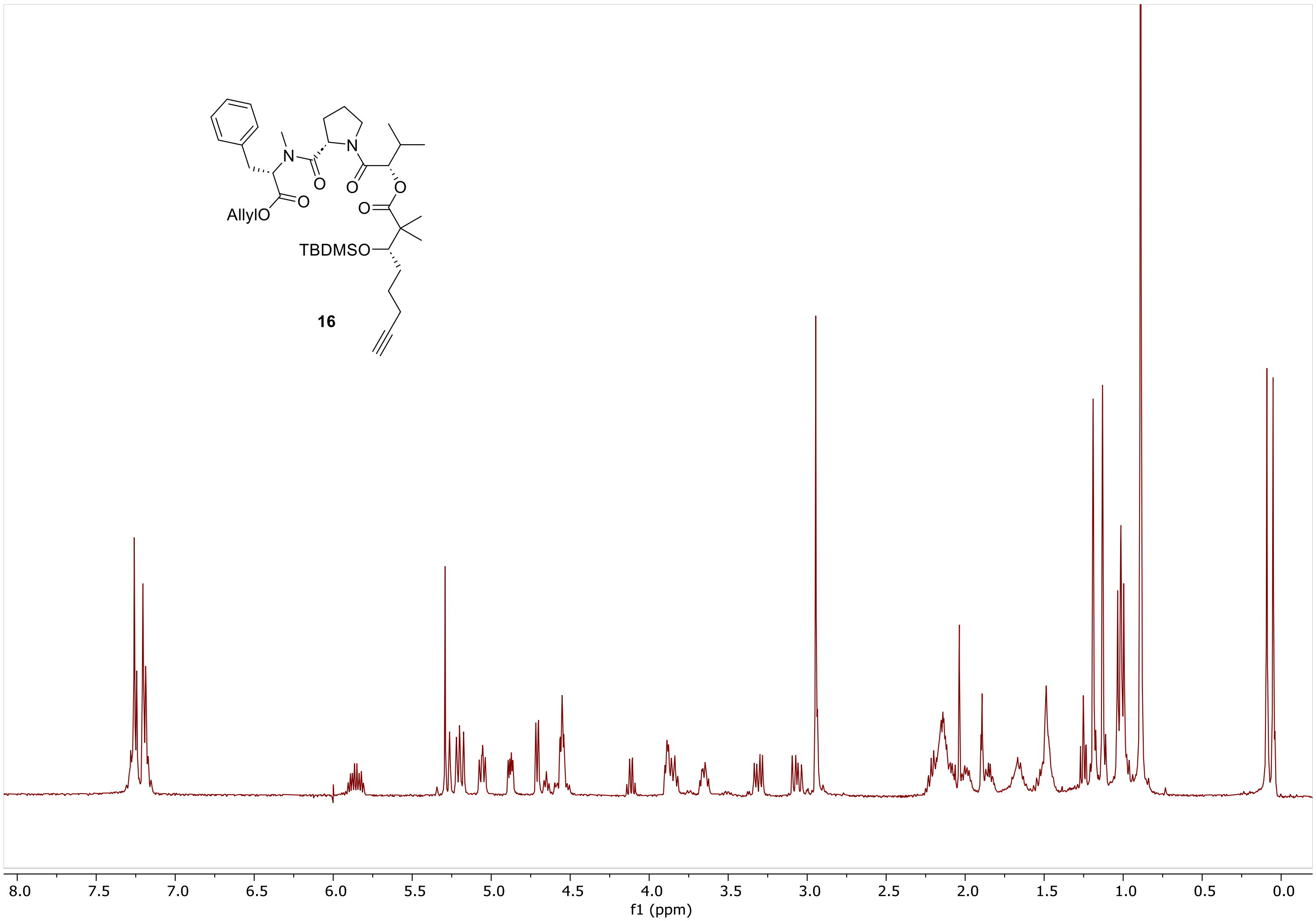

Figure S42. ^{13}C NMR spectrum of (*S*)-1-((*S*)-2-(((*S*)-1-(allyloxy)-1-oxo-3-phenylpropan-2-yl)(methyl)carbamoyl)pyrrolidin-1-yl)-3-methyl-1-oxobutan-2-yl (*S*)-3-((tert-butyldimethylsilyl)oxy)-2,2-dimethyloct-7-ynoate (**16**) (100 MHz, $CDCl_3$).

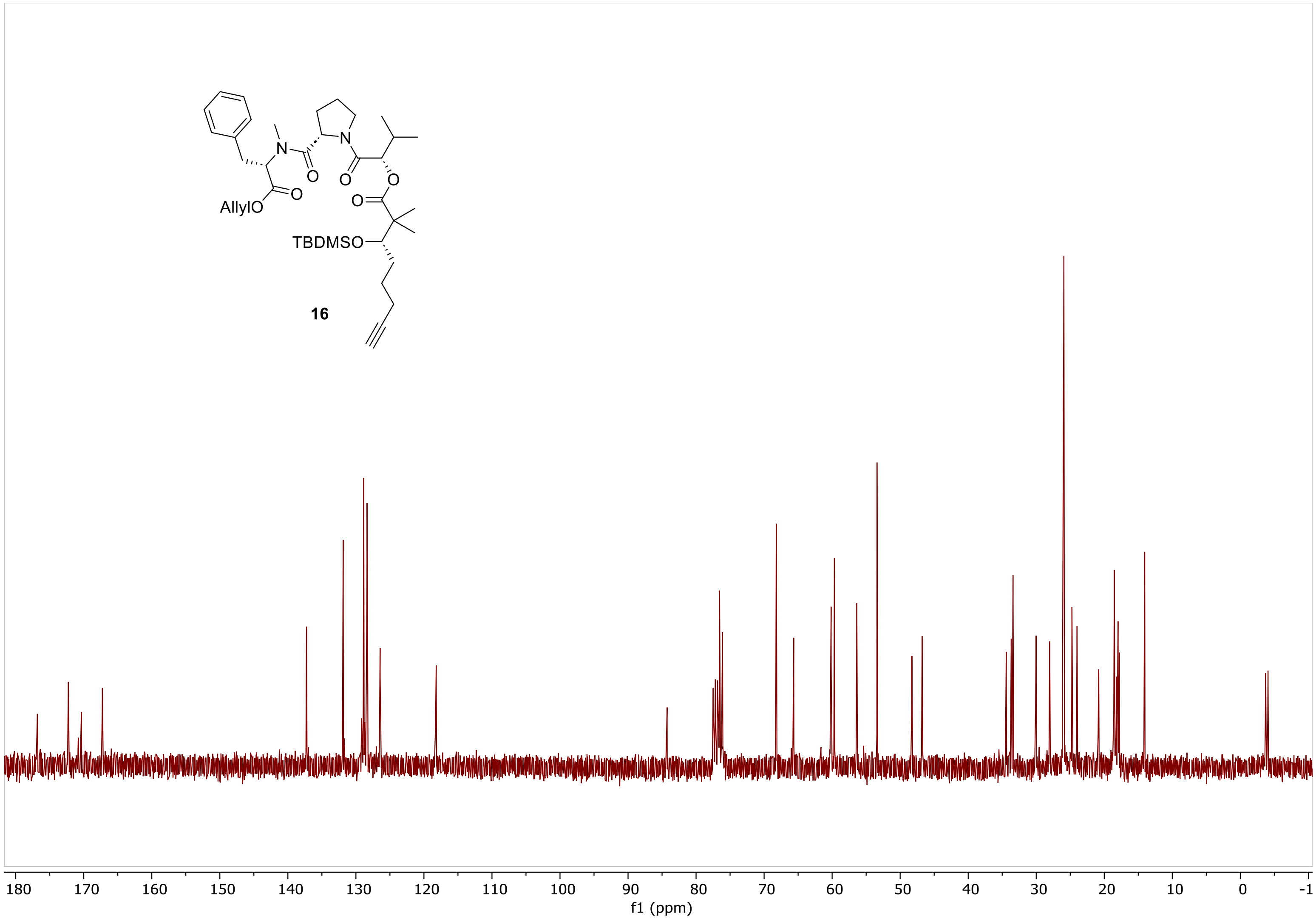

Figure S43. ^{1}H NMR spectrum of (*S*)-1-((*S*)-2-(((*S*)-1-(allyloxy)-1-oxo-3-phenylpropan-2-yl)(methyl)carbamoyl)pyrrolidin-1-yl)-3-methyl-1-oxobutan-2-yl (*S*)-3-hydroxy-2,2-dimethyloct-7-ynoate (**17**) (400 MHz, $CDCl_3$).

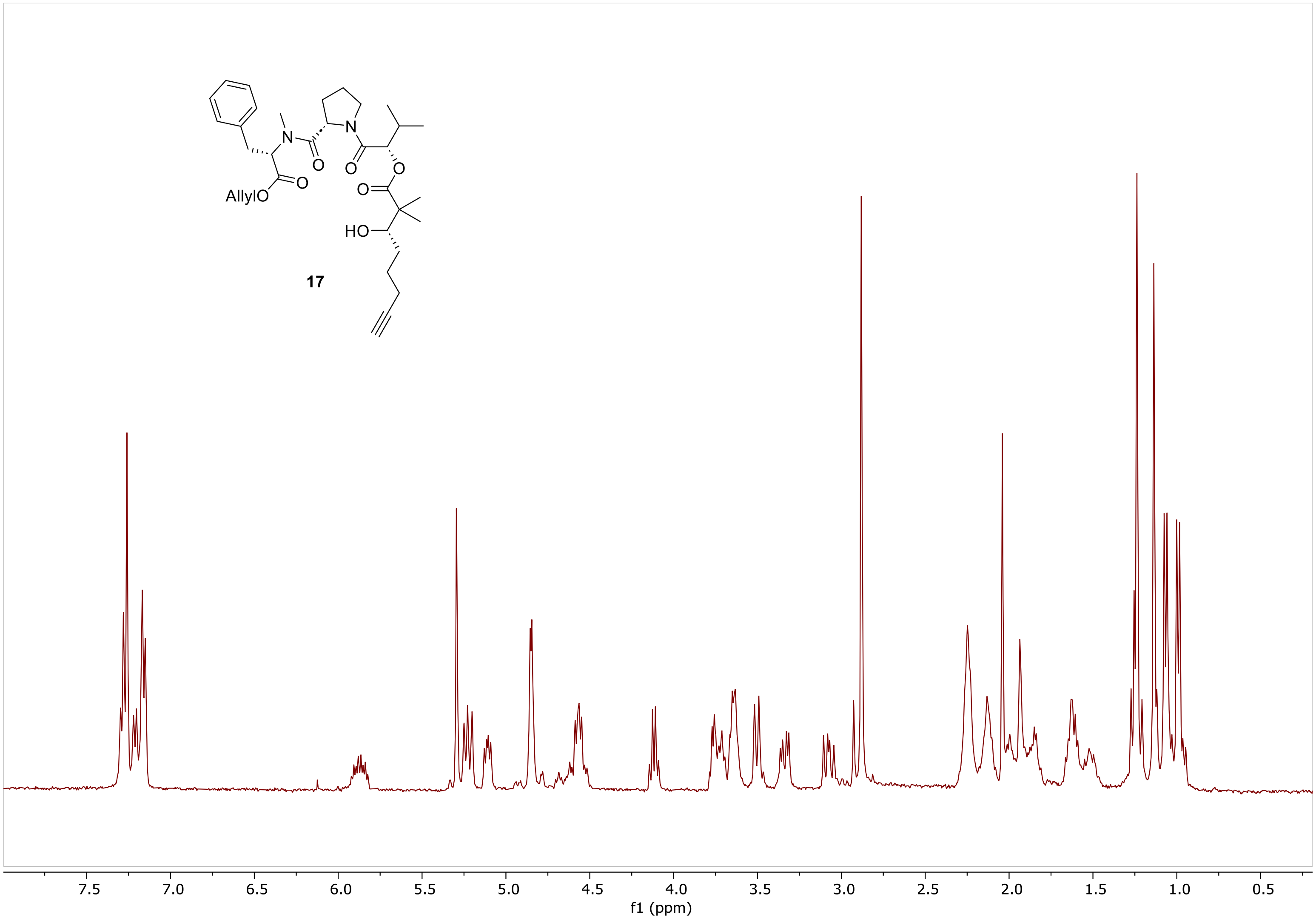

Figure S44. ^{13}C NMR spectrum of (*S*)-1-((*S*)-2-(((*S*)-1-(allyloxy)-1-oxo-3-phenylpropan-2-yl)(methyl)carbamoyl)pyrrolidin-1-yl)-3-methyl-1-oxobutan-2-yl (*S*)-3-hydroxy-2,2-dimethyloct-7-ynoate (**17**) (100 MHz, $CDCl_3$).

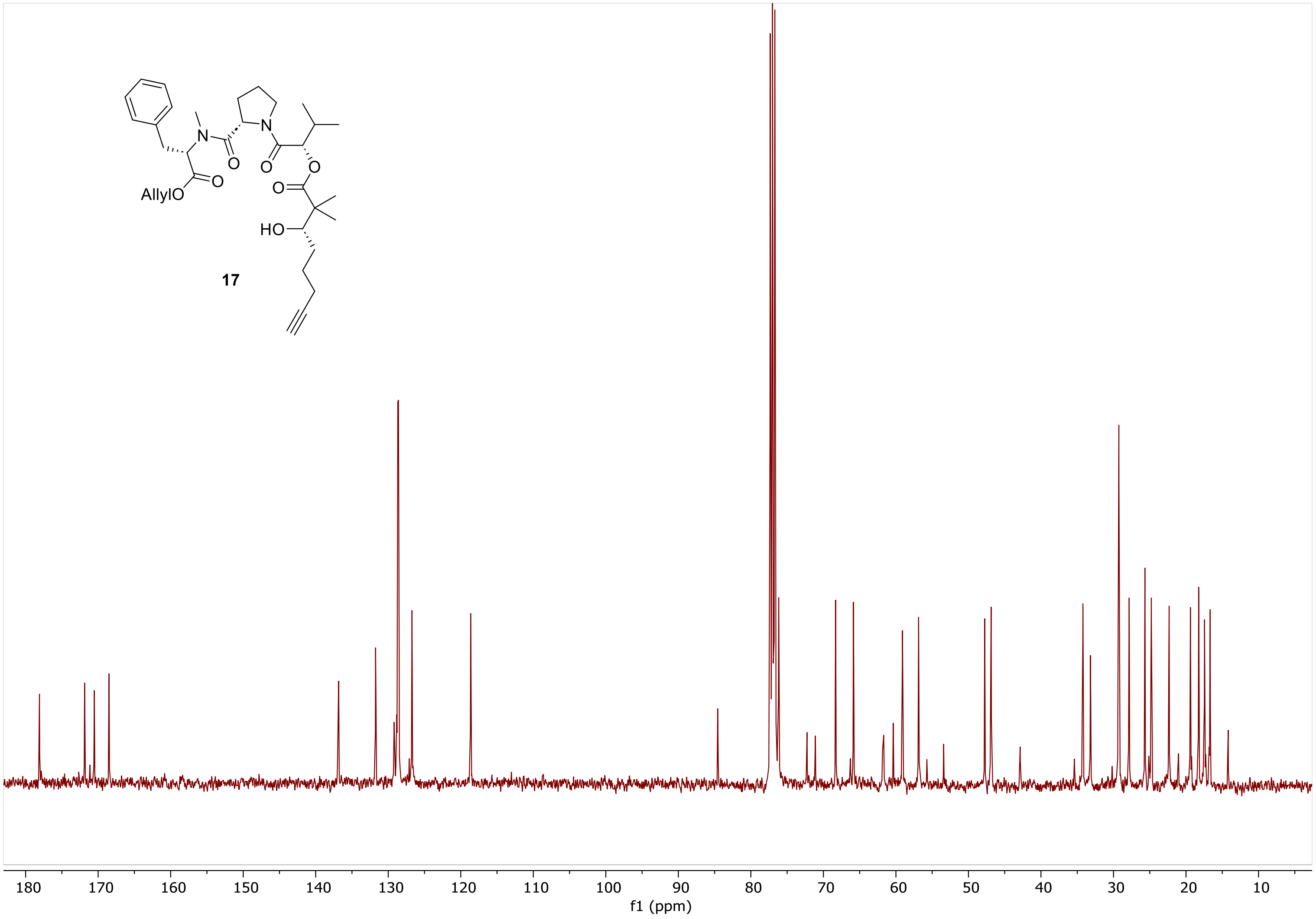

Figure S45. ^{1}H NMR spectrum of (*S*)-1-((*S*)-2-(((*S*)-1-(allyloxy)-1-oxo-3-phenylpropan-2-yl)(methyl)carbamoyl)pyrrolidin-1-yl)-3-methyl-1-oxobutan-2-yl (*S*)-3-((3-((*tert*-butoxycarbonyl)amino)propanoyl)oxy)-2,2-dimethyloct-7-ynoate (**18**) (400 MHz, $CDCl_3$).

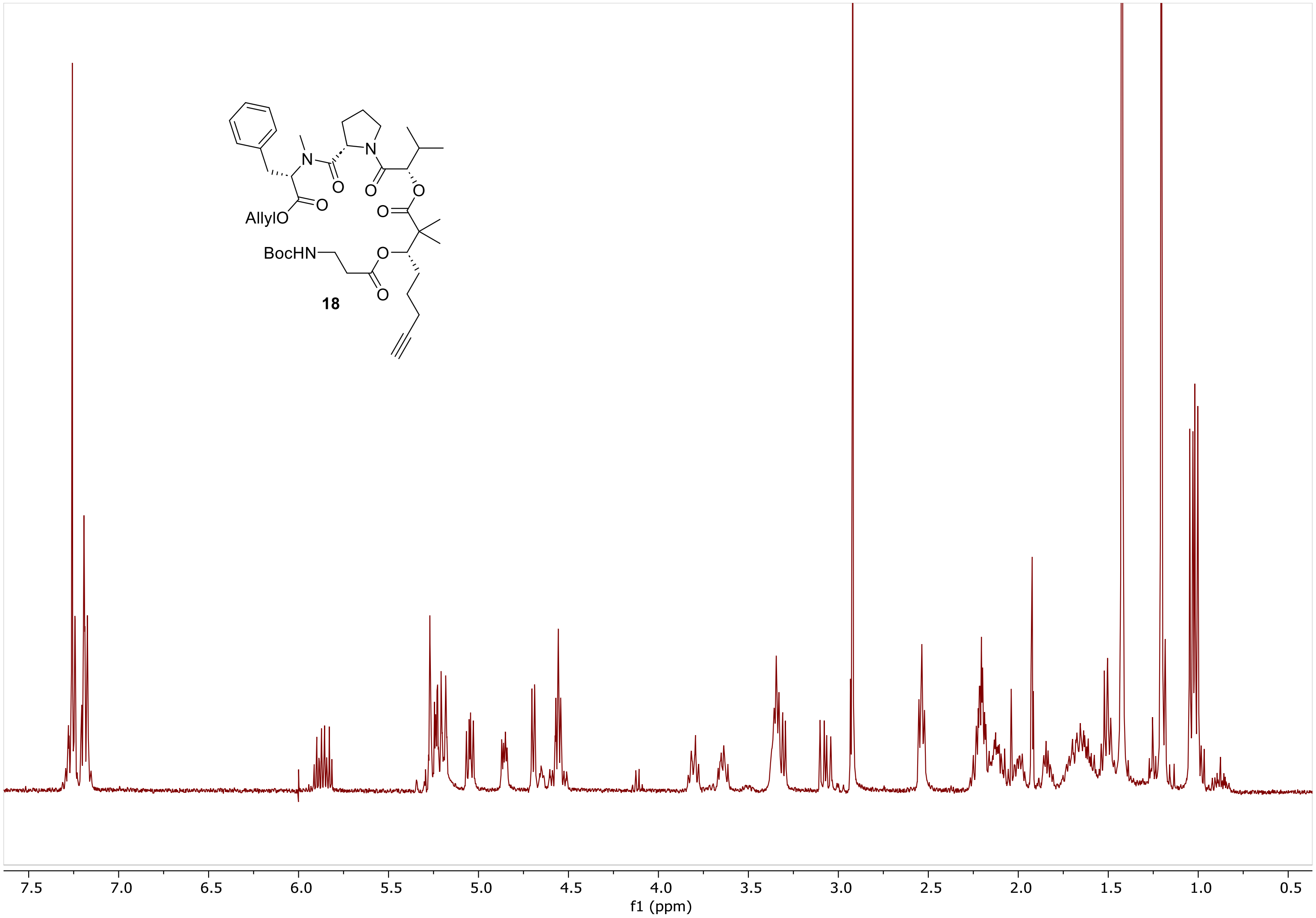

Figure S46. ^{13}C NMR spectrum of (*S*)-1-((*S*)-2-(((*S*)-1-(allyloxy)-1-oxo-3-phenylpropan-2-yl)(methyl)carbamoyl)pyrrolidin-1-yl)-3-methyl-1-oxobutan-2-yl (*S*)-3-((3-((*tert*-butoxycarbonyl)amino)propanoyl)oxy)-2,2-dimethyloct-7-ynoate (**18**) (100 MHz, $CDCl_3$).

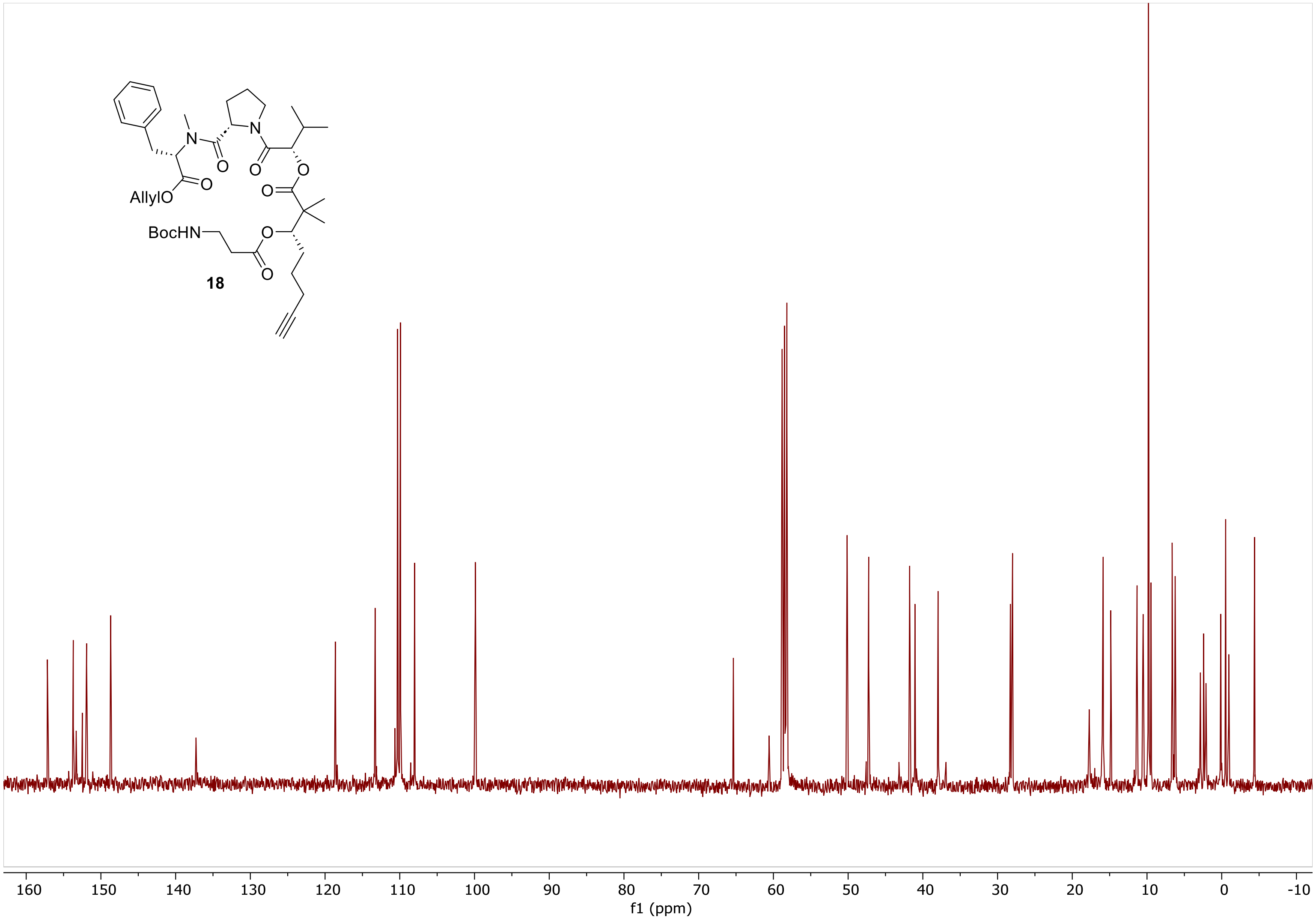

Figure S47. ^{1}H NMR spectrum of *N*-(((10*S*,14*S*)-14-isopropyl-2,2,11,11-tetramethyl-4,8,12-trioxo-10-(pent-4-yn-1-yl)-3,9,13-trioxa-5-azapentadecan-15-oyl)-L-prolyl)-*N*-methyl-L-phenylalanine (**19**) (400 MHz, $CDCl_3$).

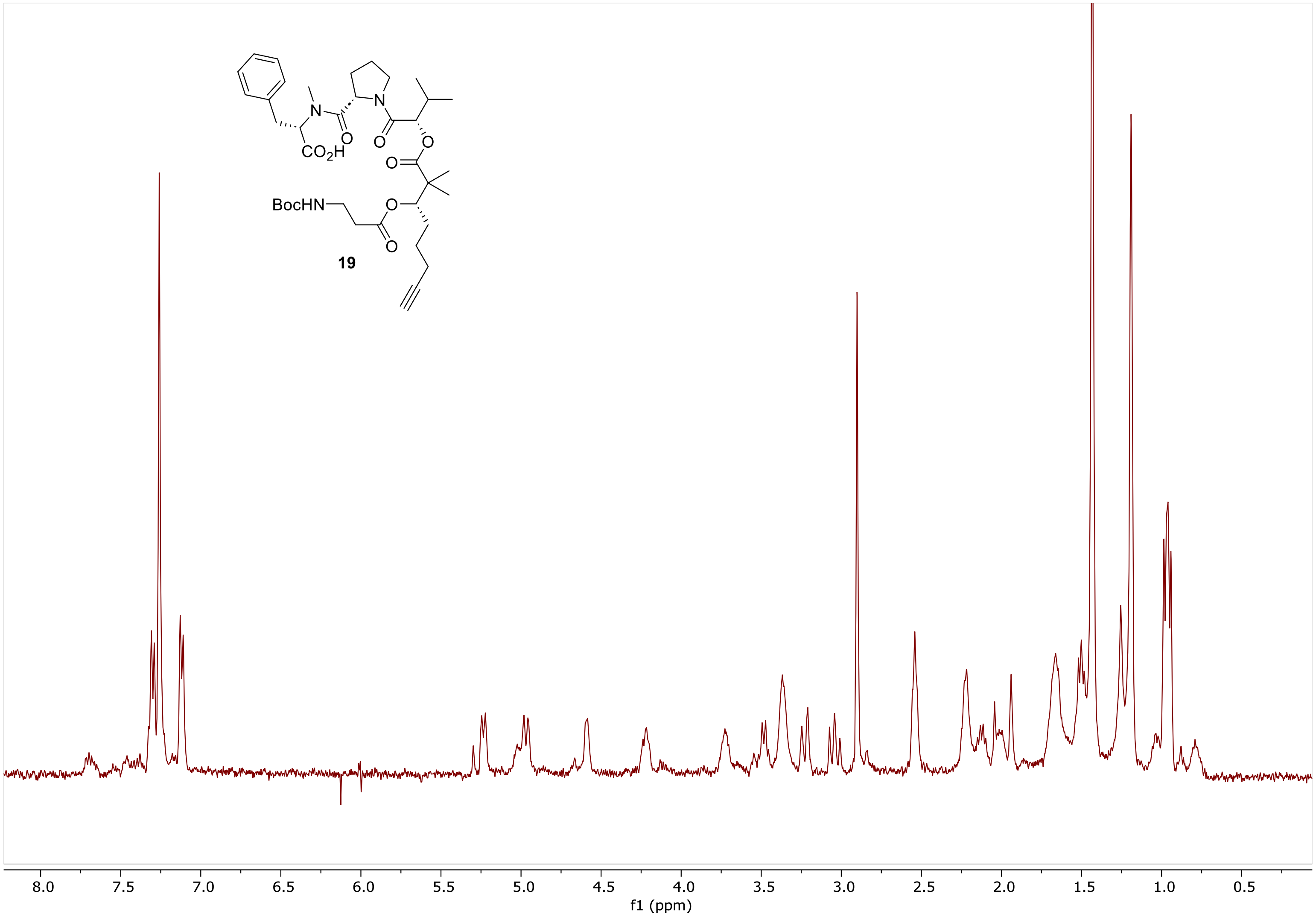

Figure S48. ^{13}C NMR spectrum of *N*-(((10*S*,14*S*)-14-isopropyl-2,2,11,11-tetramethyl-4,8,12-trioxo-10-(pent-4-yn-1-yl)-3,9,13-trioxa-5-azapentadecan-15-oyl)-L-prolyl)-*N*-methyl-L-phenylalanine (**19**) (100 MHz, CD_3OD).

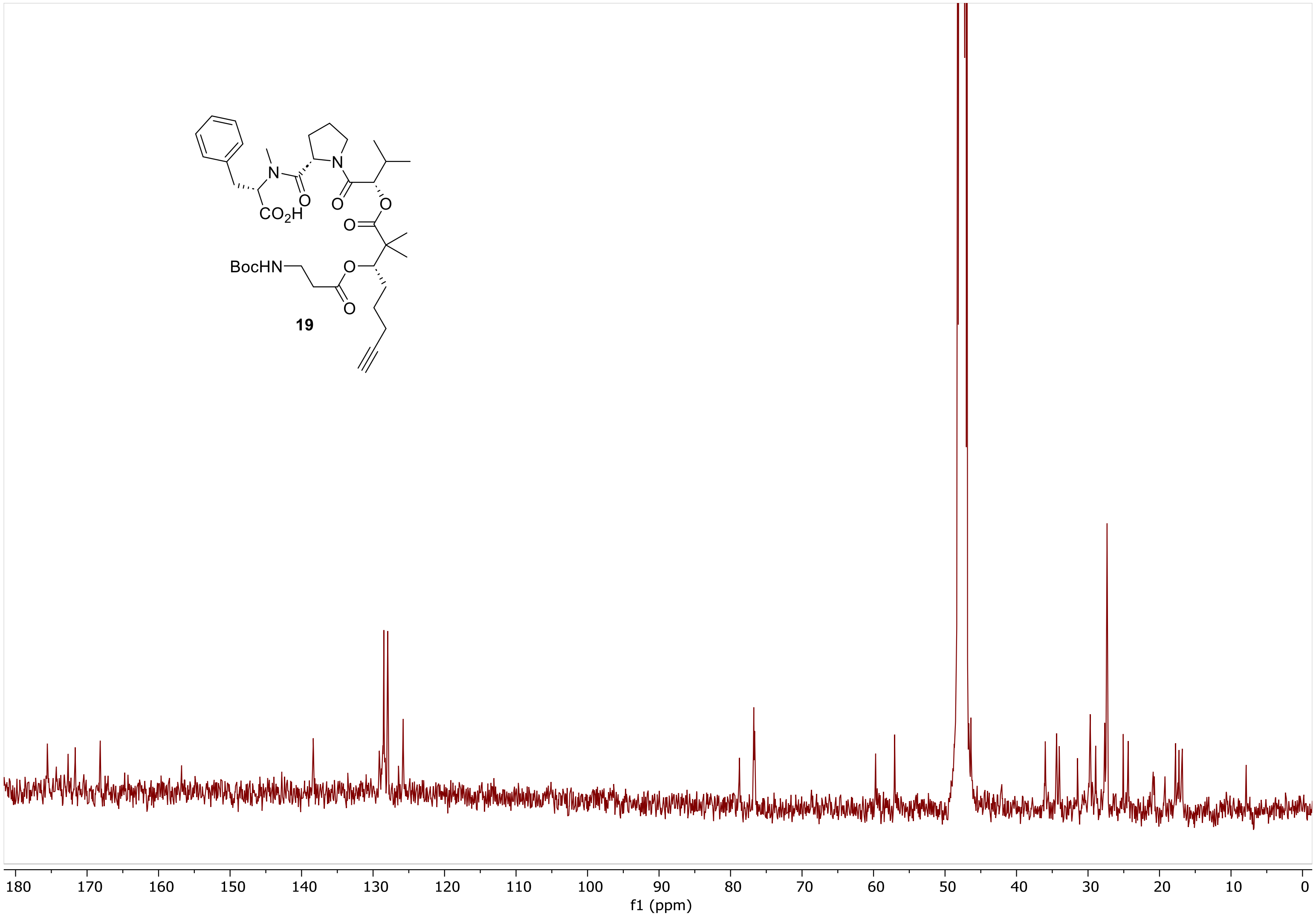

Figure S49. ^{1}H NMR spectrum of *N*-(((*S*)-2-(((S)-3-((3-aminopropanoyl)oxy)-2,2-dimethyloct-7-ynoyl)oxy)-3-methylbutanoyl)-L-prolyl)-*N*-methyl-L-phenylalanine (**20**) (400 MHz, CD_3OD).

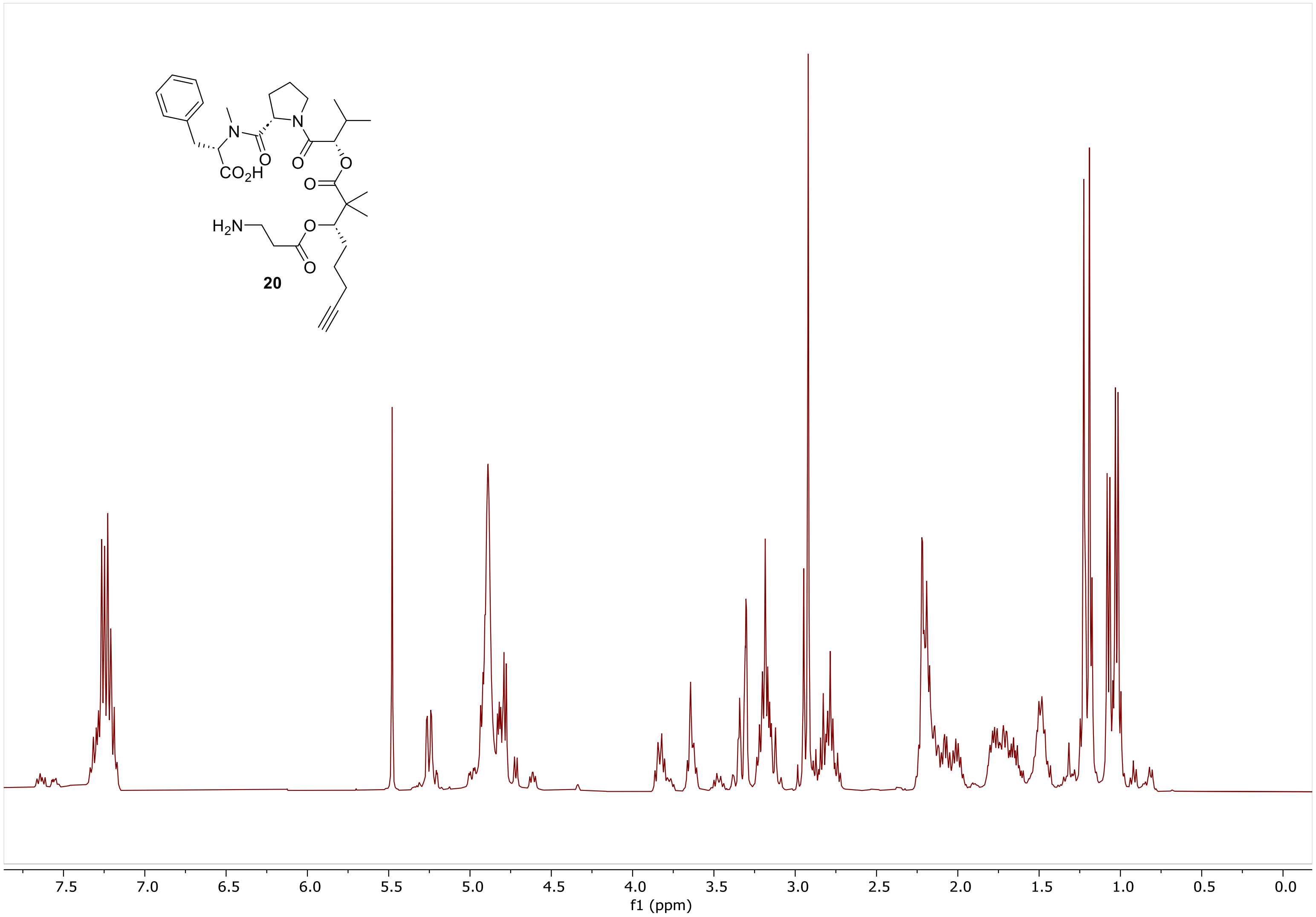

Figure S50. ^{13}C NMR spectrum of *N*-(((*S*)-2-(((S)-3-((3-aminopropanoyl)oxy)-2,2-dimethyloct-7-ynoyl)oxy)-3-methylbutanoyl)-L-prolyl)-*N*-methyl-L-phenylalanine (**20**) (100 MHz, CD_3OD).

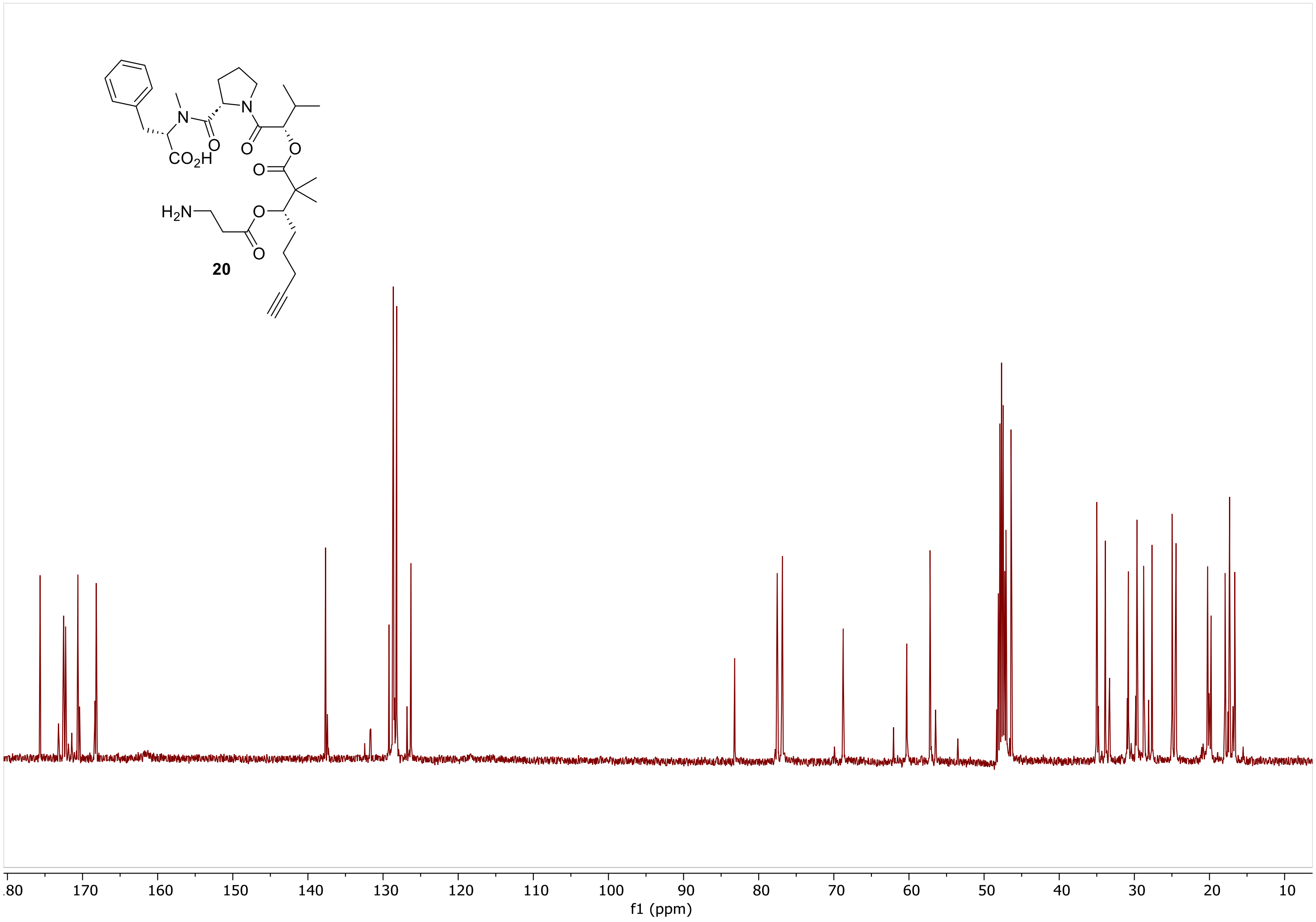

Figure S51. ^{1}H NMR spectra of synthetic *vs.* natural **PM170453**.

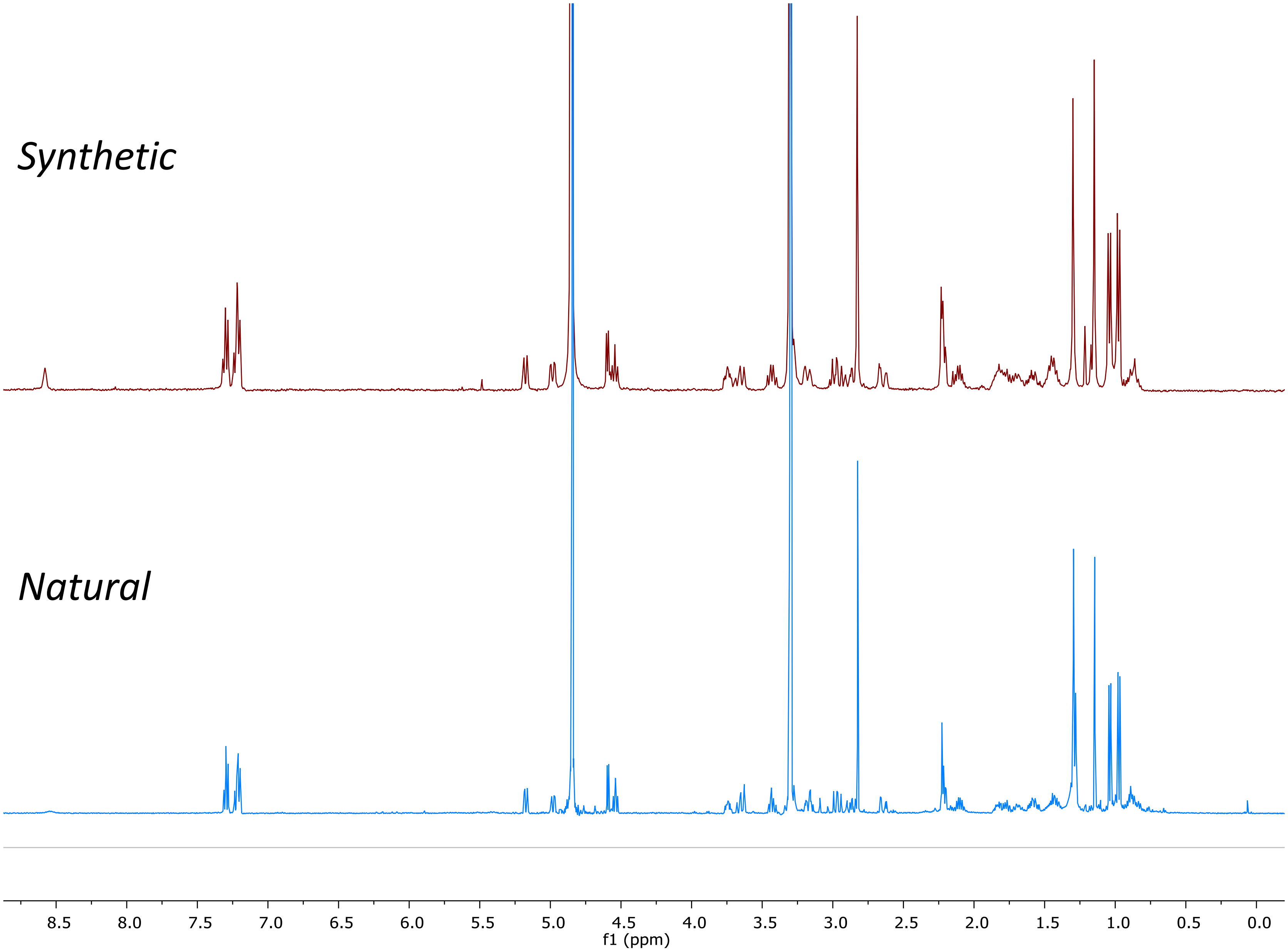

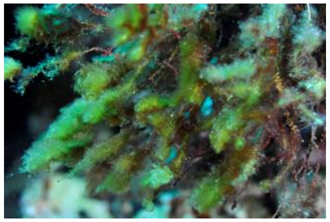

Supplement: Supplementary file 1 [file marinedrugs-22-00303-s001.zip › marinedrugs-3067401-supplementary.pdf]
